# A Dynamic 3D Graphical Representation for RNA Structure Analysis and Its Application in Non-Coding RNA Classification

**DOI:** 10.1371/journal.pone.0152238

**Published:** 2016-05-23

**Authors:** Yi Zhang, Haiyun Huang, Xiaoqing Dong, Yiliang Fang, Kejing Wang, Lijuan Zhu, Ke Wang, Tao Huang, Jialiang Yang

**Affiliations:** 1 Department of Mathematics, Hebei University of Science and Technology, Shijiazhuang, Hebei 050018, People's Republic of China; 2 Hebei Laboratory of Pharmaceutic Molecular Chemistry, Shijiazhuang, Hebei 050018, People's Republic of China; 3 Department of Information Retrieval of Library, Hebei University of Science and Technology, Shijiazhuang, Hebei 050018, People's Republic of China; 4 International Travel Healthcare Center, Fuzhou, Fujian 350001, People's Republic of China; 5 Institute of Health Sciences, Shanghai Institutes for Biological Sciences, Chinese Academy of Sciences, Shanghai 200031, People's Republic of China; 6 Department of Genetics and Genomic Sciences, Icahn School of Medicine at Mount Sinai, New York, NY 10029, United States of America; Tianjin University, CHINA

## Abstract

With the development of new technologies in transcriptome and epigenetics, RNAs have been identified to play more and more important roles in life processes. Consequently, various methods have been proposed to assess the biological functions of RNAs and thus classify them functionally, among which comparative study of RNA structures is perhaps the most important one. To measure the structural similarity of RNAs and classify them, we propose a novel three dimensional (3D) graphical representation of RNA secondary structure, in which an RNA secondary structure is first transformed into a characteristic sequence based on chemical property of nucleic acids; a dynamic 3D graph is then constructed for the characteristic sequence; and lastly a numerical characterization of the 3D graph is used to represent the RNA secondary structure. We tested our algorithm on three datasets: (1) Dataset I consisting of nine RNA secondary structures of viruses, (2) Dataset II consisting of complex RNA secondary structures including pseudo-knots, and (3) Dataset III consisting of 18 non-coding RNA families. We also compare our method with other nine existing methods using Dataset II and III. The results demonstrate that our method is better than other methods in similarity measurement and classification of RNA secondary structures.

## Introduction

As a bridge of information transmission, RNAs carry a wide variety of functions in biological systems, including performing catalytic function, regulating gene expression, and carrying genetic information [1]. In living cells, the single-stranded RNAs do not remain in a linear form. Instead, their bases always fold into pairs that lead to the formation of RNA secondary structures [2]. Since the three dimensional (3D) structures and functions of RNAs are mostly determined by their secondary structures [3], it is important to account for both primary sequences and secondary structures in understanding the functional similarities among RNAs, especially for non-coding RNAs (ncRNAs) and RNAs with pseudo-knots.

Recently, ncRNAs have become a focus for both computational and experimental researches [4]. Though ncRNAs do not encode proteins, they are involved in a lot of cellular processes [5]. For example, ncRNAs play an important role in chromosome maintenance and segregation [6] and have also been implicated in neurological diseases and various cancers [7, 8]. Specifically, microRNAs are endogenous, short, non-coding RNA molecules that are directly involved in the posttranscriptional regulation of gene expression. Dysregulation of microRNAs is usually associated with diseases [9, 10]. Moreover, a few computational methods have been developed to detect causal genes of diseases at the whole-genome level [11, 12].

It is known that the functions of ncRNAs are mostly determined by their structures [5]. Though sequences can be well aligned by techniques like seed technique [13, 14], predicting the secondary structure of ncRNAs is very difficult [15]. Novel and effective methods to accurately evaluate the structural similarities of ncRNAs are highly demanded, especially for those with special structures like pseudo-knot. As a kind of RNA structures formed by stem nesting, pseudo-knot is responsible for a few important biological activities such as virus infiltration [16].

Historically, dynamic programming based algorithms with various scoring functions have been widely used to measure the similarities among RNA secondary structures [17–19]. For example, based on the alignment of a string representation of RNA secondary structure, a score function and a distance function were established to represent insertion, deletion, and substitution of bases in the compared structures [17, 18]. In addition, a tree representation of the RNA secondary structure elements [19] and base pairing probability matrices [20, 21] were also proposed to measure the similarities among RNA structures. However, methods based on dynamic programming are computationally inefficient, which makes it hard to predict RNAs with complex secondary structures like pseudo-knot.

In order to measure RNA similarity more efficiently, various alternative techniques have been tested. For example, a novel 2D graphical representation of RNA secondary structure was proposed in [22]. However, this method may cause the loss of information due to its non-uniqueness in representing an RNA. Jeffery introduced a chaos game representation of DNA sequences [23], based on which Li et al. proposed a non-degenerative 2D graphical representation of RNA secondary structure to solve the information loss problem [24]. On the ground of sequence and base chemical information, two similar 3D representation methods were proposed [23, 25]. However, they are space-demanding, especially for long RNAs. As a high dimension representation scheme, a 4D method was developed to resolve the problem of structure degeneracy and information loss, but it is not good for visualization [26]. In addition, a novel wavelet-based graphical representation method was used to classify non-coding RNA secondary structures [27]. However, the data obtained by this method is redundant because each base is characterized by three vectors.

In this paper, we proposed a novel dynamic 3D graphical representation of RNA secondary structures based on their sequences, chemical properties, and structural information. We evaluated our algorithm on three sets of RNA secondary structures including 9 viral RNAs, 33 RNAs with complex secondary structures, and 120 non-coding RNAs. A comparison study showed that our novel method outperformed other nine methods in deciphering RNA similarity.

## Materials and Methods

### Data

**Fig 1:**
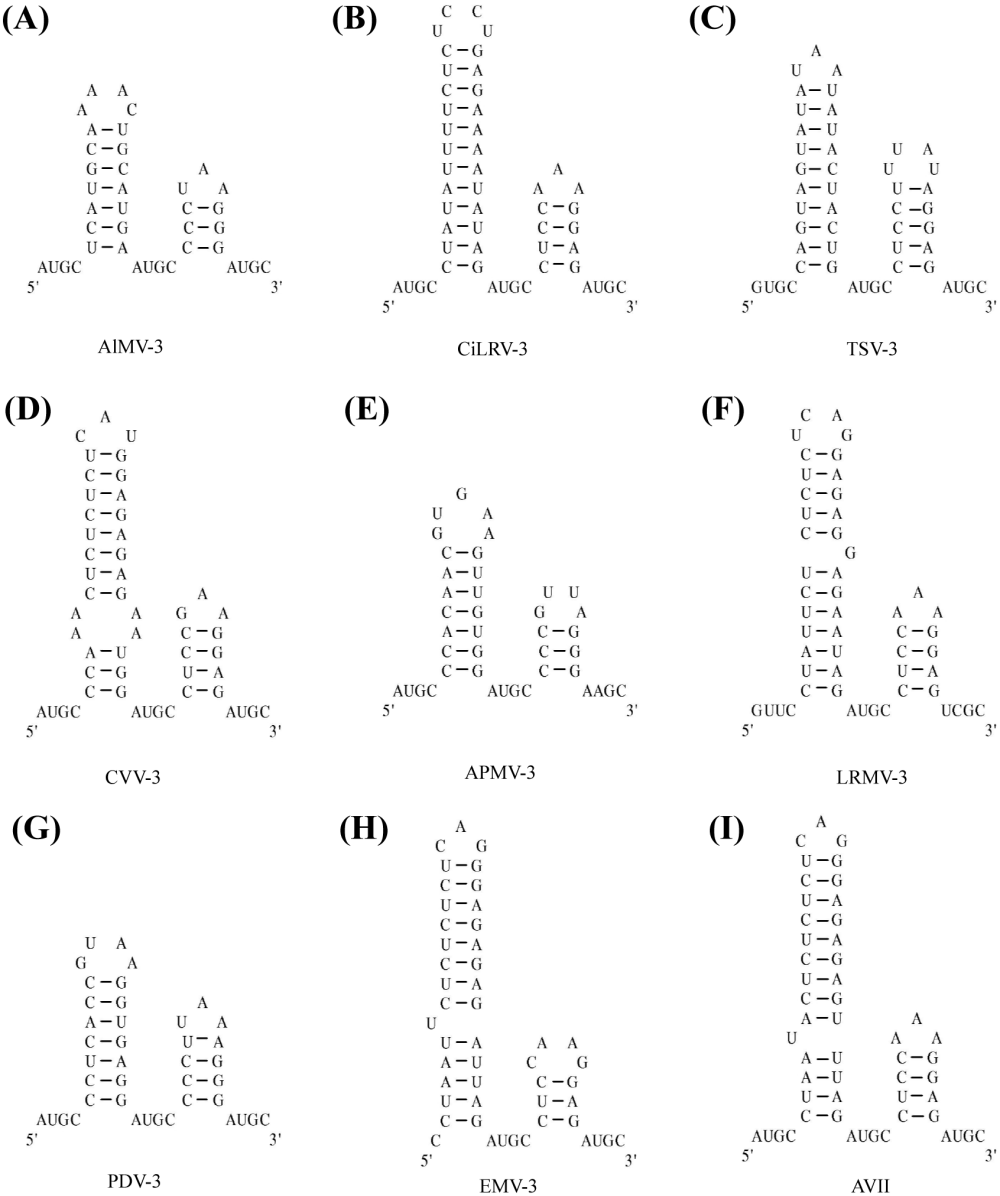
The RNA secondary structures at the 3′—terminus for 9 viruses. (A) alfalfa mosaic virus AlMV-3, (B) citrus leaf rugose virus CiLRV-3, (C) tobacco streak virus TSV-3, (D) citrus variegation virus CVV-3, (E) apple mosaic virus APMV-3, (F) lilac ring mottle virus LRMV-3, (G) prune dwarf ilarvirus PDV-3, (H) elm mottle virus EMV-3, and (I) asparagus virus 2 AVII.

We adopted three RNA datasets in this study: (1) Dataset I consisting of 3'-terminus of RNAs from nine viruses reported by Bol (see Fig 1) [28]; (2) Dataset II containing two kinds of RNA secondary structures: 17 complex RNA secondary structures retrieved from RNase P database [29] and 16 RNA secondary structures with pseudo-knots retrieved from Pseud Base (Pseud Base++) [30] (see S1 Fig); (3) Dataset III including 60 non-coding RNA secondary structures from RNA STRAND database (RNA STRAND v2.0) [31, 32], and 60 non-coding RNA sequences randomly selected from 18 non-coding RNA families in Rfam database (see S2 Fig).

### 3D graphical representation of RNA secondary structures

The secondary structure of an RNA was constructed by four free bases A, G, C, and U, as well as base pairs formed by bonds between A-U, G-C, and G-U. For convenience, A, G, C, and U located in the base pairs were denoted by A', G', C', and U', respectively. In this way, we can use a sequence consisting of A, G, C, U and A', G', C', U' to represent an RNA secondary structure. The sequence is called the “characteristic sequence” of the RNA secondary structure. We provided a software to generate such sequence (see S1 Software) from any RNA secondary structure.

Based on their chemical properties, the four bases A, C, G, and U can be divided into three groups: (i) amino M = {A,C} and keto K = {G,U}, (ii) purine R = {A,G} and pyrimidine Y = {C,U}, and (iii) weak H-bonds W = {A,U} and strong H-bonds S = {C,G}. According to the base classification scheme (i), (ii), and (iii), a characteristic sequence can be represented by three 3D graphs through three maps *φ*_1_, *φ*_2_, and *φ*_3_ respectively (see Table 1), where *A*_*i*_, *G*_*i*_, *C*_*i*_, *U*_*i*_, ${A^{\prime }}_{i}$, ${G^{\prime }}_{i}$, ${C^{\prime }}_{i}$, and ${U^{\prime }}_{i}$ are the cumulative occurrence numbers of *A*, *G*, *C*, *U*, *A*′, *G*′, *C*′, and *U*′, respectively in the characteristic sequence from the first base to the *i*-th base, *i* = 1, 2, … *n* with *n* being the length of the characteristic sequence.

**Table 1:** The definition of three maps *φ*_1_, *φ*_2_, and *φ*_3_.

*g*_*i*_	*φ*_1_(*g*_*i*_) = (*x*_1*i*_, *y*_1*i*_, *z*_1*i*_)			*g*_*i*_	*φ*_2_(*g*_*i*_) = (*x*_2*i*_, *y*_2*i*_, *z*_2*i*_)			*g*_*i*_	*φ*_3_(*g*_*i*_) = (*x*_3*i*_, *y*_3*i*_, *z*_3*i*_)		
	*x*_1i_	*y*_1i_	*z*_1i_		*x*_2i_	*y*_2i_	*z*_2i_		*x*_3i_	*y*_3i_	*z*_3i_
{*AorC*}	$\frac{i}{n+1}$	$\sqrt{1-{\left(\frac{i}{n+1}\right)}^{2}}$	*A*_*i*_+*C*_*i*_	{*AorG*}	$\frac{i}{n+1}$	$\sqrt{1-{\left(\frac{i}{n+1}\right)}^{2}}$	*A*_*i*_+*G*_*i*_	{*AorU*}	$\frac{i}{n+1}$	$\sqrt{1-{\left(\frac{i}{n+1}\right)}^{2}}$	*A*_*i*_+*U*_*i*_
{*GorU*}	$\frac{i}{n+1}$	$-\sqrt{1-{\left(\frac{i}{n+1}\right)}^{2}}$	*G*_*i*_+*U*_*i*_	{*CorU*}	$\frac{i}{n+1}$	$-\sqrt{1-{\left(\frac{i}{n+1}\right)}^{2}}$	*C*_*i*_+*U*_*i*_	{*CorG*}	$\frac{i}{n+1}$	$-\sqrt{1-{\left(\frac{i}{n+1}\right)}^{2}}$	*C*_*i*_+*G*_*i*_
{*A′orC′*}	$\frac{-i}{n+1}$	$\sqrt{1-{\left(\frac{i}{n+1}\right)}^{2}}$	${A^{\prime }}_{i}+{C^{\prime }}_{i}$	{*A′orG′*}	$\frac{-i}{n+1}$	$\sqrt{1-{\left(\frac{i}{n+1}\right)}^{2}}$	${A^{\prime }}_{i}+{G^{\prime }}_{i}$	{*A′orU′*}	$\frac{-i}{n+1}$	$\sqrt{1-{\left(\frac{i}{n+1}\right)}^{2}}$	${A^{\prime }}_{i}+{U^{\prime }}_{i}$
{*G′orU′*}	$\frac{-i}{n+1}$	$-\sqrt{1-{\left(\frac{i}{n+1}\right)}^{2}}$	${G^{\prime }}_{i}+\;{U^{\prime }}_{i}$	{*C′orU′*}	$\frac{-i}{n+1}$	$-\sqrt{1-{\left(\frac{i}{n+1}\right)}^{2}}$	${C^{\prime }}_{i}+\;{U^{\prime }}_{i}$	{*C′orG′*}	$\frac{-i}{n+1}$	$-\sqrt{1-{\left(\frac{i}{n+1}\right)}^{2}}$	${C^{\prime }}_{i}+\;{G^{\prime }}_{i}$

**Fig 2:**
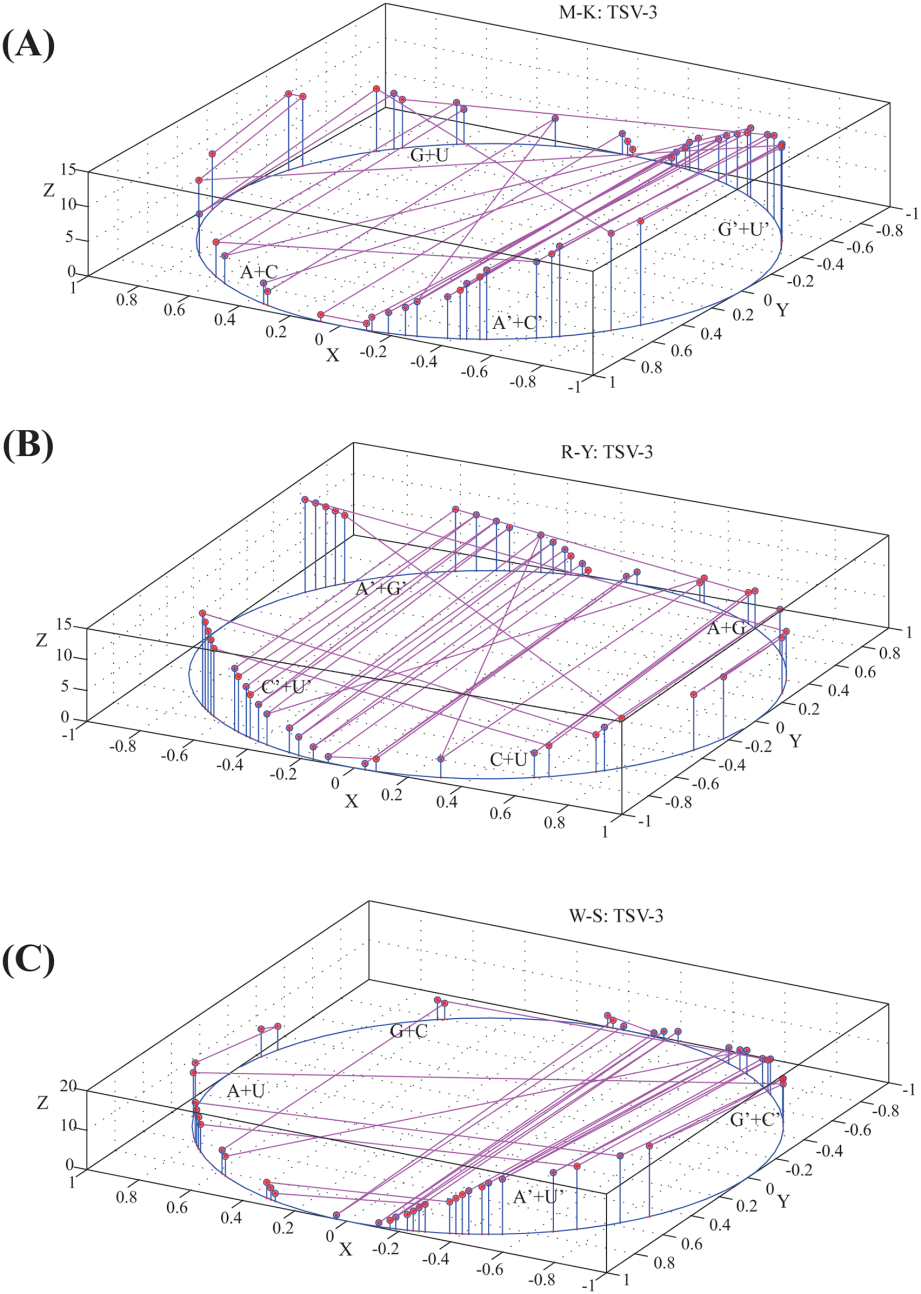
Three characteristic curves of TSV-3. (A) M-K curve, (B) R-Y curve, and (C) W-S curve. Let *G* = *g*_1_*g*_2_*g*_3_…*g*_*n*_ be the characteristic sequence of TSV-3, where *n* is the length of *G*. Each base *g*_*i*_ can be mapped into three dots *φ*_1_(*g*_*i*_) = (*x*_1*i*_, *y*_1*i*_, *z*_1*i*_), *φ*_2_(*g*_*i*_) = (*x*_2*i*_, *y*_2*i*_, *z*_2*i*_), and *φ*_3_(*g*_*i*_) = (*x*_3*i*_, *y*_3*i*_, *z*_3*i*_), where *i* = 1, 2, …, *n*. By connecting dots in *φ*_1_(*g*_*i*_) = (*x*_1*i*_, *y*_1*i*_, *z*_1*i*_) (*i* = 1, 2, …, *n*) in order, we can obtain an M-K curve to represent the characteristic sequence G. Similarly, by connecting dots in *φ*_2_(*g*_*i*_) = (*x*_2*i*_, *y*_2*i*_, *z*_2*i*_) and *φ*_3_(*g*_*i*_) = (*x*_3*i*_, *y*_3*i*_, *z*_3*i*_) respectively, we can obtain R-Y and W-S curves.

Specifically, let *G* = *g*_1_*g*_2_*g*_3_…*g*_*n*_ be the characteristic sequence of an RNA secondary structure, where *n* is the length of *G*. Each base *g*_*i*_ can be mapped into three dots *φ*_1_(*g*_*i*_) = (*x*_1*i*_, *y*_1*i*_, *z*_1*i*_), *φ*_2_(*g*_*i*_) = (*x*_2*i*_, *y*_2*i*_, *z*_2*i*_), and *φ*_3_(*g*_*i*_) = (*x*_3*i*_, *y*_3*i*_, *z*_3*i*_) where *i* = 1, 2, …, *n*. By connecting dots in *φ*_1_(*g*_*i*_) = (*x*_1*i*_, *y*_1*i*_, *z*_1*i*_) (*i* = 1, 2, …, *n*) in order, we can obtain an M-K curve to represent the characteristic sequence *G*. Similarly, by connecting dots in *φ*_2_(*g*_*i*_) (*i* = 1, 2, …, *n*) and *φ*_3_(*g*_*i*_) (*i* = 1, 2, …, *n*) respectively, we can obtain R-Y and W-S curves. Taking the secondary structure of TSV-3 as an example, we plotted its M-K, R-Y, and W-S curves in Fig 2. Obviously, the x, y, and z coordinates for each of the three curves are all dynamic. The points projected onto the X-Y plane in our method are moving around the unit circle by a certain length and the z coordinate is changed by the content of bases.

### Properties of the method

In this section, we introduced 3 properties showing 3 theoretical advantages of our method including non-degenerative (no information loss), easily reflecting the content of stem and loop, and the distribution of base frequencies. The proof of the properties were provided in S1 Text.

#### Property 1

The mapping between an RNA secondary structure and its dynamic 3D graphical representation is one to one, and the mapping on the X-Y plane is non-degenerative. Thus no information is lost.

#### Property 2

Our dynamic 3D graphical representation can easily reflect the content of bases and proportion of stem and loop structures.

This property shows that a few information on the base distribution and compositions of RNA secondary structure, such as the proportion of stem and loop structures, can be intuitively reflected by the dynamic 3D graphical representation. It is of note that the proportion of stem and loop structures is extremely important for RNA secondary structure prediction [33].

#### Property 3

For a characteristic sequence of the RNA secondary structure, let the frequencies of bases *A*′, *G*′, *C*′, *U*′, *A*, *G*, *C*, *U* be *a*′, *g*′, *c*′, *u*′, *a*, *g*, *c*, *u*, and $z_{1}^{1}=\frac{1}{n}\sum_{i=1}^{n}z_{1i}^{A,C}$, $z_{1}^{2}=\frac{1}{n}\sum_{i=1}^{n}z_{1i}^{G,U}$, $z_{1}^{3}=\frac{1}{n}\sum_{i=1}^{n}z_{1i}^{A^{\prime },C^{\prime }}$, $z_{1}^{4}=\frac{1}{n}\sum_{i=1}^{n}z_{1i}^{G^{\prime },U^{\prime }}$, $z_{2}^{1}=\frac{1}{n}\sum_{i=1}^{n}z_{2i}^{A,G}$, $z_{2}^{2}=\frac{1}{n}\sum_{i=1}^{n}z_{2i}^{C,U}$, $z_{2}^{3}=\frac{1}{n}\sum_{i=1}^{n}z_{2i}^{A^{\prime },G^{\prime }}$, $z_{2}^{4}=\frac{1}{n}\sum_{i=1}^{n}z_{2i}^{C^{\prime },U^{\prime }}$, $z_{3}^{1}=\frac{1}{n}\sum_{i=1}^{n}z_{3i}^{A,U}$, $z_{3}^{2}=\frac{1}{n}\sum_{i=1}^{n}z_{3i}^{G,C}$, $z_{3}^{3}=\frac{1}{n}\sum_{i=1}^{n}z_{3i}^{A^{\prime },U^{\prime }}$, $z_{3}^{4}=\frac{1}{n}\sum_{i=1}^{n}z_{3i}^{G^{\prime },C^{\prime }}$, *i* = 1, 2, …, *n*, where *n* is the length of the characteristic sequence, then $z_{1}^{1}$, $z_{1}^{2}$, $z_{1}^{3}$, $z_{1}^{4}$, $z_{2}^{1}$, $z_{2}^{2}$, $z_{2}^{3}$, $z_{2}^{4}$, $z_{3}^{1}$, $z_{3}^{2}$, $z_{3}^{3}$, and $z_{3}^{4}$ indicate the distribution of base frequencies in the whole sequence.

### Characterizing RNA secondary structures by 36-D vectors

For an RNA secondary structure, we have three sets of points (*x*_1*i*_, *y*_1*i*_, *z*_1*i*_), (*x*_2*i*_, *y*_2*i*_, *z*_2*i*_), and (*x*_3*i*_, *y*_3*i*_, *z*_3*i*_), *i* = 1, 2, …, *n*, where *n* is the length of the structure. We draw the geometrical center of the three curves to construct a 36-dimensional vector denoted by $[x_{1}^{1},y_{1}^{1},z_{1}^{1},x_{1}^{2},y_{1}^{2},z_{1}^{2},x_{1}^{3},y_{1}^{3},z_{1}^{3},x_{1}^{4},y_{1}^{4},z_{1}^{4},x_{2}^{1},y_{2}^{1},z_{2}^{1},x_{2}^{2},y_{2}^{2},z_{2}^{2},x_{2}^{3},y_{2}^{3},z_{2}^{3},x_{2}^{4},y_{2}^{4},z_{2}^{4},x_{3}^{1},y_{3}^{1},z_{3}^{1},$ $x_{3}^{2},y_{3}^{2},z_{3}^{2},x_{3}^{3},y_{3}^{3},z_{3}^{3},x_{3}^{4},y_{3}^{4},z_{3}^{4}]$ in which, $x_{1}^{1}=\frac{1}{n}\sum_{i=1}^{n}x_{1i}^{A,C}$, $y_{1}^{1}=\frac{1}{n}\sum_{i=1}^{n}y_{1i}^{A,C}$, $z_{1}^{1}=\frac{1}{n}\sum_{i=1}^{n}z_{1i}^{A,C}$, $x_{1}^{2}=\frac{1}{n}\sum_{i=1}^{n}x_{1i}^{G,U}$, $y_{1}^{2}=\frac{1}{n}\sum_{i=1}^{n}y_{1i}^{G,U}$, $z_{1}^{2}=\frac{1}{n}\sum_{i=1}^{n}z_{1i}^{G,U}$, $x_{1}^{3}=\frac{1}{n}\sum_{i=1}^{n}x_{1i}^{A^{\prime },C^{\prime }}$, $y_{1}^{3}=\frac{1}{n}\sum_{i=1}^{n}y_{1i}^{A^{\prime },C^{\prime }}$, $z_{1}^{3}=\frac{1}{n}\sum_{i=1}^{n}z_{1i}^{A^{\prime },C^{\prime }}$, $x_{1}^{4}=\frac{1}{n}\sum_{i=1}^{n}x_{1i}^{G^{\prime },U^{\prime }}$, $y_{1}^{4}=\frac{1}{n}\sum_{i=1}^{n}y_{1i}^{G^{\prime },U^{\prime }}$, $z_{1}^{4}=\frac{1}{n}\sum_{i=1}^{n}z_{1i}^{G^{\prime },U^{\prime }}$, $x_{2}^{1}=\frac{1}{n}\sum_{i=1}^{n}x_{2i}^{A,G}$, $y_{2}^{1}=\frac{1}{n}\sum_{i=1}^{n}y_{2i}^{A,G}$, $z_{2}^{1}=\frac{1}{n}\sum_{i=1}^{n}z_{2i}^{A,G}$, $x_{2}^{2}=\frac{1}{n}\sum_{i=1}^{n}x_{2i}^{C,U}$, $y_{2}^{2}=\frac{1}{n}\sum_{i=1}^{n}y_{2i}^{C,U}$, $z_{2}^{2}=\frac{1}{n}\sum_{i=1}^{n}z_{2i}^{C,U}$, $x_{2}^{3}=\frac{1}{n}\sum_{i=1}^{n}x_{2i}^{A^{\prime },G^{\prime }}$, $y_{2}^{3}=\frac{1}{n}\sum_{i=1}^{n}y_{2i}^{A^{\prime },G^{\prime }}$, $z_{2}^{3}=\frac{1}{n}\sum_{i=1}^{n}z_{2i}^{A^{\prime },G^{\prime }}$, $x_{2}^{4}=\frac{1}{n}\sum_{i=1}^{n}x_{2i}^{C^{\prime },U^{\prime }}$, $y_{2}^{4}=\frac{1}{n}\sum_{i=1}^{n}y_{2i}^{C^{\prime },U^{\prime }}$, $z_{2}^{4}=\frac{1}{n}\sum_{i=1}^{n}z_{2i}^{C^{\prime },U^{\prime }}$, $x_{3}^{1}=\frac{1}{n}\sum_{i=1}^{n}x_{3i}^{A,U}$, $y_{3}^{1}=\frac{1}{n}\sum_{i=1}^{n}y_{3i}^{A,U}$, $z_{3}^{1}=\frac{1}{n}\sum_{i=1}^{n}z_{3i}^{A,U}$, $x_{3}^{2}=\frac{1}{n}\sum_{i=1}^{n}x_{3i}^{G,C}$, $y_{3}^{2}=\frac{1}{n}\sum_{i=1}^{n}y_{3i}^{G,C}$, $z_{3}^{2}=\frac{1}{n}\sum_{i=1}^{n}z_{3i}^{G,C}$, $x_{3}^{3}=\frac{1}{n}\sum_{i=1}^{n}x_{3i}^{A^{\prime },U^{\prime }}$, $y_{3}^{3}=\frac{1}{n}\sum_{i=1}^{n}y_{3i}^{A^{\prime },U^{\prime }}$, $z_{3}^{3}=\frac{1}{n}\sum_{i=1}^{n}z_{3i}^{A^{\prime },U^{\prime }}$, $x_{3}^{4}=\frac{1}{n}\sum_{i=1}^{n}x_{3i}^{G^{\prime },C^{\prime }}$, $y_{3}^{4}=\frac{1}{n}\sum_{i=1}^{n}y_{3i}^{G^{\prime },C^{\prime }}$, $z_{3}^{4}=\frac{1}{n}\sum_{i=1}^{n}z_{3i}^{G^{\prime },C^{\prime }}$.

The 36-dimensional vector was adopted as a descriptor to characterize RNA secondary structures. It is of note that according to the 3 properties in the previous section and the compactness and uniqueness of the three curves for a given RNA secondary structure, this dynamic graphical representation scheme can reflect the distribution of bases in the characteristic sequence and has small degeneracy. Thus, we adopted it to compute the similarity between RNA secondary structures. Specifically, for any two RNA secondary structures, we first constructed their 36D representative vectors, and then calculated the similarity between the two vectors by the quotient between (1) the Euclidean distance between their end-points (in graph) and (2) the cosine of the angle between the two vectors. Clearly, the smaller is the quotient, the more similar are the two RNA secondary structures.

## Results and Discussion

### Similarities between nine RNA secondary structures of virus

We drew in Fig 1 the RNA secondary structures for nine viruses in **Dataset I**, and listed their pairwise similarities in Table 2.

**Table 2:** The similarity/dissimilarity matrix for nine RNA secondary structures in Fig 1 based on RNA 36D vector representation.

**species**	**AlMV-3**	**APMV-3**	**AVII**	**CiLRV-3**	**CVV-3**	**EMV-3**	**LRMV-3**	**PDV-3**	**TSV-3**
**AlMV-3**	0	1.5387	4.4078	5.2166	2.6300	4.7192	3.9887	1.7040	4.4352
**APMV-3**		0	5.4632	7.0070	2.9442	6.2642	5.0473	1.5851	5.6373
**AVII**			0	2.0993	2.6788	1.0170	0.9822	4.1554	2.1266
**CiLRV-3**				0	4.6843	1.6580	1.9534	6.0340	1.2103
**CVV-3**					0	3.4462	2.8271	1.9629	4.1915
**EMV-3**						0	1.0041	4.7944	2.0004
**LRMV-3**							0	3.7523	1.7026
**PDV-3**								0	4.8894
**TSV-3**									0

**Fig 3:**
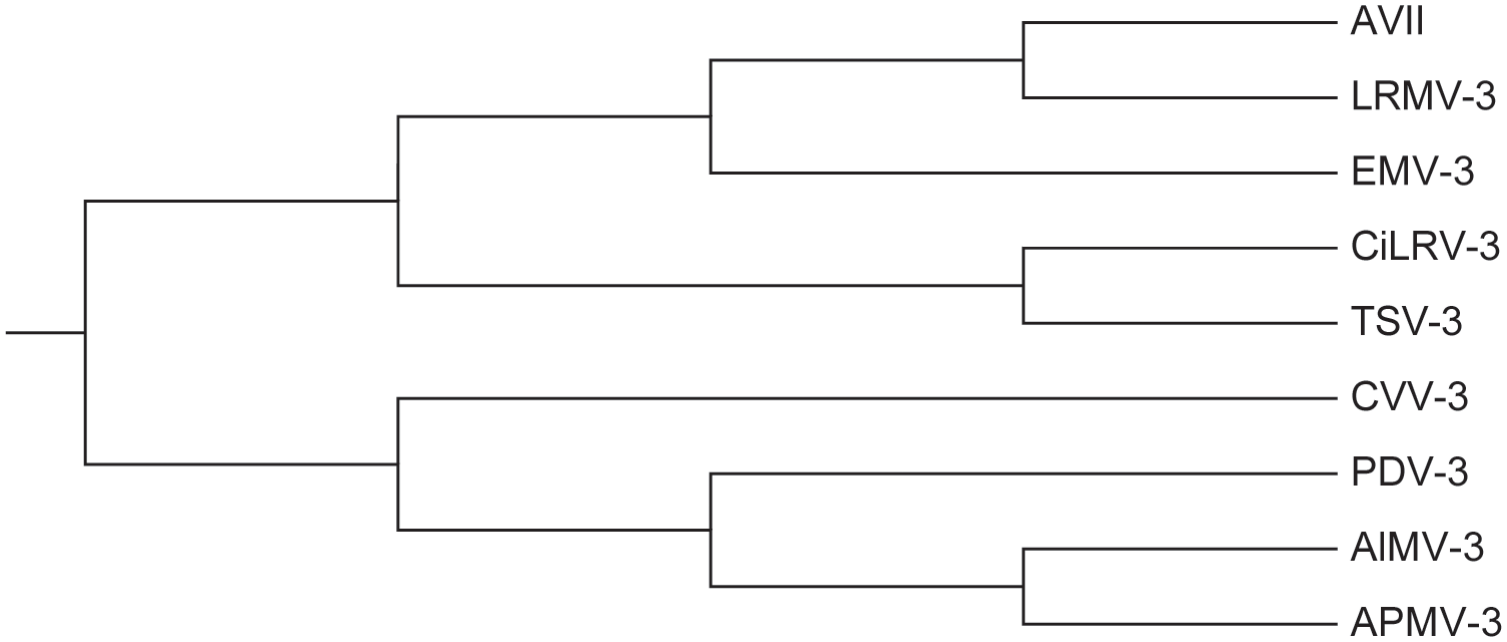
The phylogenetic tree for nine virus in Fig 1. The tree was constructed using UPGMA, in which the distance matrix is calculated by our RNA comparison method based on 36D vector representation.

As Table 2 shows, the smallest entries are associated with pairs (AVII, LRMV-3), (LRMV-3, EMV-3), and (AVII, EMV-3), which indicates that AVII, LRMV-3, and EMV-3 are more similar to each other. On the other hand, APMV-3, AlMV-3, and PDV-3 show great dissimilarity with others. This is consistent with the results reported by Liao et al. [34, 35], Yao et al. [22], Li et al. [24], and Bai et al. [36]. For a better view of our results, we constructed a phylogenetic tree (see Fig 3) for the nine RNA structures based on the 9×9 similarity/dissimilarity matrix using UPGMA method in MEGA5.1. The results indicate that the 36D vectors can catch some intrinsic characteristics of RNA secondary structures.

### Further test on Dataset II and III

To further evaluate the sensitivity of this algorithm in measuring the similarities among complex RNA secondary structures, we applied it to **Dataset II**, whose characteristic sequences were shown in S1 Table.

Based on [37, 38], the 17 complex RNA secondary structures shown in S1(A) Fig of RNase P database can be divided into six groups: (1) Gamma Purple Bacteria RNase P structures (Klebsiella pneumoniae, Serratia marcescens, Escherichia coli and Chromatium vinosum), (2) Green sulfur Bacteria RNase P structures (Chlorobium limicola and Chlorobium tepidum), (3) Low G+C Gram positive RNase P structures (Bacillus subtilis and Enterococcus faecalis), (4) Cyanobacterial RNase P structures (Calothrix PCC7601, Anabaena PCC7120 and Synechocystis PCC6803), (5) Archaea Euryarchaeal RNase P structures (Thermococcus celer, Pyrococcus horikoshii and T. Litoralis), and (6) Nuclear RNase P structures (Pan troglodytes, Macaca mulatta, Pongo pygmaeus). In addition, according to pseudo-knot structures, the 16 pseudo-knot secondary structures shown in S1(B) Fig can be divided into five groups: (PKB44, PKB46, PKB4, PKB42, PKB43), (PKB94, PKB114, PKB84), (PKB131, PKB132), (PKB134, PKB135), and (PKB144, PKB140, PKB142, PKB143).

**Fig 4:**
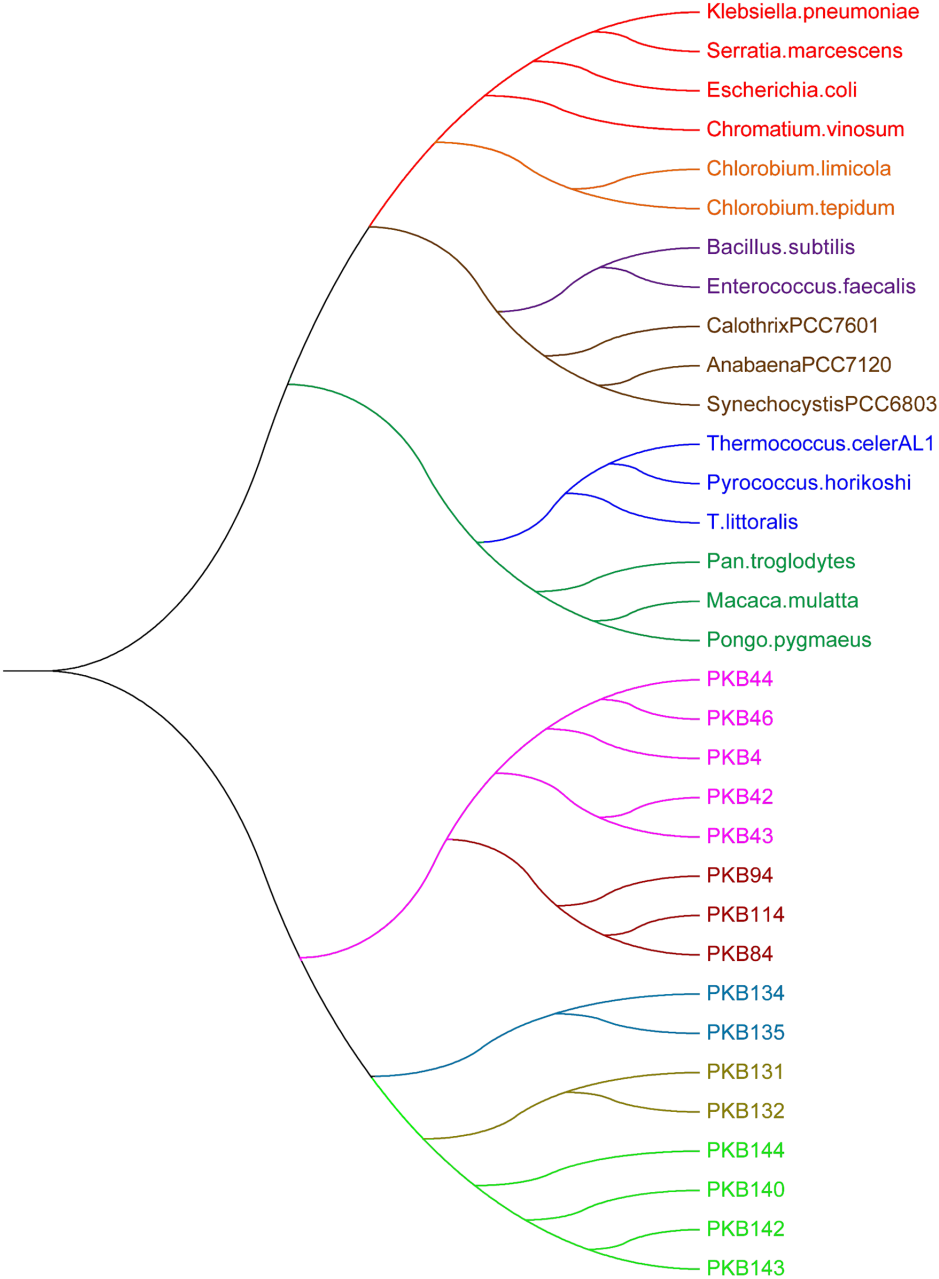
The phylogenetic tree for RNA secondary structures in S1 Fig. Branch colors indicate six clusters of RNase P structures from RNase P database and five clusters of pseudo-knot structures from Pseud Base. The six clusters of RNase P structures include: (1) Gamma Purple Bacteria RNase P structures, (2) Green sulfur Bacteria RNase P structures, (3) Low G+C Gram positive RNase P structures, (4) Cyanobacterial RNase P structures, (5) Archaea Euryarchaeal RNase P structures, and (6) Nuclear RNase P structures.

We plotted the similarities among the 33 RNA secondary structures (based on their 36D vector representations) in Fig 4. There are 11 branches corresponding to the six classes of RNase P structures and five classes of pseudo-knot structures respectively. Thus, our method perfectly separated the RNA classes in **Dataset II**.

**Fig 5:**
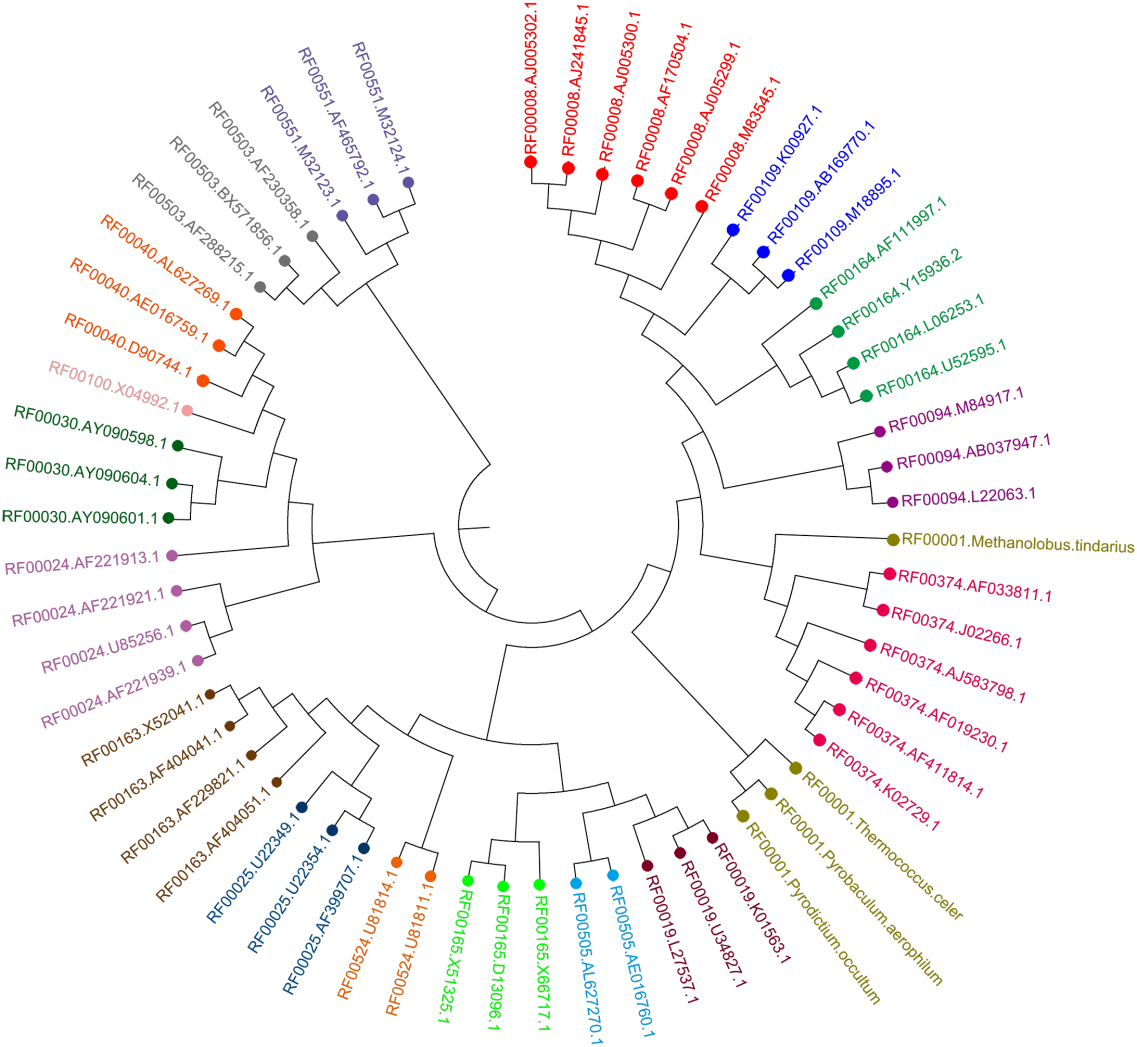
The phylogenetic tree for non-coding RNA secondary structures in S2 Fig. The 18 branch colors represent 18 non-coding RNA families respectively from RNAstrand database, including 5S rRNA (RF00001), Gammaretro_CES (RF00374), Hepatitis delta virus ribozyme (RF00094), Vimentin3 (RF00109), Corona_pk3 (RF00165), Y_RNA (RF00019), s2m (RF00164), Hammerhead ribozyme (type III) (RF00008), Ciliate telomerase RNA (RF00025), R2 RNA element (RF00524), Hammerhead ribozyme (type I) (RF00163), Vertebrate telomerase RNA (RF00024), rne5 (RF00040), RNase MRP (RF00030), 7SK RNA (RF00100), RNAIII (RF00503), RydC RNA (RF00505), and Bicoid 3 prime-UTR regulatory element (RF00551).

By a similar process, we constructed a phylogenetic tree (Fig 5) for the secondary structures of 60 ncRNAs in **Dataset III**, whose characteristic sequences were shown in S2 Table. The phylogenetic tree presents clearly 18 branches corresponding to the 18 non-coding RNA families with only one mis-clustering, i.e., RF00001.Methanolobus.tindarius was classified into the cluster RF00374. The results show that our approach performs well in comparing the secondary structures of non-coding RNAs.

### Comparison with other methods on Dataset II and III

We compared our similarity measure with other nine popular RNA comparison methods, including a similarity metric based on the wavelet decomposition of the TV-Curve of ncRNA [27], LZ complexity by Liu et al. [39], five 3D graphical representations of RNA secondary structures proposed by Liao et al. [34], Zhu et al. [25], Feng et al. [40], Liu et al. [41], and Luo et al. [42], and two different 2D graphical representations of RNA secondary structures provided by Li et al. [24] and Yao et al. [43] respectively. We applied the nine methods into **Dataset II** and **III**, and the RNA similarities measured by these methods were shown as UPGMA trees in S3–S20 Figs.

As can be seen, the method based on wavelet decomposition [27] failed in separating RNase P from others (see S3 Fig) and mis-classified RF00024.AF221913.1 and RF00001.Thermococcus.celer (see S4 Fig). Similarly, as shown in S5–S18 Figs, the five 3D and two 2D graphical representations performed weakly in comparing 33 RNA secondary structures in **Dataset II**. In addition, except for the method provided by Luo et al. [42], the other six methods also presented weak classifications for the 18 non-coding RNA families in **Dataset III**. Specifically, the method by Liu et al. [39] cannot distinguish RNAs with pseudo-knots from Pseud Base in **Dataset II** (see S19 and S20 Figs). An easy comparison in S3 Table showed that our method outperformed the above nine approaches. Our 36D geometrical center vector may capture some intrinsic characteristics of the non-coding RNA and pseudo-knot structures. Moreover, the points projected onto the X-Y plane in our method are dynamic. This is different from Liao [34] and Zhu [25], in which they fixed x and y coordinates. These dynamic x and y coordinates may provide more “order” information along the secondary structure than the fixed ones.

## Conclusion

Based on the chemical property and order of bases, we first proposed a dynamic 3D graphical visualization scheme for RNA secondary structures. We then extracted digital features of the dynamic 3D graphs to compute the similarity between two RNA secondary structures. The method was applied to ncRNAs from the Rfam database and achieved good classification results. In the end, we compared our method with other nine popular approaches and showed that our method outperformed them on RNAs with pseudo-knot and non-coding RNAs. In the future, it will be interesting to extend this method to capture more features of RNA secondary structure, and interpret the information carried by the graphical representation. The additional features and information will be useful in developing a more efficient method to measure RNA structure similarity.

## Supporting Information

S1 Fig 33 RNA secondary structures in Dataset II. (A) 17 complicated RNA secondary structures from RNase P database: Klebsiella pneumoniae, Serratia marcescens, Escherichia coli K-12 W3110, Chromatium vinosum, Chlorobium limicola thiosulfatophilum, Chlorobium tepidum, Bacillus subtilis 168, Enterococcus (ex-Streptococcus) faecalis, Calothrix PCC7601, Anabaena PCC7120, Synechocystis PCC6803, Thermococcus celer AL-1, Pyrococcus horikoshii strain OT3, T. Litoralis, Pan troglodytes, Macaca mulatta, Pongo pygmaeus. (B) 16 RNA secondary structures with pseudo-knots from Pseud Base: PKB44, PKB46, PKB4, PKB42, PKB43, PKB94, PKB114, PKB84, PKB134, PKB135, PKB131, PKB132, PKB144, PKB140, PKB142, PKB143.
(DOC)

S2 Fig 18 kinds of non-coding RNA secondary structures from RNAstrand database. (A) 5S rRNA (downloaded from Gutell Lab CRW Site in RNAstrand database and belonging to the RF00001 family of Rfam database). (B) Gammaretro_CES (RF00374). (C) Hepatitis delta virus ribozyme (RF00094). (D) Vimentin3 (RF00109). (E) Corona_pk3 (RF00165). (F) Y_RNA (RF00019). (G) s2m (RF00164). (H) Hammerhead ribozyme (type III) (RF00008). (I) Ciliate telomerase RNA (RF00025). (J) R2 RNA element (RF00524). (K) Hammerhead ribozyme (type I) (RF00163). (L) Vertebrate telomerase RNA (RF00024). (M) rne5 (RF00040). (N) RNase MRP (RF00030). (O) 7SK RNA (RF00100). (P) RNAIII (RF00503). (Q) RydC RNA (RF00505). (R) Bicoid 3 prime-UTR regulatory element (RF00551) (non-coding RNA secondary structures belonging to B-R are obtained from Rfam database in RNAstrand database).
(DOC)

S3 Fig The Phylogenetic tree by multi-scale RNA comparison based on RNA triple vector curve representation for the secondary structures of RNAs in S1 Fig. (DOC)

S4 Fig The Phylogenetic tree by multi-scale RNA comparison based on RNA triple vector curve representation for the secondary structures of RNAs in S2 Fig. (DOC)

S5 Fig The two phylogenetic trees for the secondary structures of RNAs in S1 Fig based on the method by Liao *et al* [34]. (A) The phylogenetic tree based on the Euclidean distance. (B) The phylogenetic tree based on the Angle.
(DOC)

S6 Fig The two phylogenetic trees for the secondary structures of RNAs in S2 Fig based on the method by Liao *et al* [34]. (A) The phylogenetic tree based on the Euclidean. (B) The phylogenetic tree based on the Angle.
(DOC)

S7 Fig The two phylogenetic trees for the secondary structures of RNAs in S1 Fig based on the method by Zhu *et al* [25]. (A) The phylogenetic tree based on the Euclidean distance. (B) The phylogenetic tree based on the Angle.
(DOC)

S8 Fig The two phylogenetic trees for the secondary structures of RNAs in S2 Fig based on the method by Zhu *et al* [25]. (A) The phylogenetic tree based on the Euclidean distance. (B) The phylogenetic tree based on the Angle.
(DOC)

S9 Fig The phylogenetic tree for the secondary structures of RNAs in S1 Fig based on the method by Feng *et al* [40]. (DOC)

S10 Fig The phylogenetic tree for the secondary structures of RNAs in S2 Fig based on the method by Feng *et al* [40]. (DOC)

S11 Fig The two phylogenetic trees for the secondary structures of RNAs in S1 Fig based on the method by Liu *et al* [41]. (A) The phylogenetic tree based on the Euclidean distance. (B) The phylogenetic tree based on the Angle.
(DOC)

S12 Fig The two phylogenetic trees for the secondary structures of RNAs in S2 Fig based on the method by Liu *et al* [41]. (A) The phylogenetic tree based on the Euclidean distance. (B) The phylogenetic tree based on the Angle.
(DOC)

S13 Fig The phylogenetic tree for the secondary structures of RNAs in S1 Fig based on the method by Luo *et al* [42]. (DOC)

S14 Fig The phylogenetic tree for the secondary structures of RNAs in S2 Fig based on the method by Luo *et al* [42]. (DOC)

S15 Fig The phylogenetic tree for the secondary structures of RNAs in S1 Fig based on the method by Li *et al* [24]. (DOC)

S16 Fig The phylogenetic tree for the secondary structures of RNAs in S2 Fig based on the method by Li *et al* [24]. (DOC)

S17 Fig The two phylogenetic trees for the secondary structures of RNAs in S1 Fig based on the method by Yao *et al* [43]. (A) The phylogenetic tree based on the Euclidean distance. (B) The phylogenetic tree based on the Angle.
(DOC)

S18 Fig The two phylogenetic trees for the secondary structures of RNAs in S2 Fig based on the method by Yao *et al* [43]. (A) The phylogenetic tree based on the Euclidean distance. (B) The phylogenetic tree based on the Angle.
(DOC)

S19 Fig The three phylogenetic trees for the secondary structures of RNAs in S1 Fig based on the method by Liu *et al* [39]. (A) The phylogenetic tree based on non-*A*(*A*′) sequences. (B) The phylogenetic tree based on the non-*C*(*C*′) sequences. (C) The phylogenetic tree based on the non-*G*(*G*′) sequences.
(DOC)

S20 Fig The three phylogenetic trees for the secondary structures of non-coding RNAs in S2 Fig based on the method by Liu *et al* [39]. (A) The phylogenetic tree based on the non-*A*(*A*′) sequences. (B) The phylogenetic tree based on the non-*C*(*C*′) sequences. (C) The phylogenetic tree based on the non-*G*(*G*′) sequences.
(DOC)

S1 Software RnaFeatureGenerator. (ZIP)

S1 Table The characteristic sequences for the secondary structures of RNAs in S1 Fig (A, G, C and U located in the base pairs are denoted as a, g, c and u). (DOC)

S2 Table The characteristic sequences for the secondary structures of RNAs in S2 Fig (A, G, C and U located in the base pairs are denoted as a, g, c and u). (DOC)

S3 Table The comparison between our method and the other nine algorithms. (DOC)

S1 Text The proof of three properties. (DOC)
