# Supplementary material for: A Dynamic 3D Graphical Representation for RNA Structure Analysis and Its Application in Non-Coding RNA Classification
Source: PLoS One. 2016 May 23;11(5):e0152238. doi: 10.1371/journal.pone.0152238 (PMC4877074; doi:10.1371/journal.pone.0152238)
Supplement: S1 Fig — (A) 17 complicated RNA secondary structures from RNase P database: Klebsiella pneumoniae, Serratia marcescens, Escherichia coli K-12 W3110, Chromatium vinosum, Chlorobium limicola thiosulfatophilum, Chlorobium tepidum, Bacillus subtilis 168, Enterococcus (ex-Streptococcus) faecalis, Calothrix PCC7601, Anabaena PCC7120, Synechocystis PCC6803, Thermococcus celer AL-1, Pyrococcus horikoshii strain OT3, T. Litoralis, Pan troglodytes, Macaca mulatta, Pongo pygmaeus. (B) 16 RNA secondary structures with pseudo-knots from Pseud Base: PKB44, PKB46, PKB4, PKB42, PKB43, PKB94, PKB114, PKB84, PKB134, PKB135, PKB131, PKB132, PKB144, PKB140, PKB142, PKB143. (DOC) [file pone.0152238.s001.doc]

**S1 Fig. 33 RNA secondary structures in Dataset II.** (A) 17 complicated RNA secondary structures from RNase P database: Klebsiella pneumoniae, Serratia marcescens, Escherichia coli K-12 W3110, Chromatium vinosum, Chlorobium limicola thiosulfatophilum, Chlorobium tepidum, Bacillus subtilis 168, Enterococcus (ex-Streptococcus) faecalis, Calothrix PCC7601, Anabaena PCC7120, Synechocystis PCC6803, Thermococcus celer AL-1, Pyrococcus horikoshii strain OT3, T. Litoralis, Pan troglodytes, Macaca mulatta, Pongo pygmaeus. (B) 16 RNA secondary structures with pseudo-knots from Pseud Base: PKB44, PKB46, PKB4, PKB42, PKB43, PKB94, PKB114, PKB84, PKB134, PKB135, PKB131, PKB132, PKB144, PKB140, PKB142, PKB143.
