# Supplementary material for: A Dynamic 3D Graphical Representation for RNA Structure Analysis and Its Application in Non-Coding RNA Classification
Source: PLoS One. 2016 May 23;11(5):e0152238. doi: 10.1371/journal.pone.0152238 (PMC4877074; doi:10.1371/journal.pone.0152238)
Supplement: S2 Fig — (A) 5S rRNA (downloaded from Gutell Lab CRW Site in RNAstrand database and belonging to the RF00001 family of Rfam database). (B) Gammaretro_CES (RF00374). (C) Hepatitis delta virus ribozyme (RF00094). (D) Vimentin3 (RF00109). (E) Corona_pk3 (RF00165). (F) Y_RNA (RF00019). (G) s2m (RF00164). (H) Hammerhead ribozyme (type III) (RF00008). (I) Ciliate telomerase RNA (RF00025). (J) R2 RNA element (RF00524). (K) Hammerhead ribozyme (type I) (RF00163). (L) Vertebrate telomerase RNA (RF00024). (M) rne5 (RF00040). (N) RNase MRP (RF00030). (O) 7SK RNA (RF00100). (P) RNAIII (RF00503). (Q) RydC RNA (RF00505). (R) Bicoid 3 prime-UTR regulatory element (RF00551) (non-coding RNA secondary structures belonging to B-R are obtained from Rfam database in RNAstrand database). (DOC) [file pone.0152238.s002.doc]

**S2 Fig. 18 kinds of non-coding RNA secondary structures from RNAstrand database.** (A) 5S rRNA (downloaded from Gutell Lab CRW Site in RNAstrand database and belonging to the RF00001 family of Rfam database). (B) Gammaretro_CES (RF00374). (C) Hepatitis delta virus ribozyme (RF00094). (D) Vimentin3 (RF00109). (E) Corona_pk3 (RF00165). (F) Y_RNA (RF00019). (G) s2m (RF00164). (H) Hammerhead ribozyme (type III) (RF00008). (I) Ciliate telomerase RNA (RF00025). (J) R2 RNA element (RF00524). (K) Hammerhead ribozyme (type I) (RF00163). (L) Vertebrate telomerase RNA (RF00024). (M) rne5 (RF00040). (N) RNase MRP (RF00030). (O) 7SK RNA (RF00100). (P) RNAIII (RF00503). (Q) RydC RNA (RF00505). (R) Bicoid 3 prime-UTR regulatory element (RF00551) (non-coding RNA secondary structures belonging to B-R are obtained from Rfam database in RNAstrand database).

**(A)**


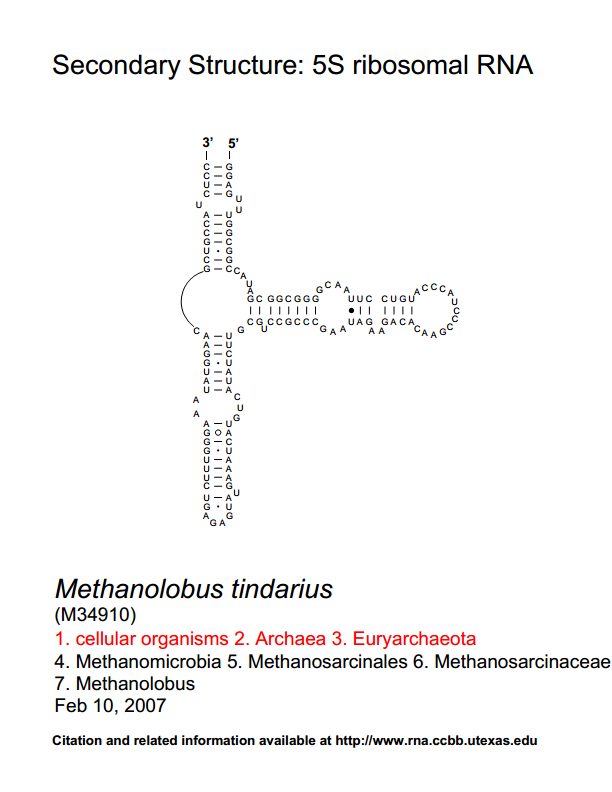

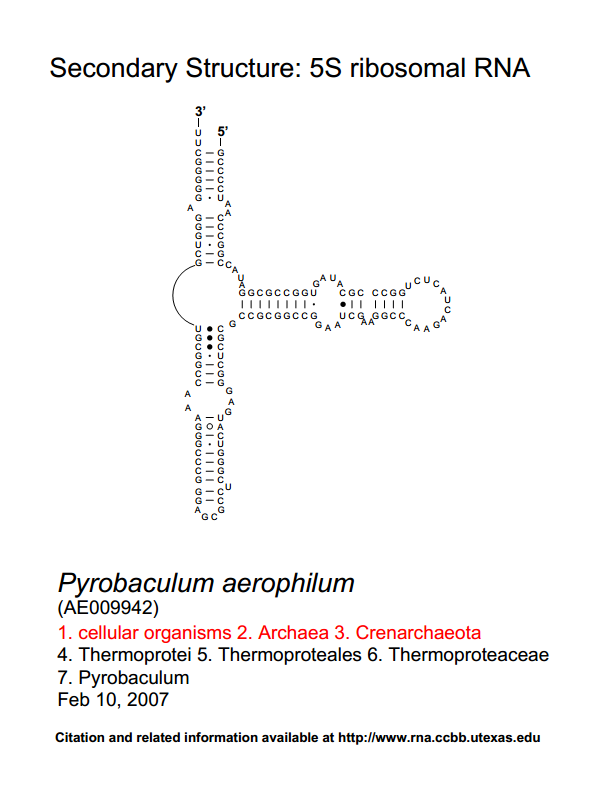

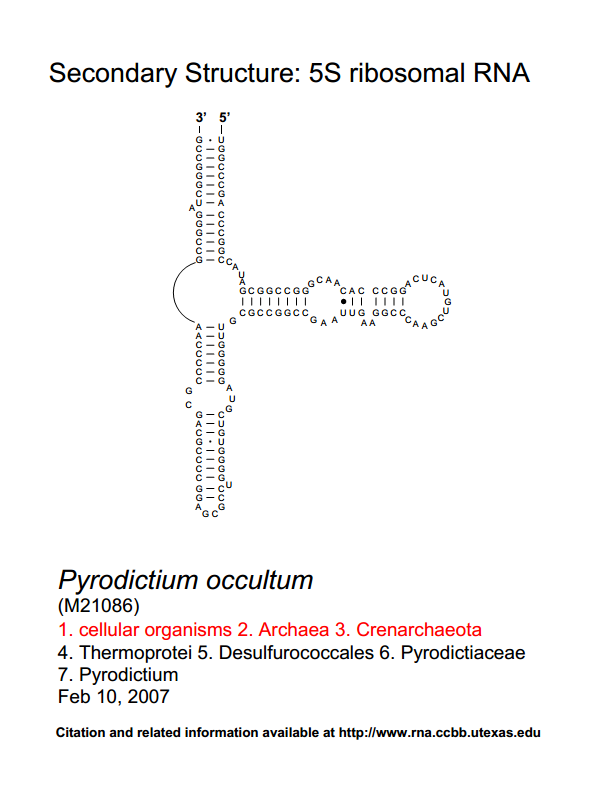

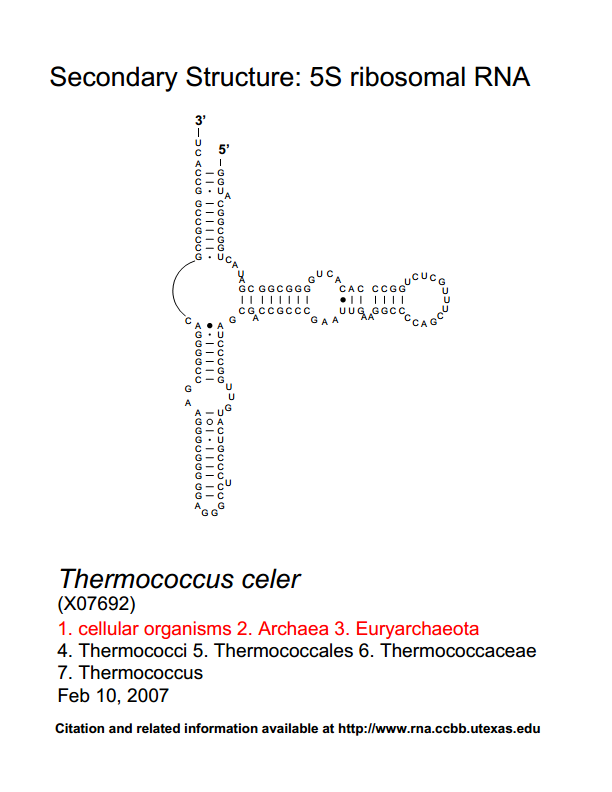


**(B)**


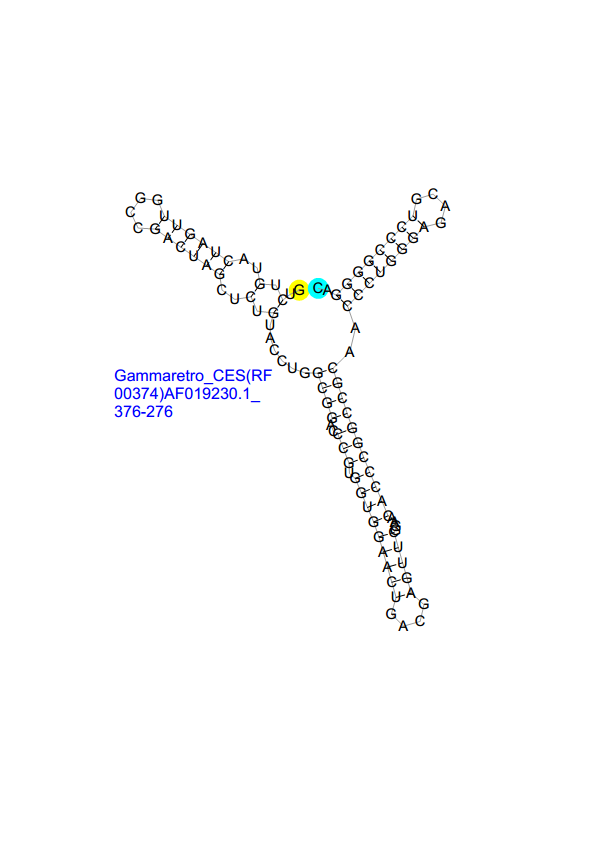

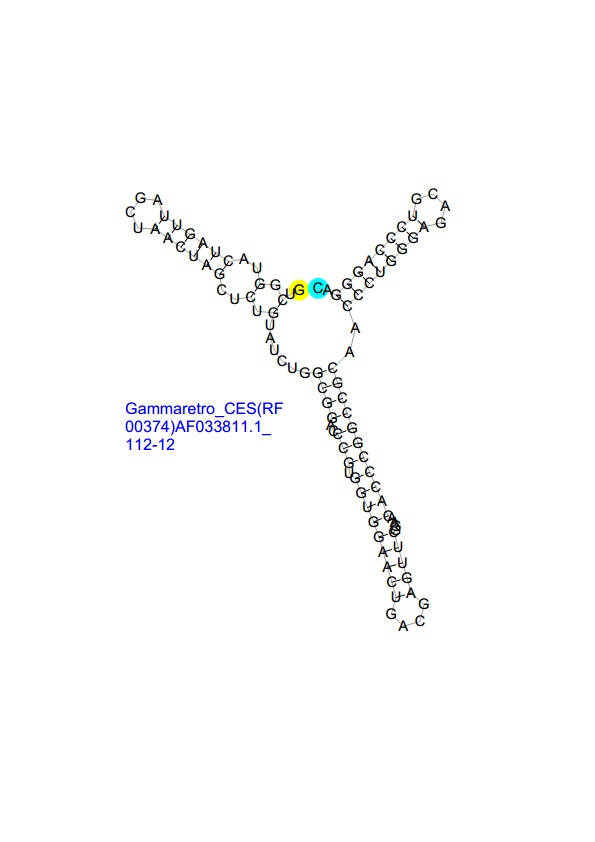

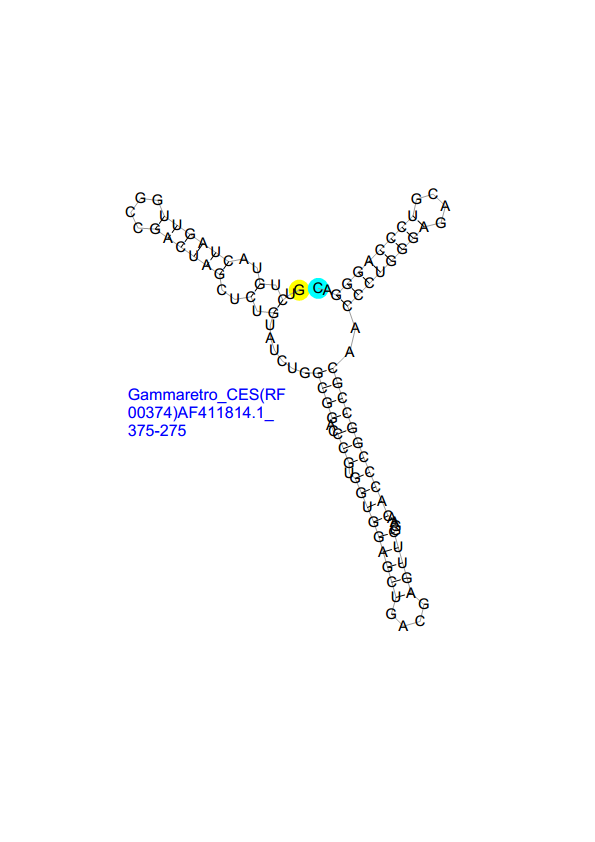

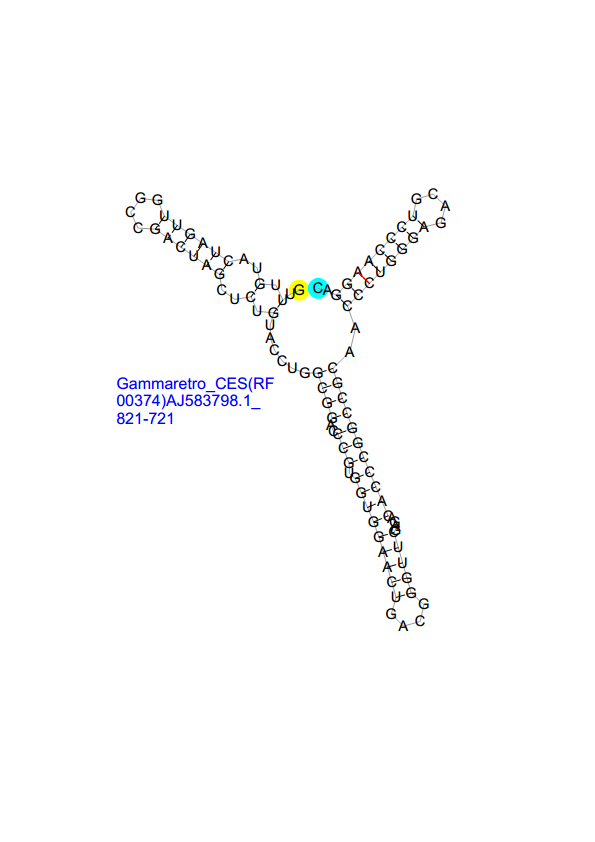

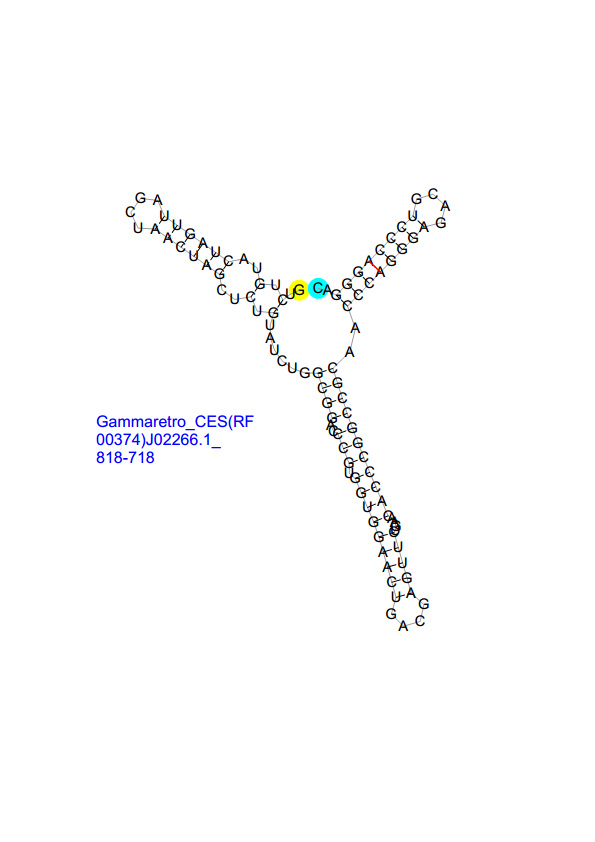

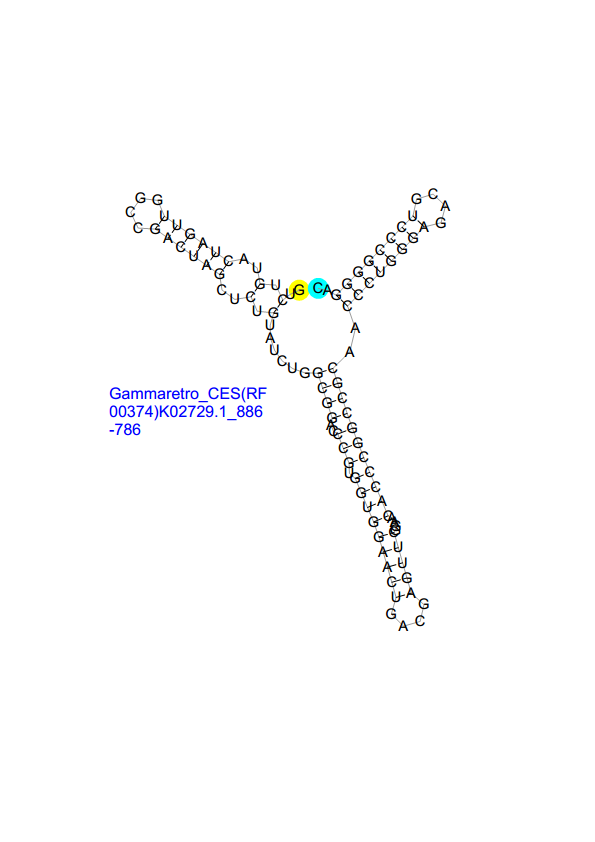


**(C)**


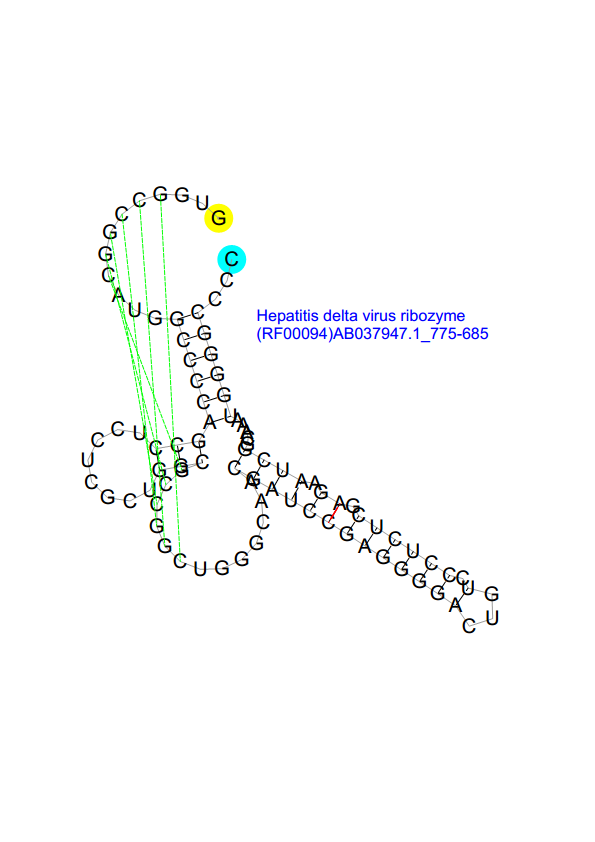

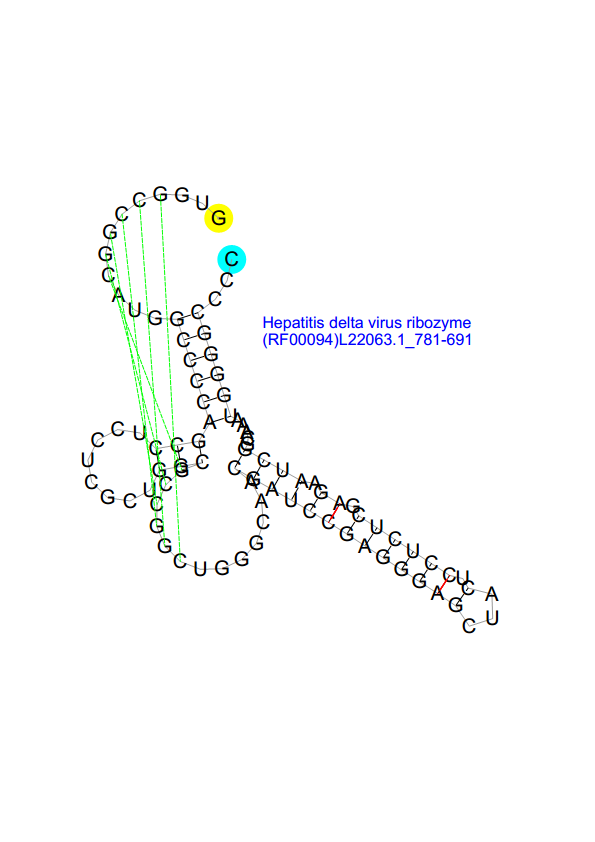

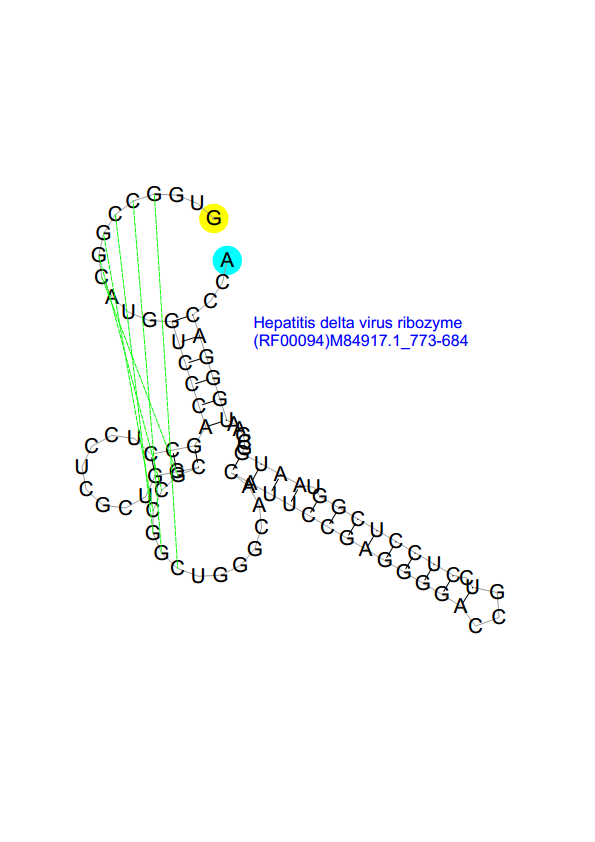


**(D)**


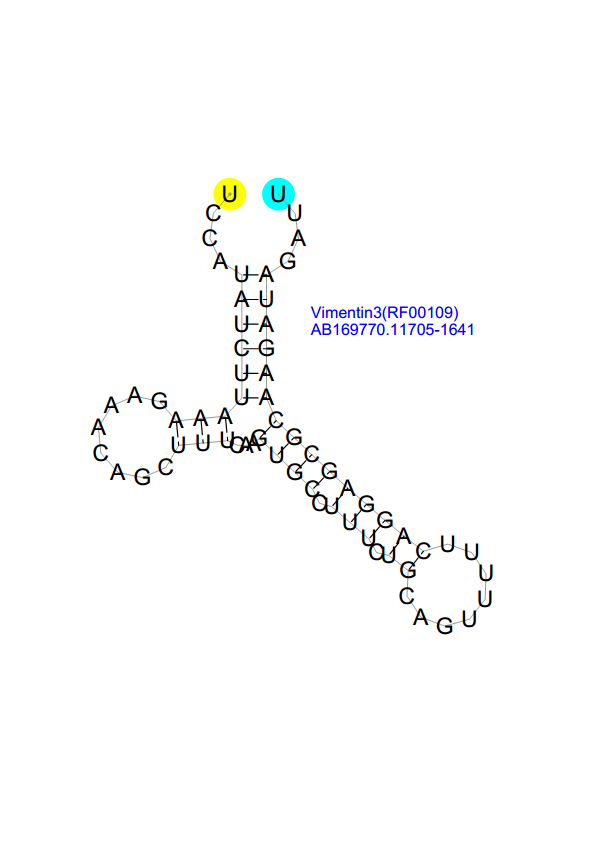

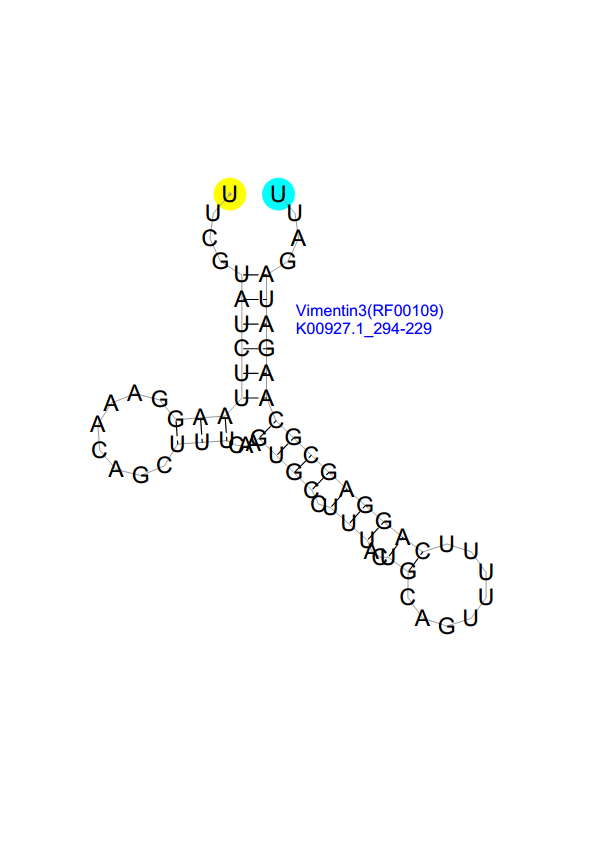

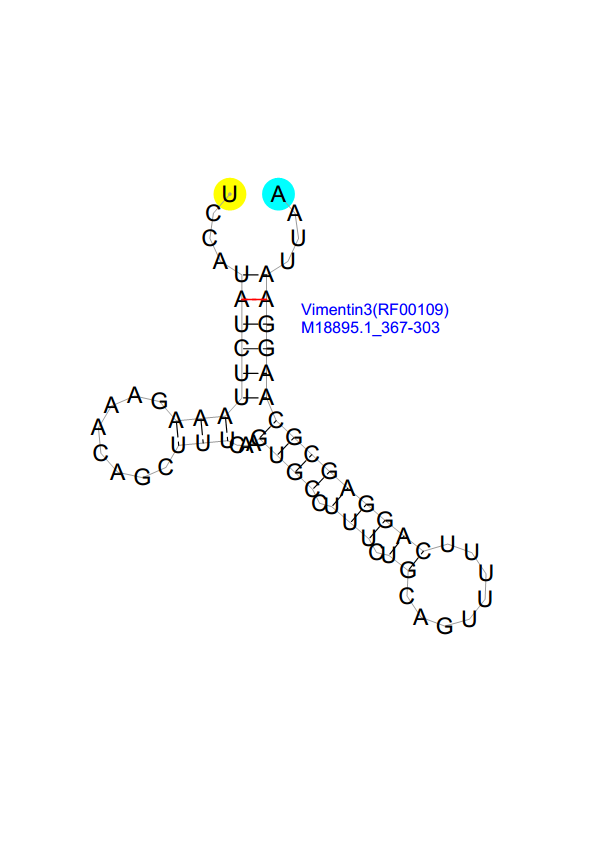


**(E)**


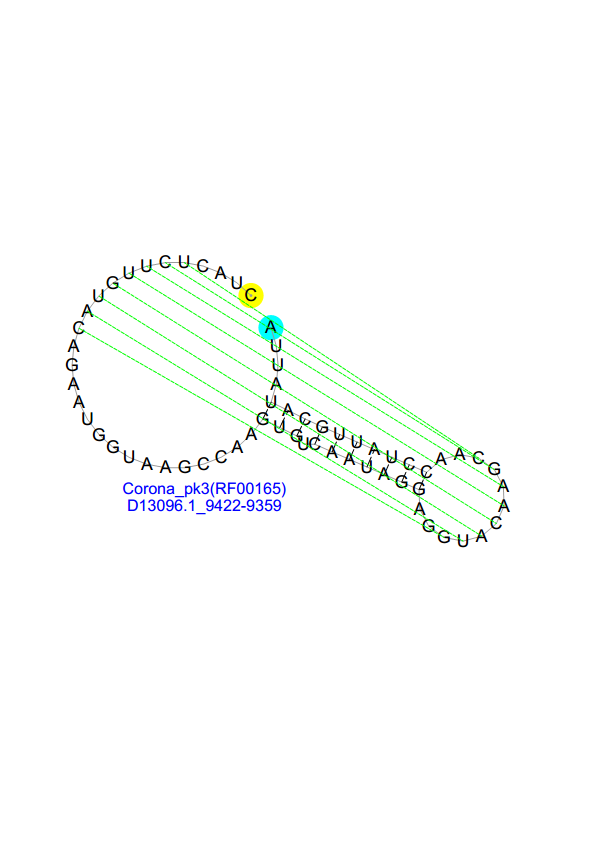

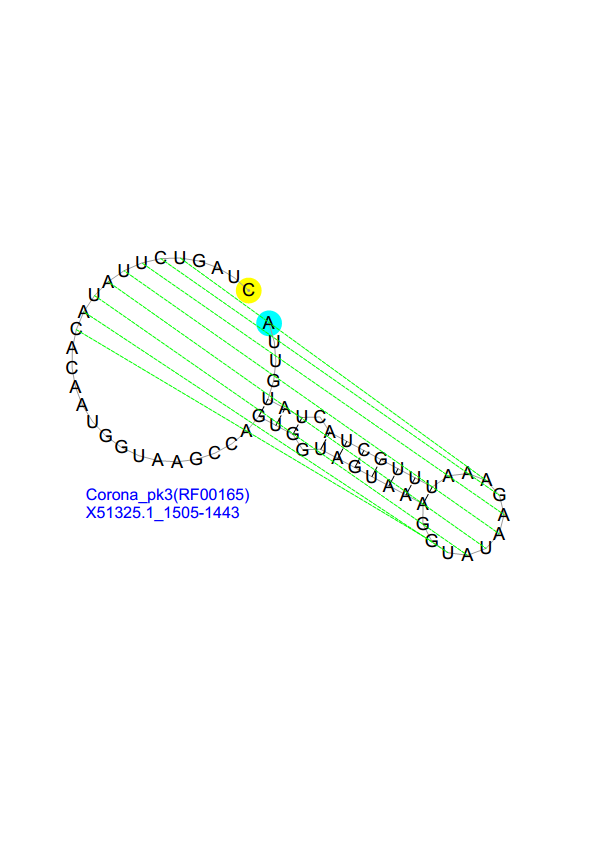

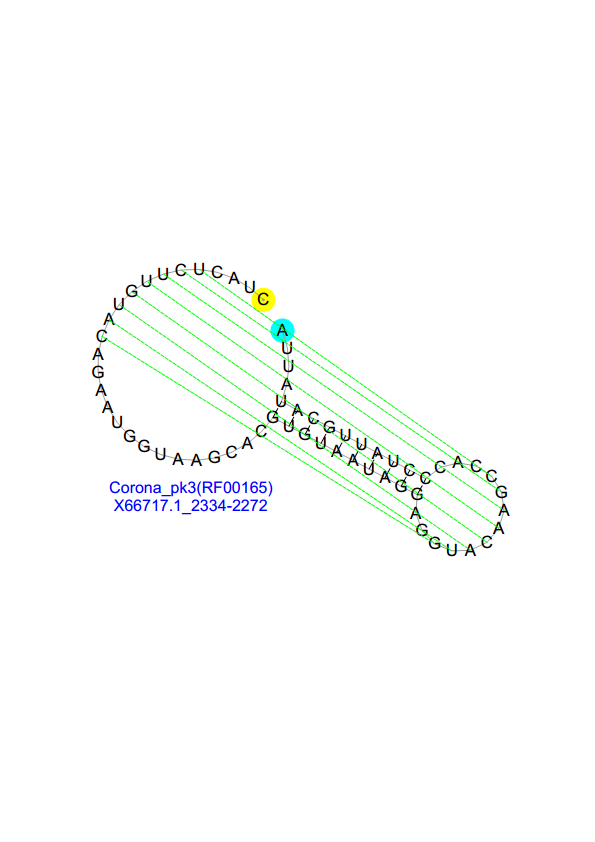


**(F)**


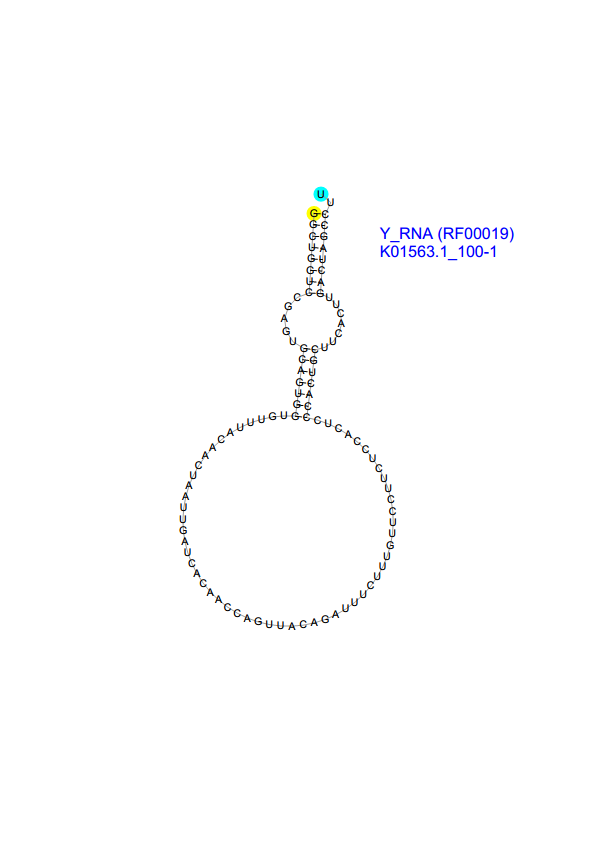

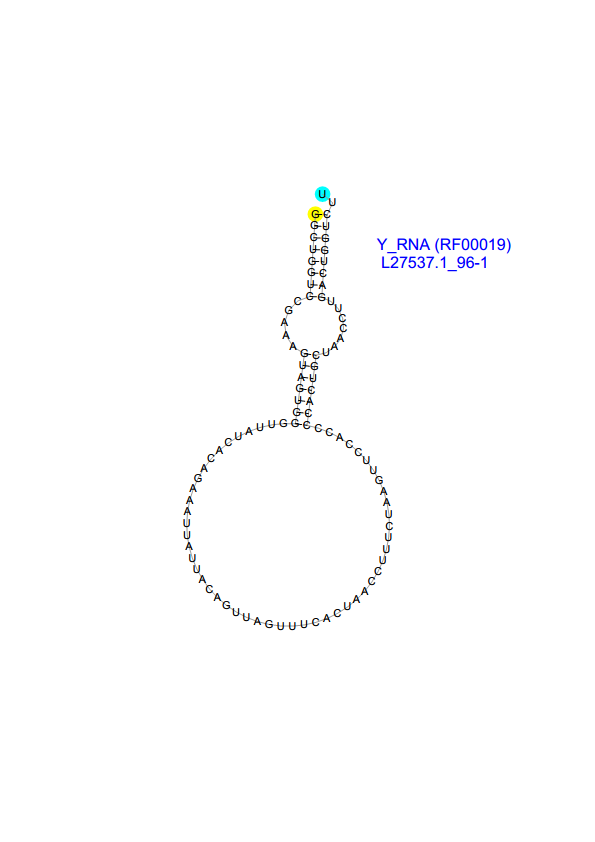

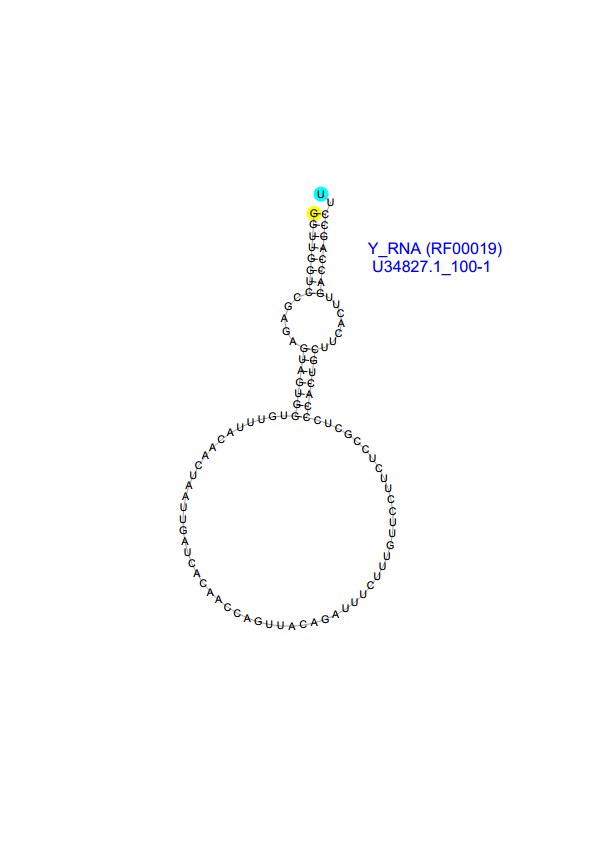


**(G)**


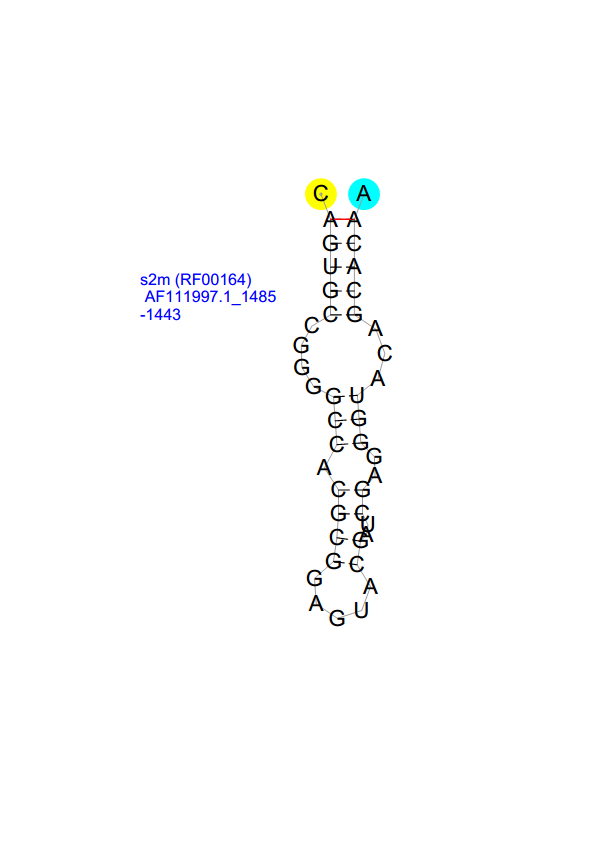

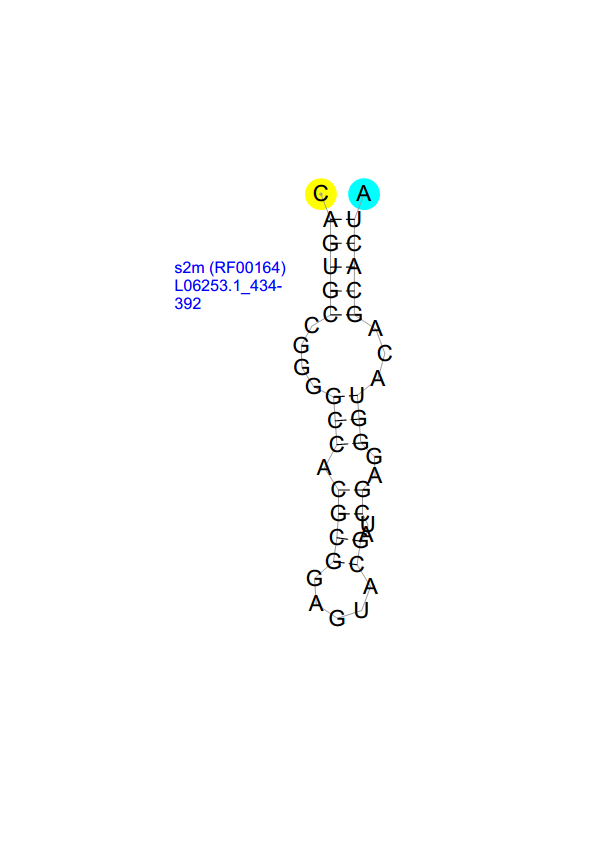

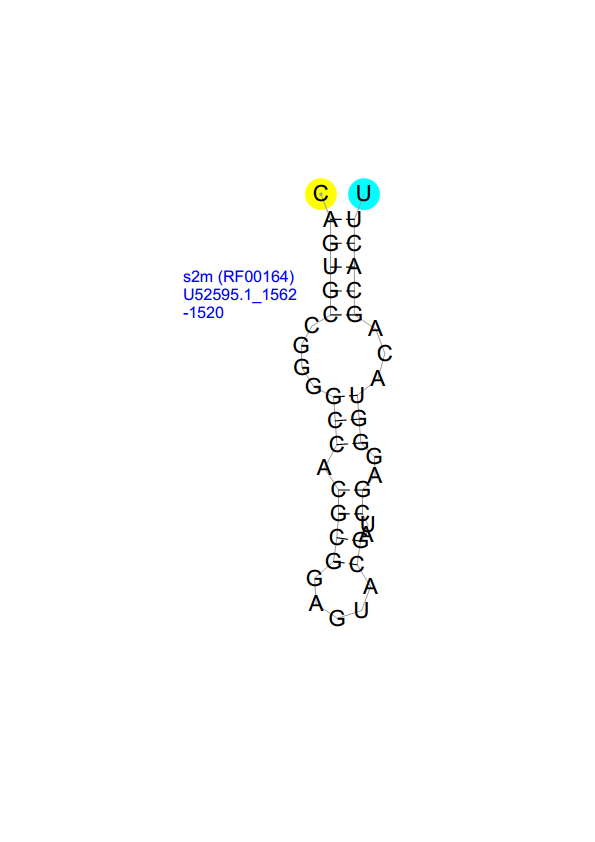

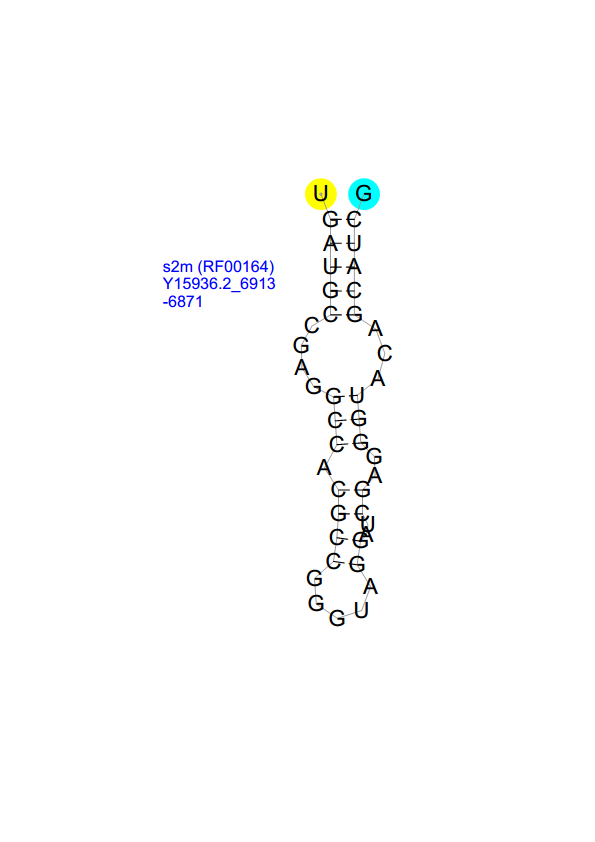


**(H)**


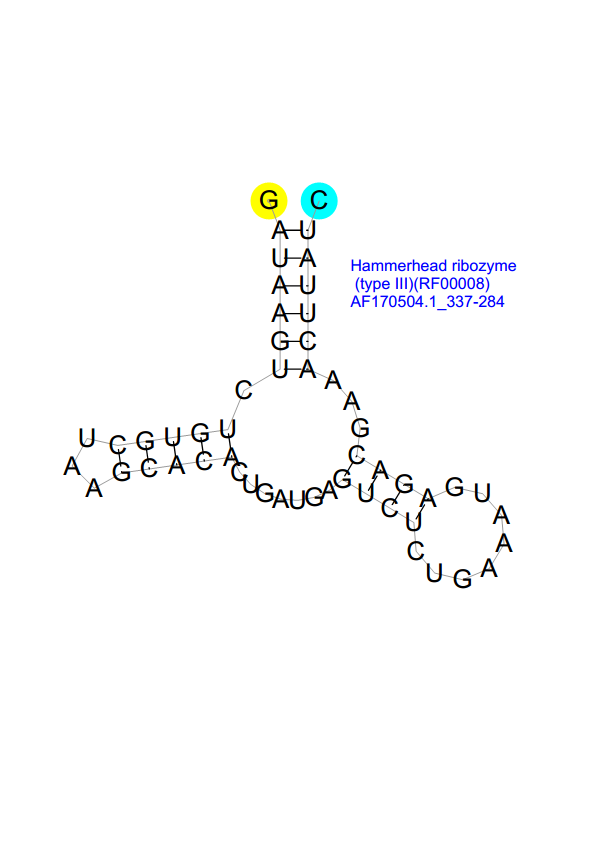

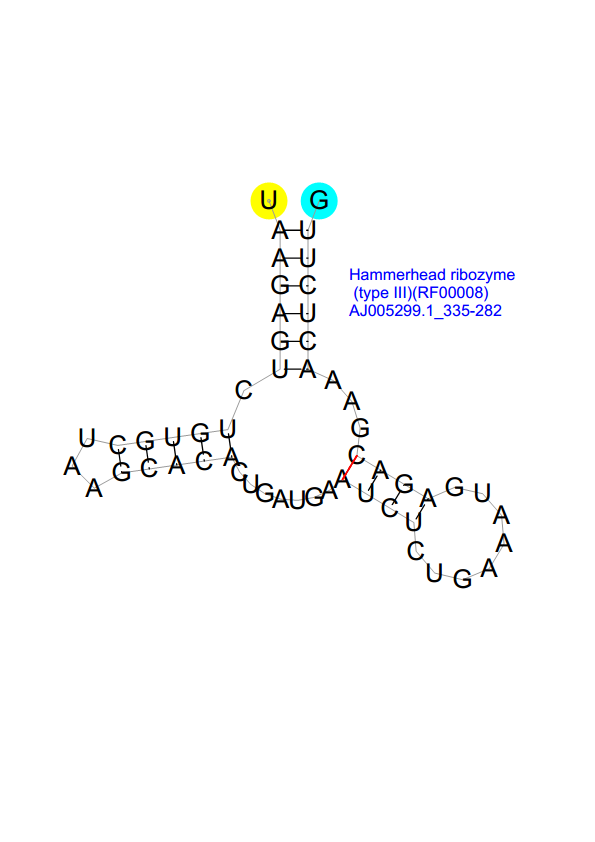

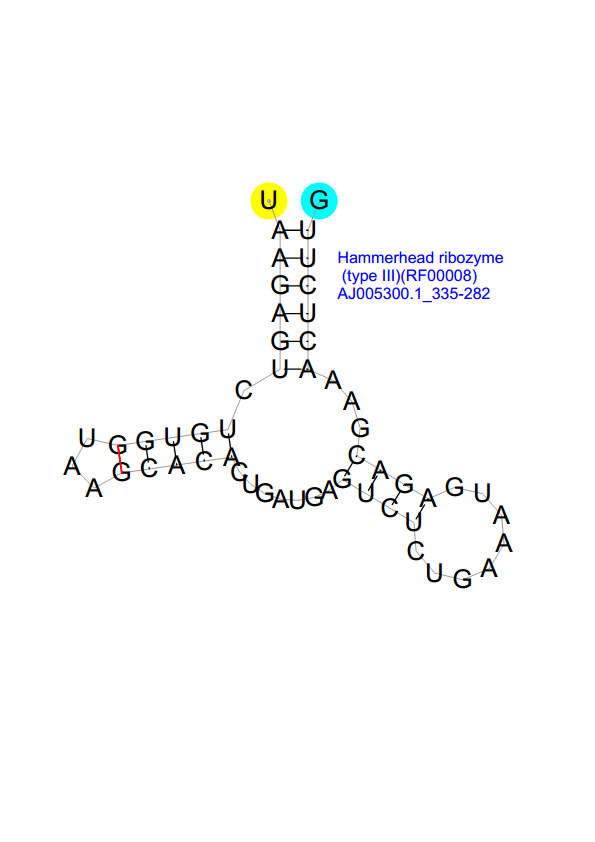

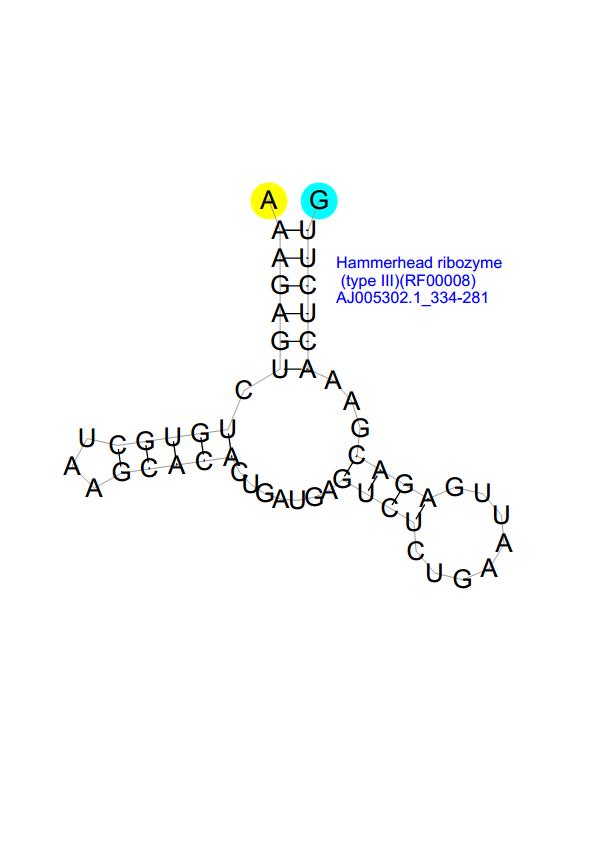

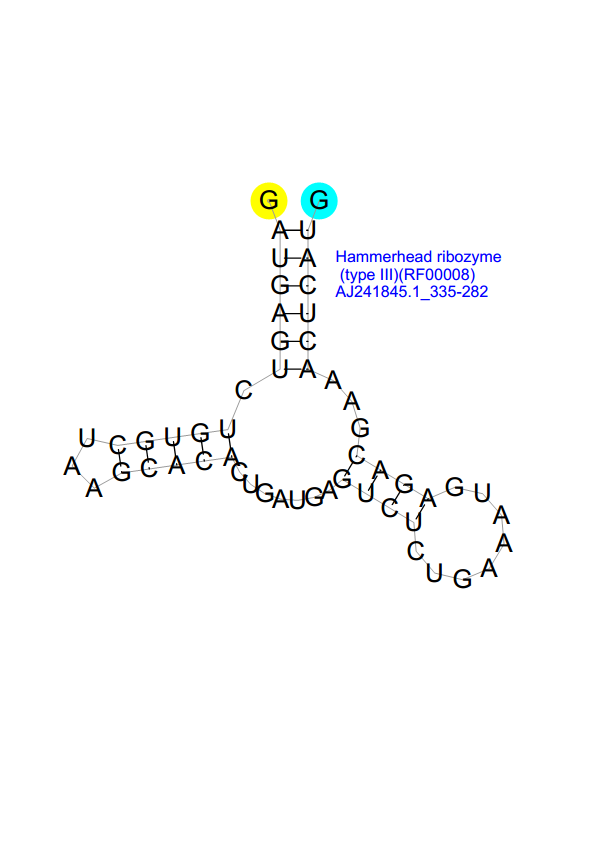

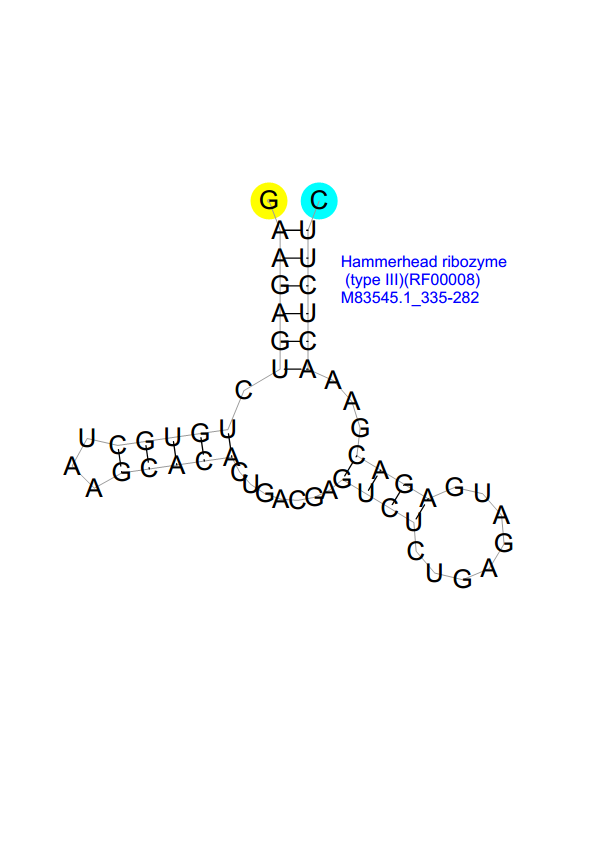


**(I)**


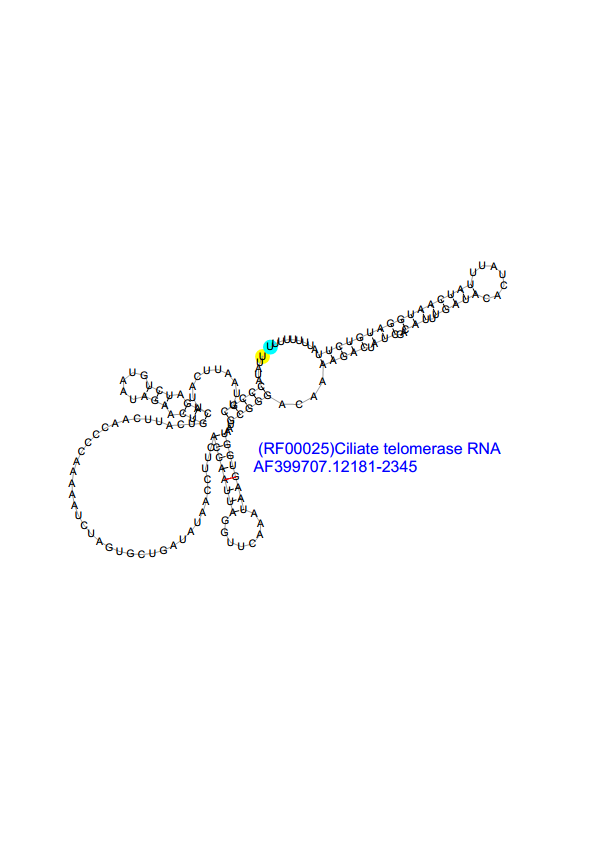

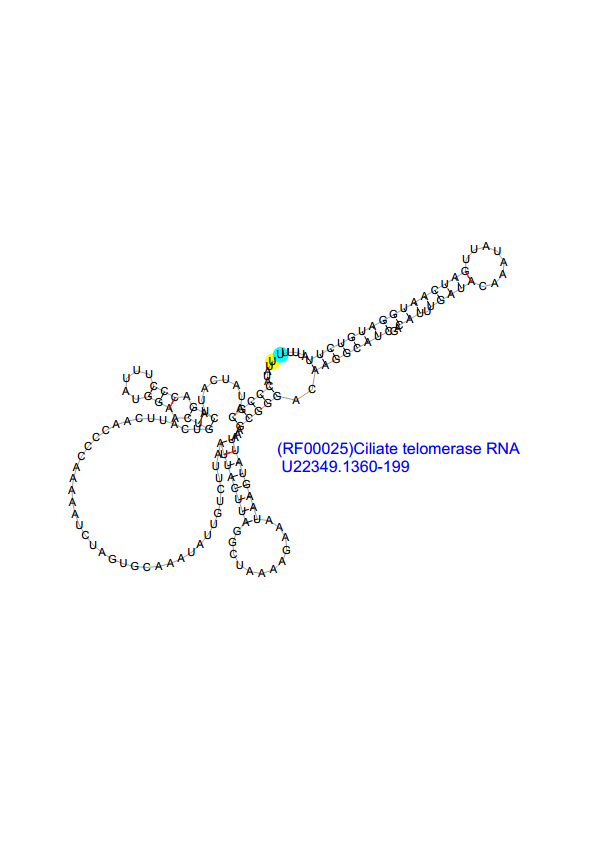

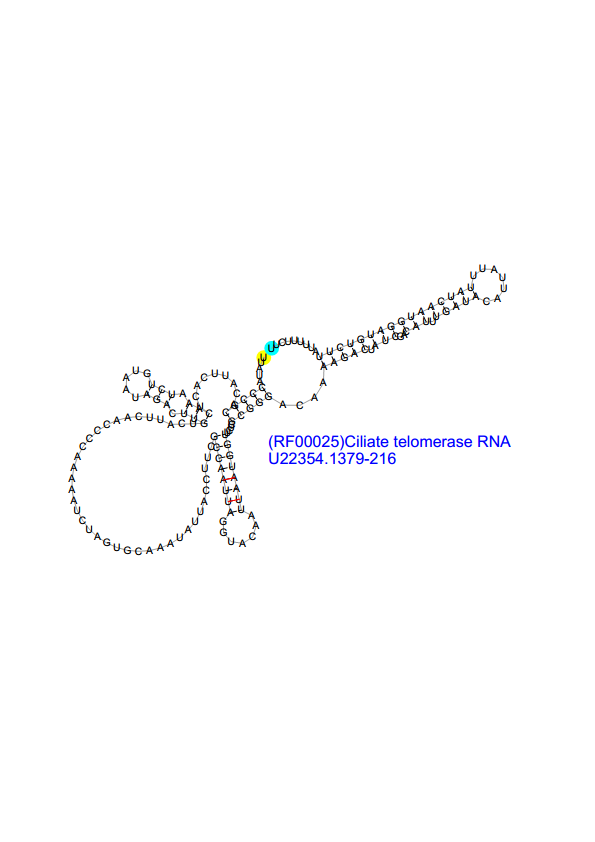


**(J)**


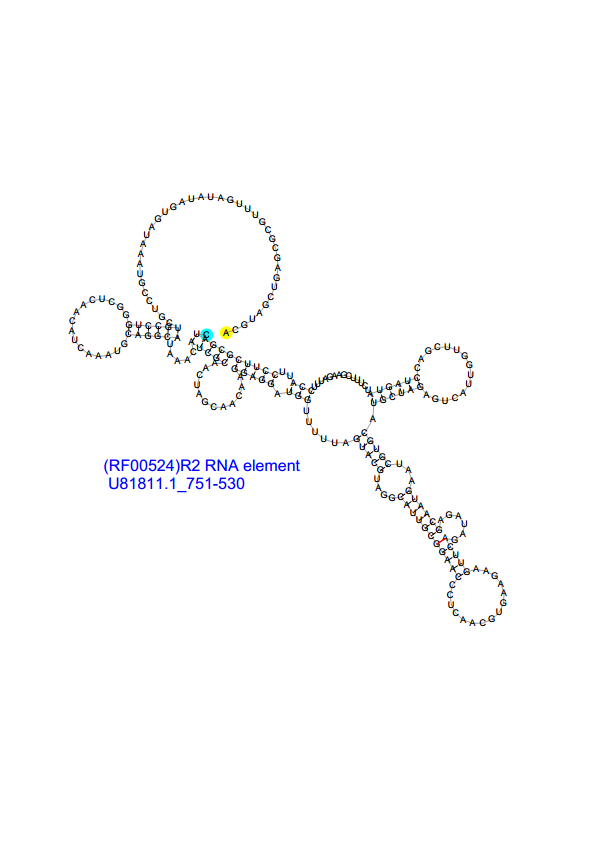

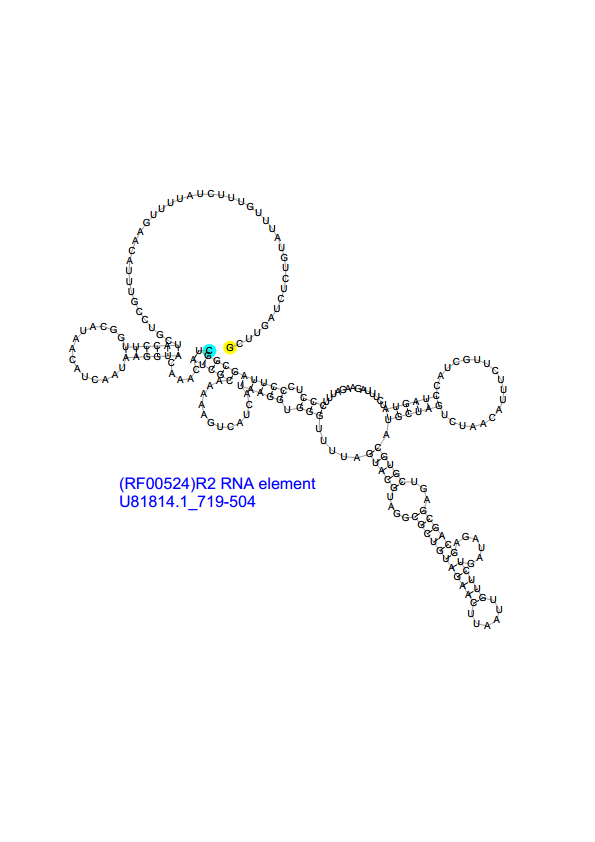


**(K)**


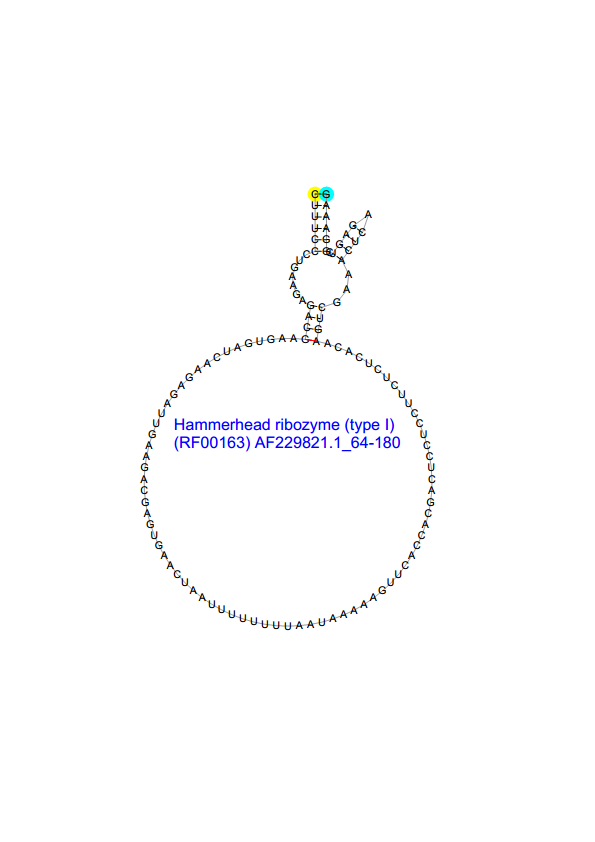

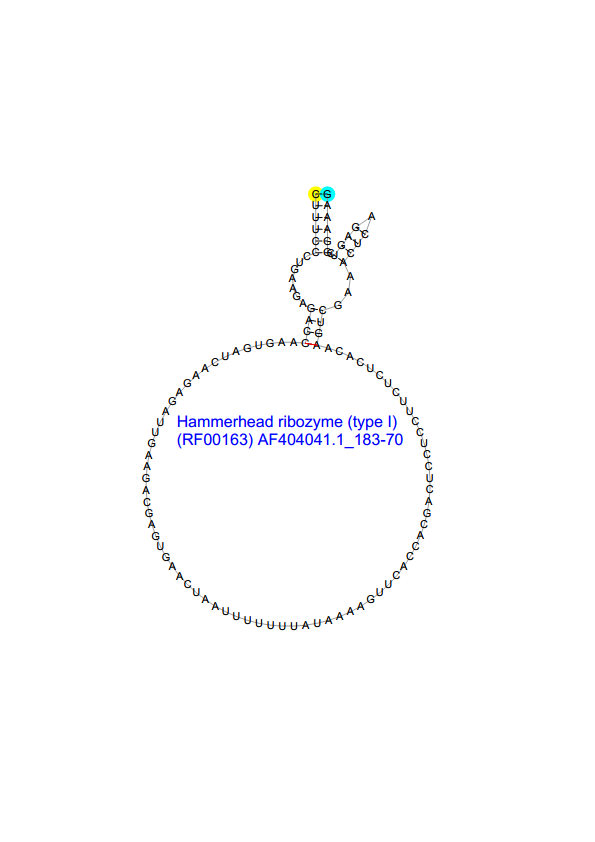

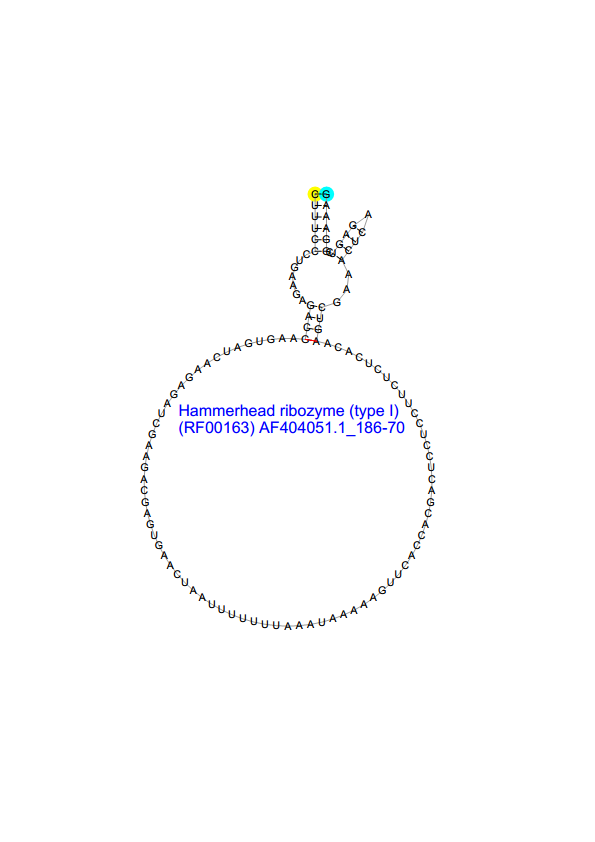

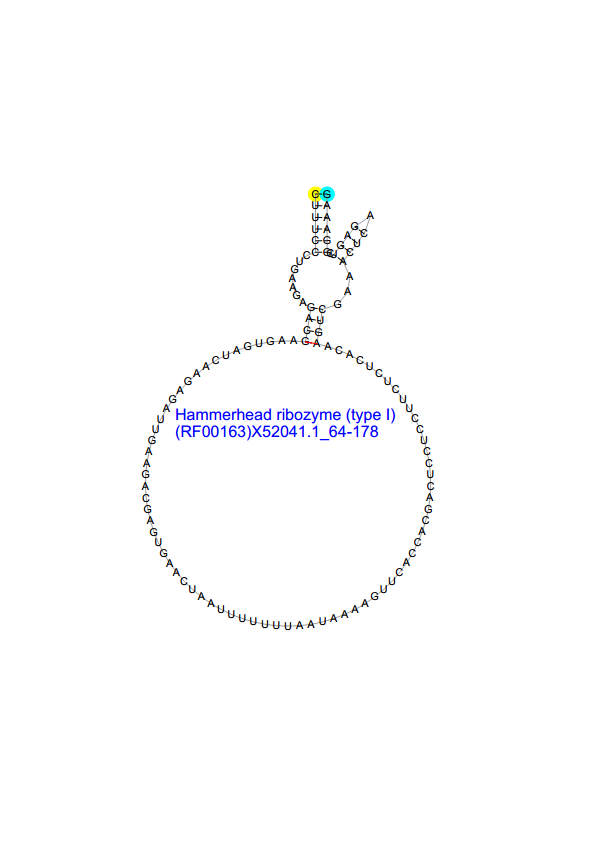


**(L)**


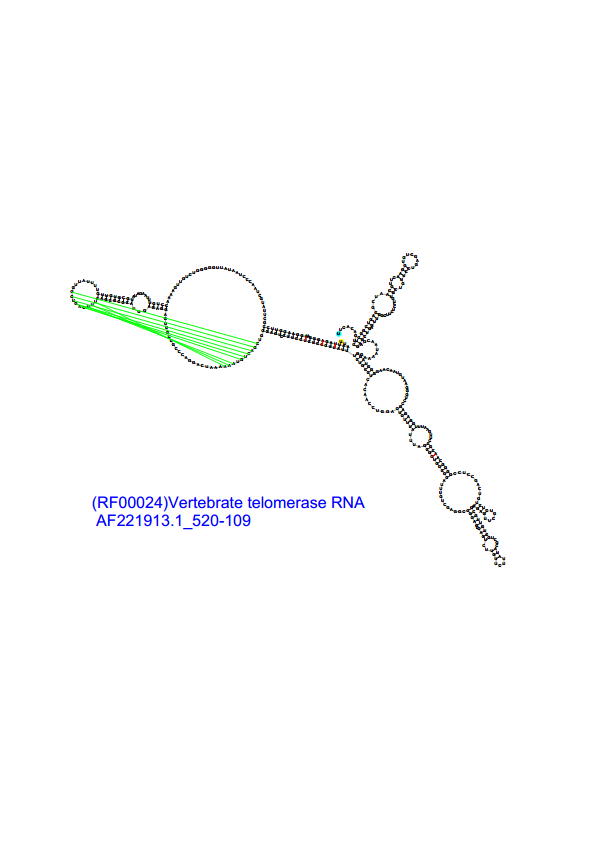

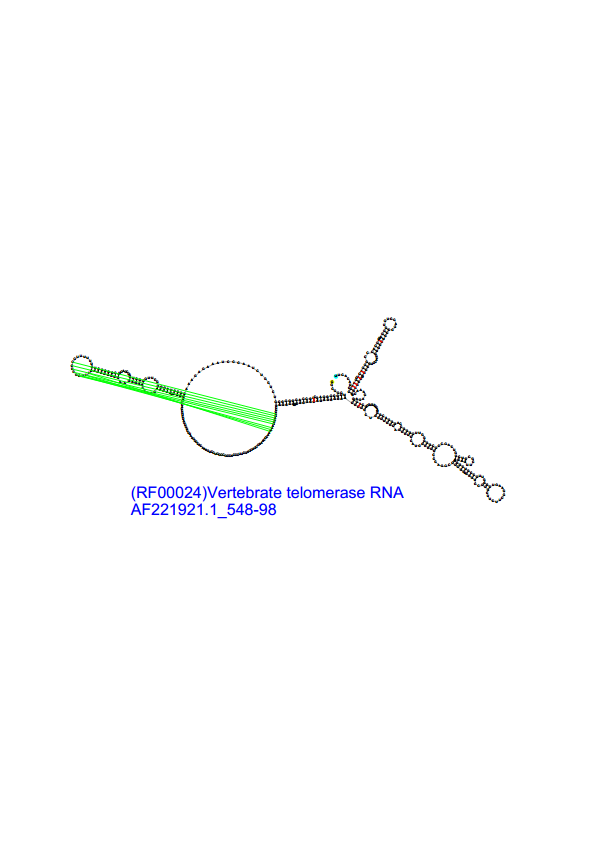

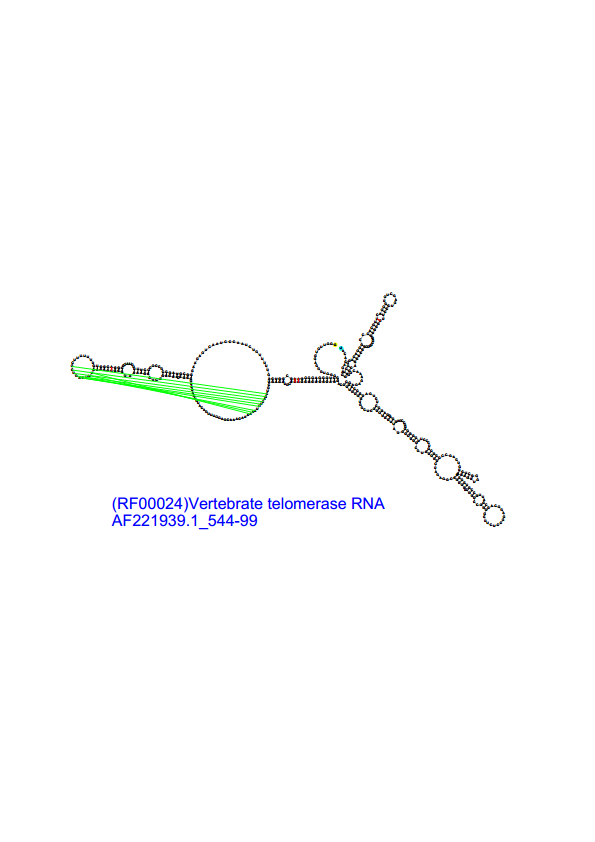

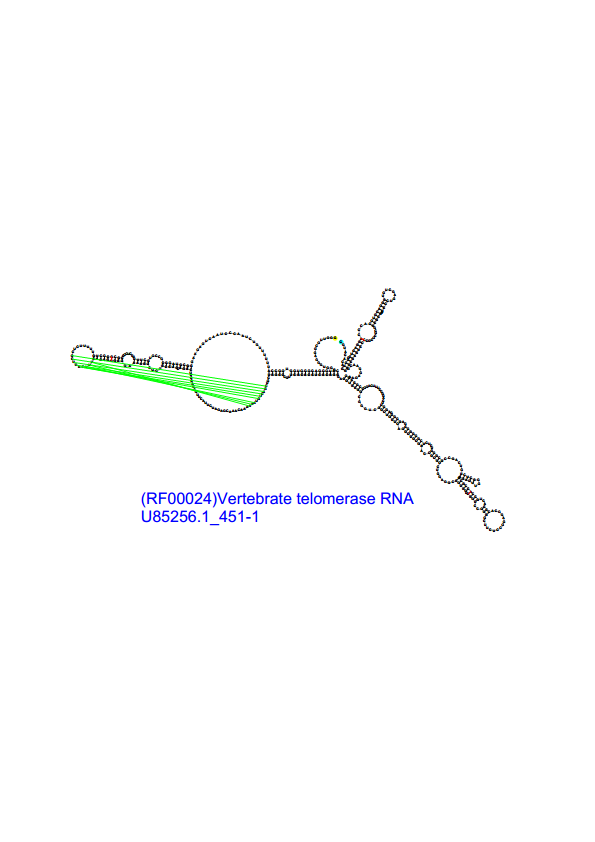


**(M)**


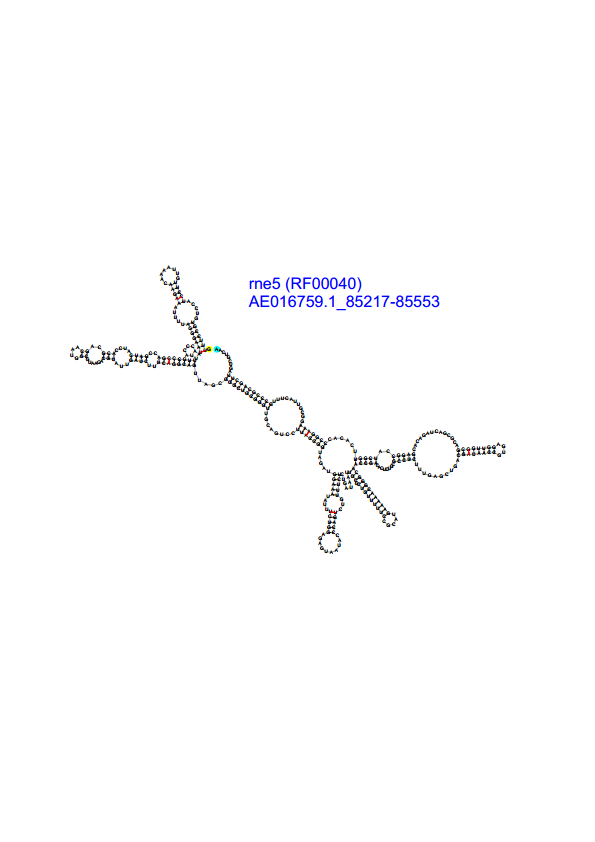

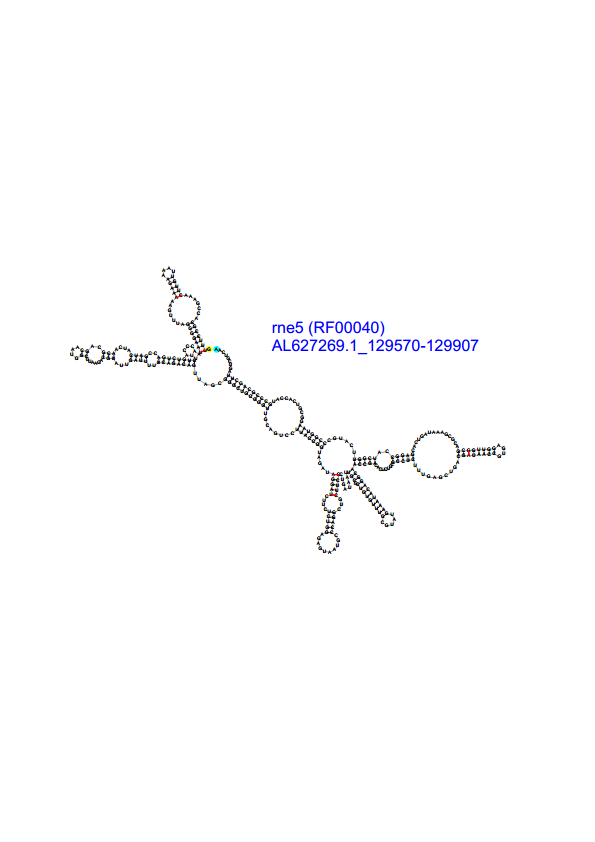

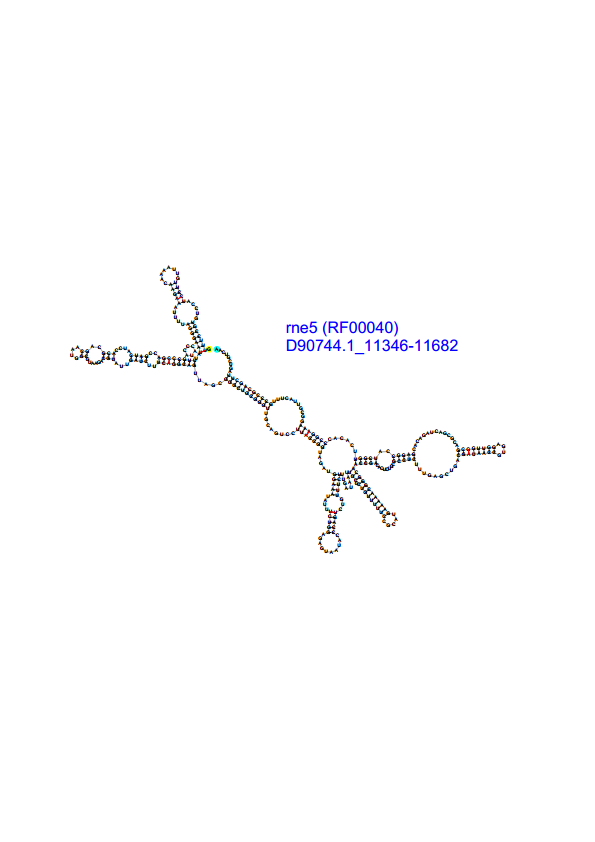


**(N)**


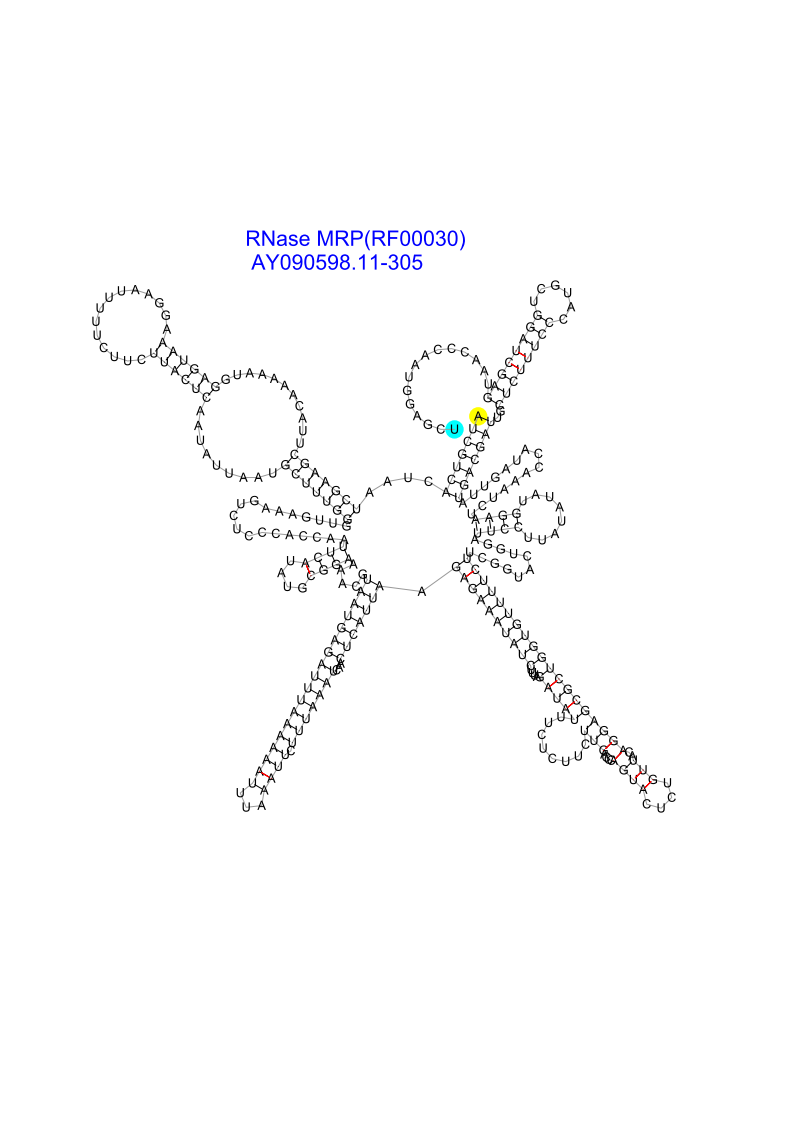

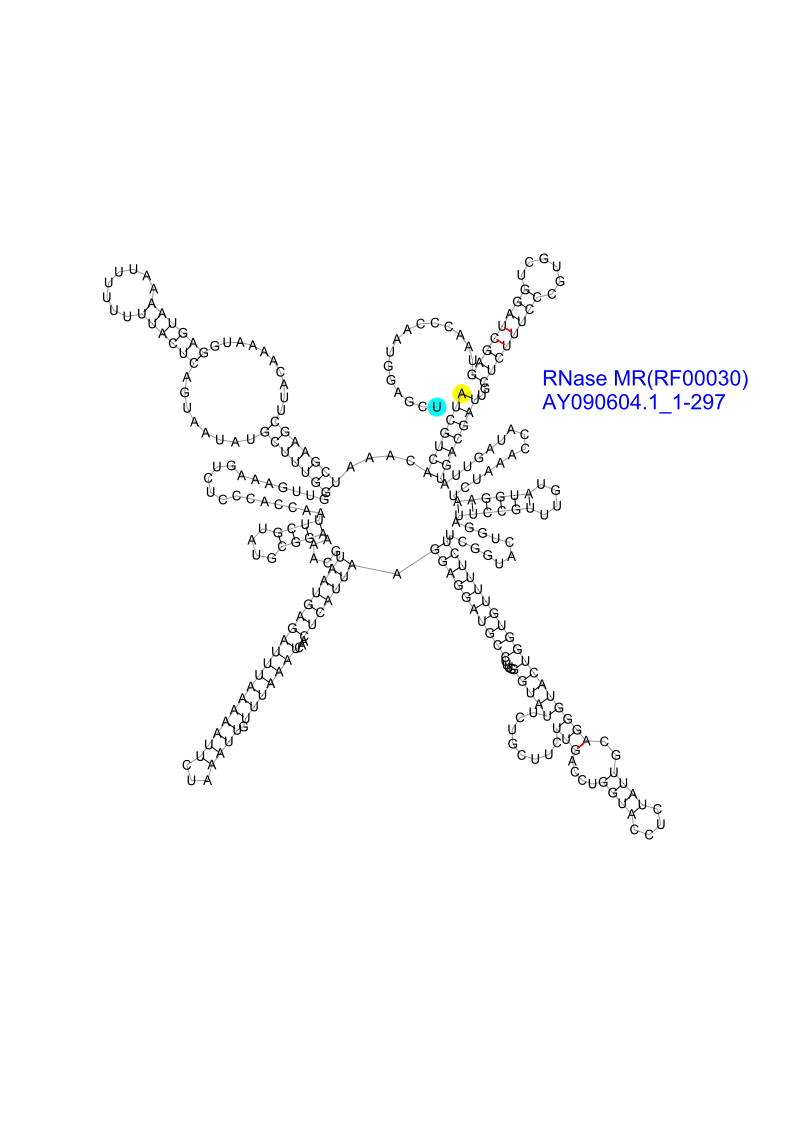

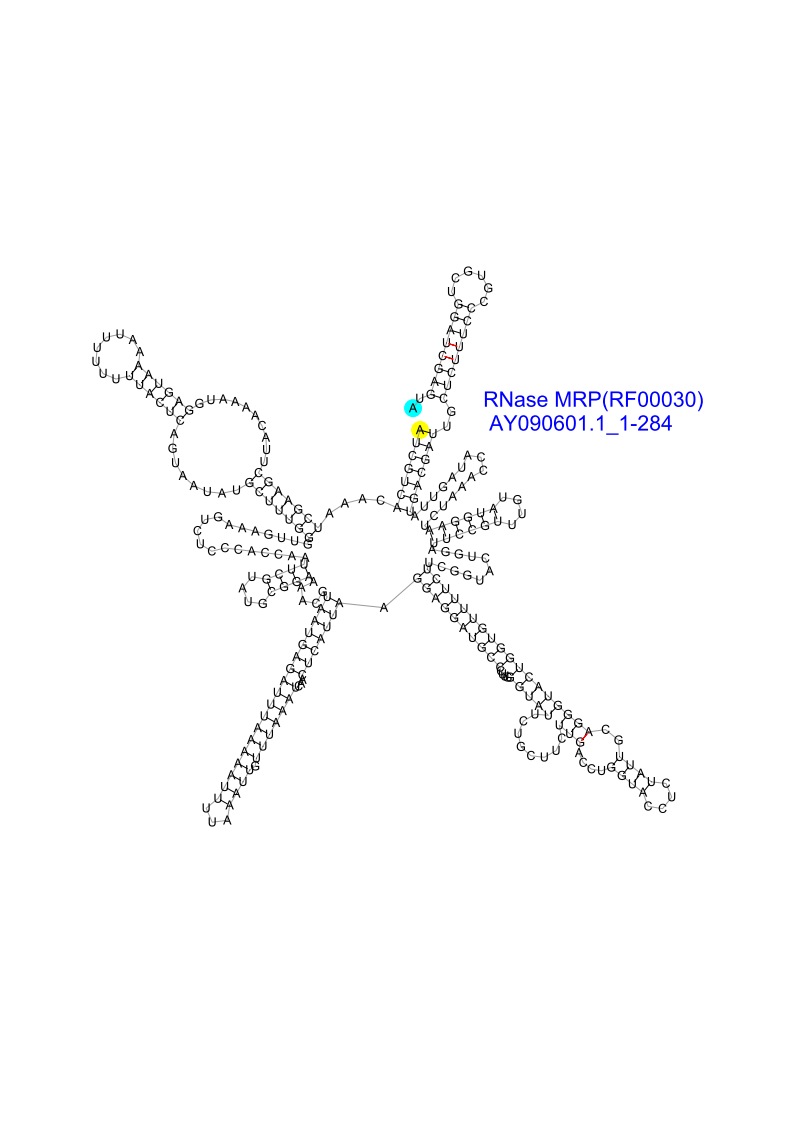


**(O)**

**
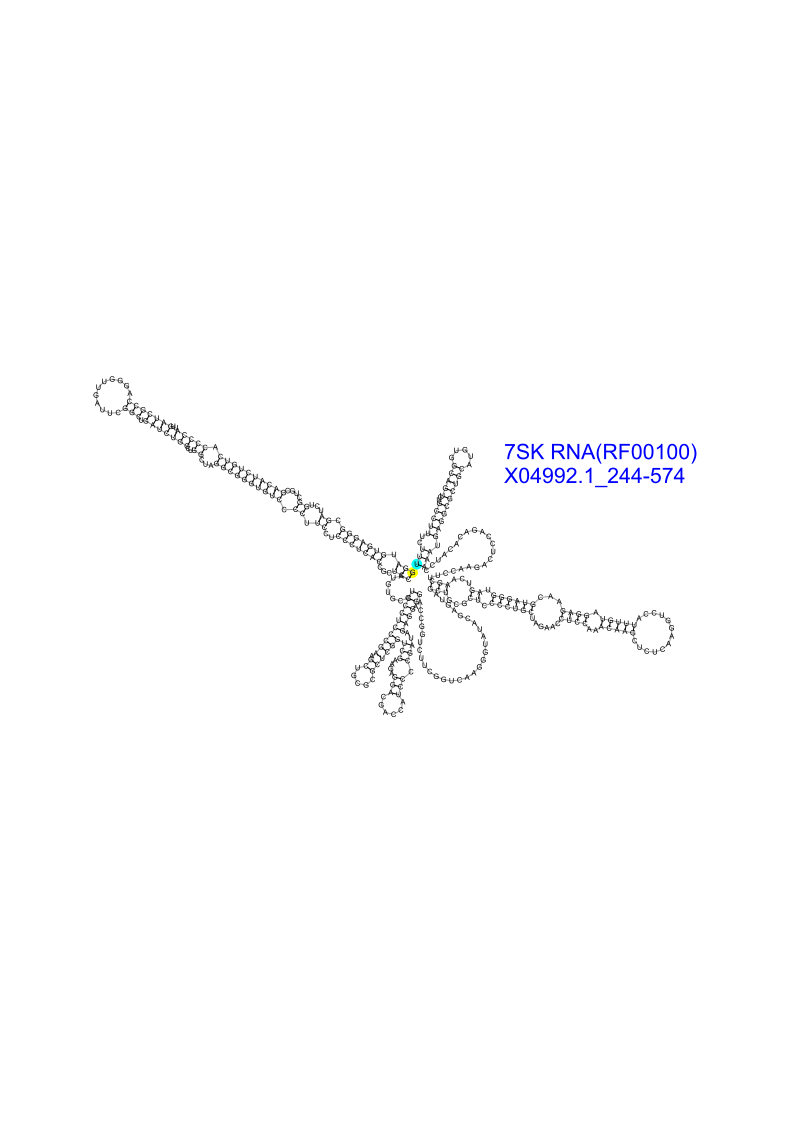
**

**(P)**


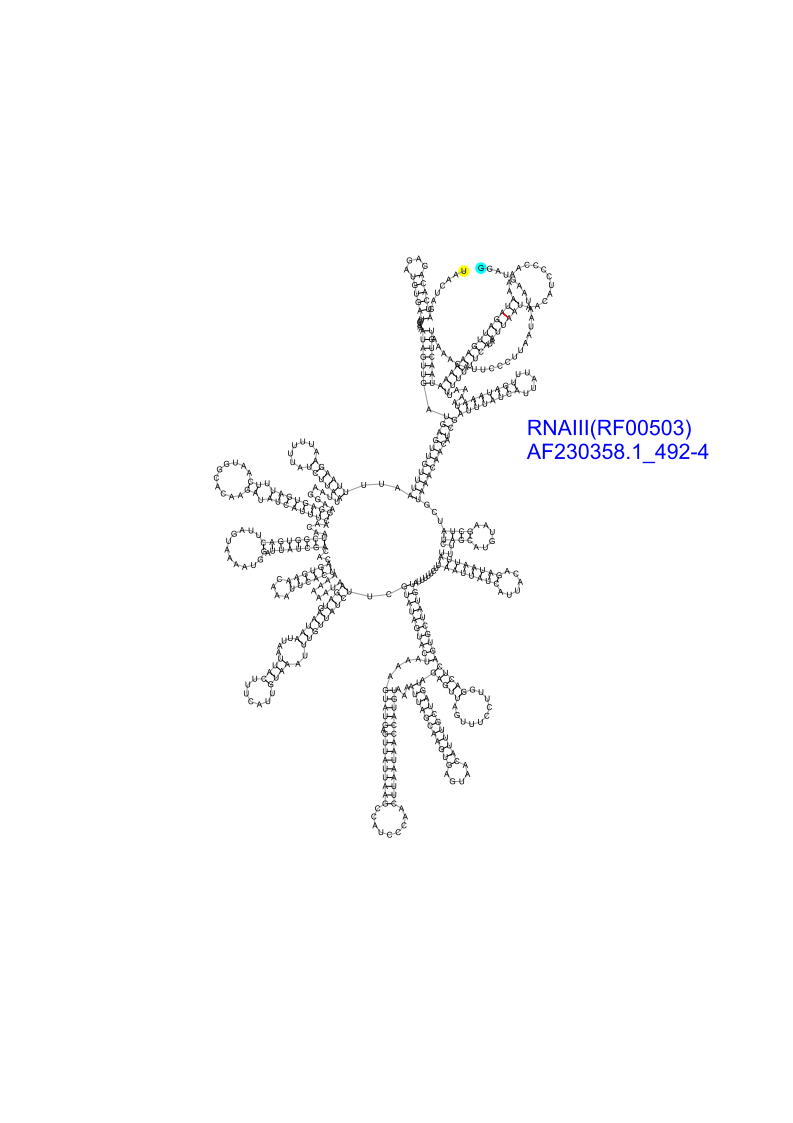

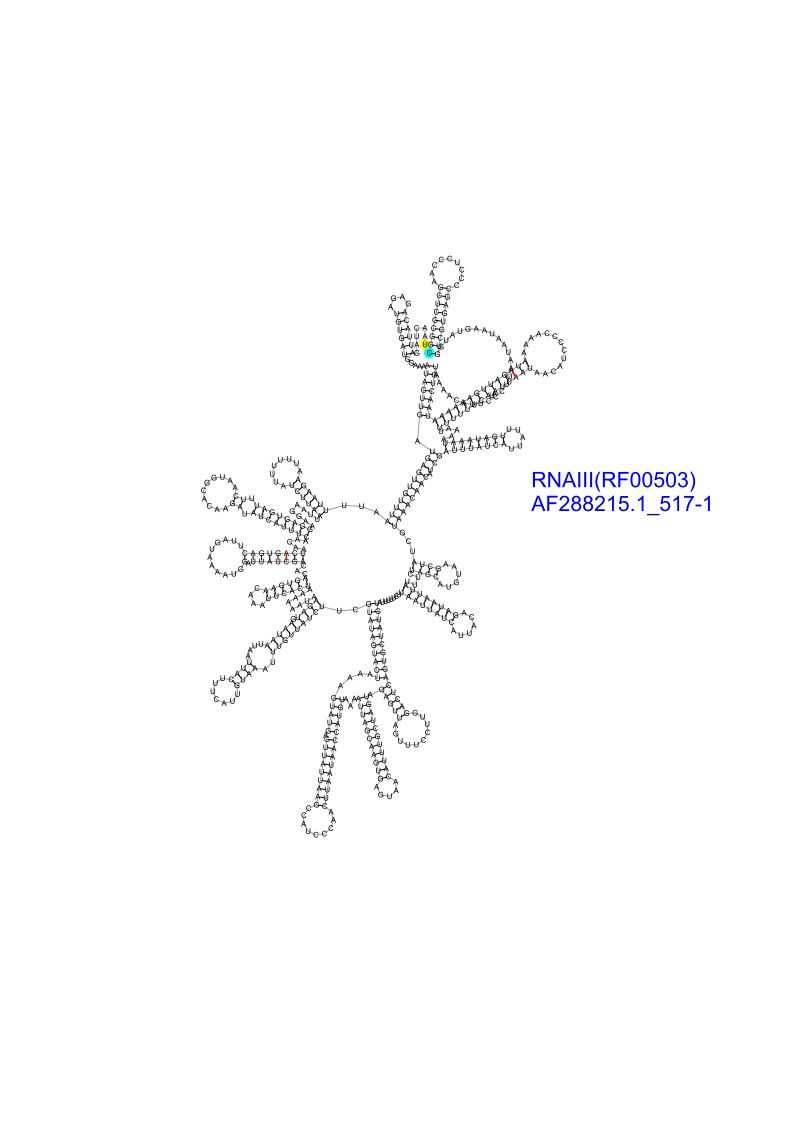

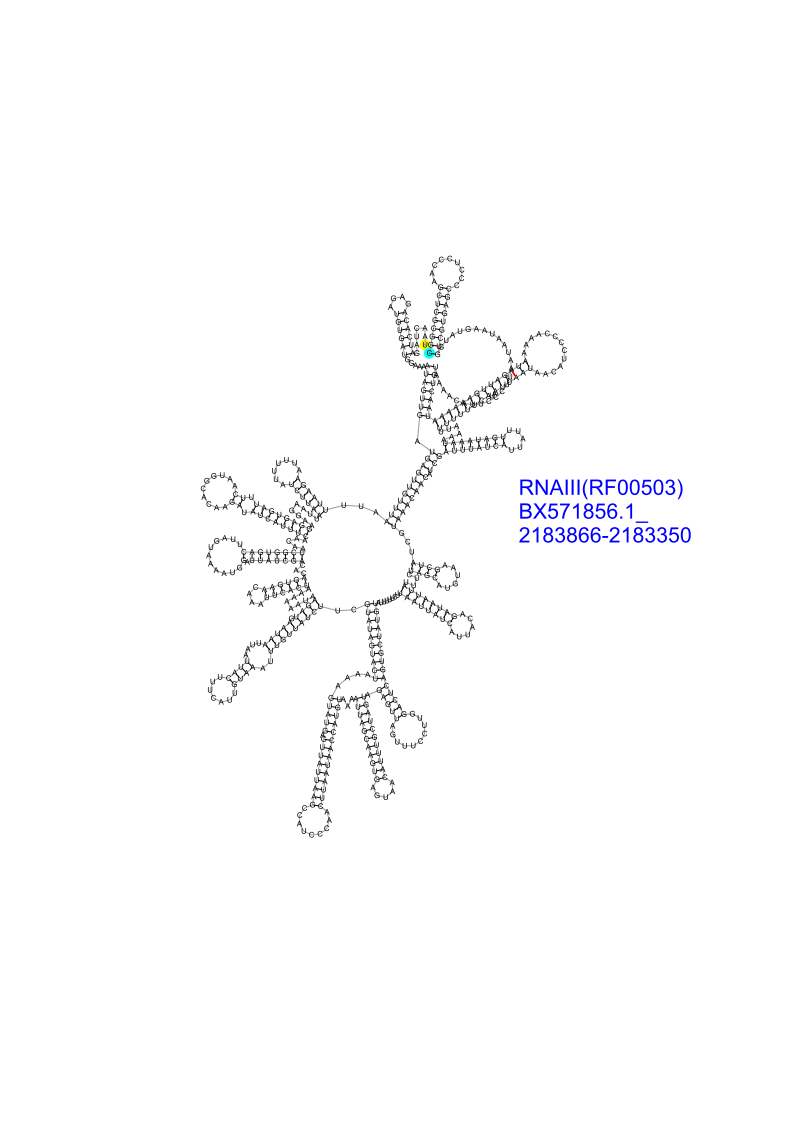


**(Q)**

**
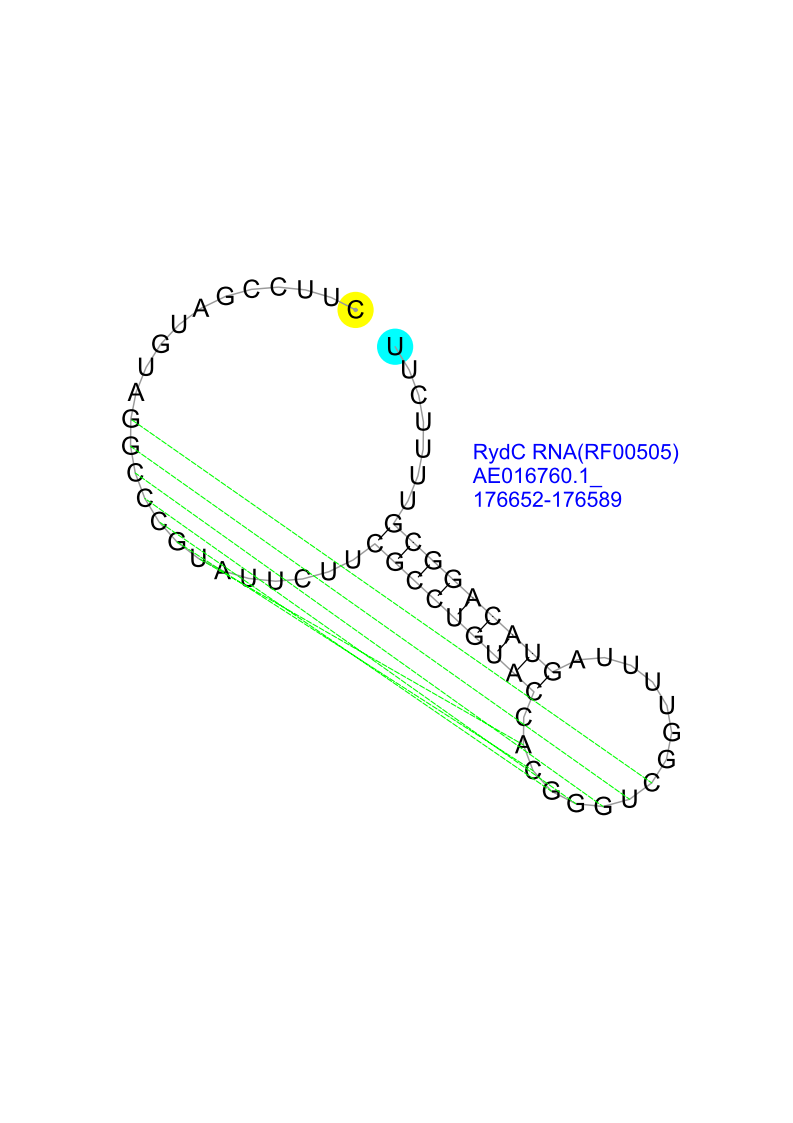

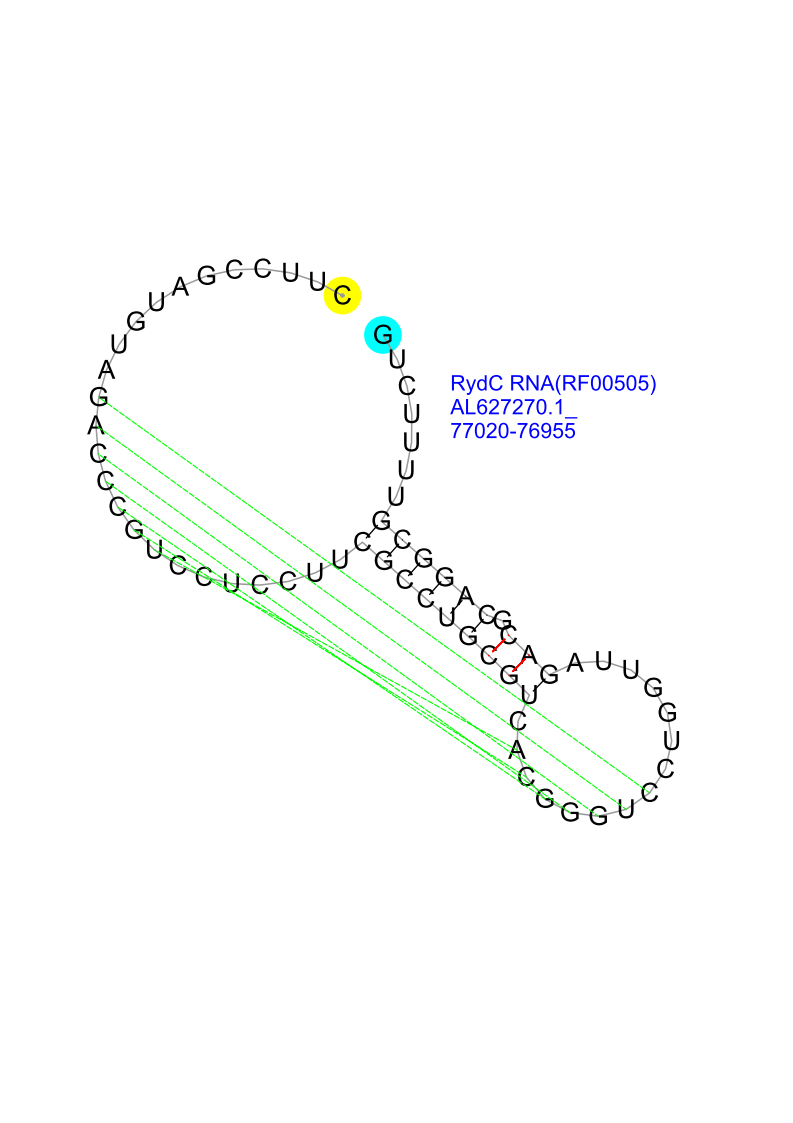
**

**(R)**

**
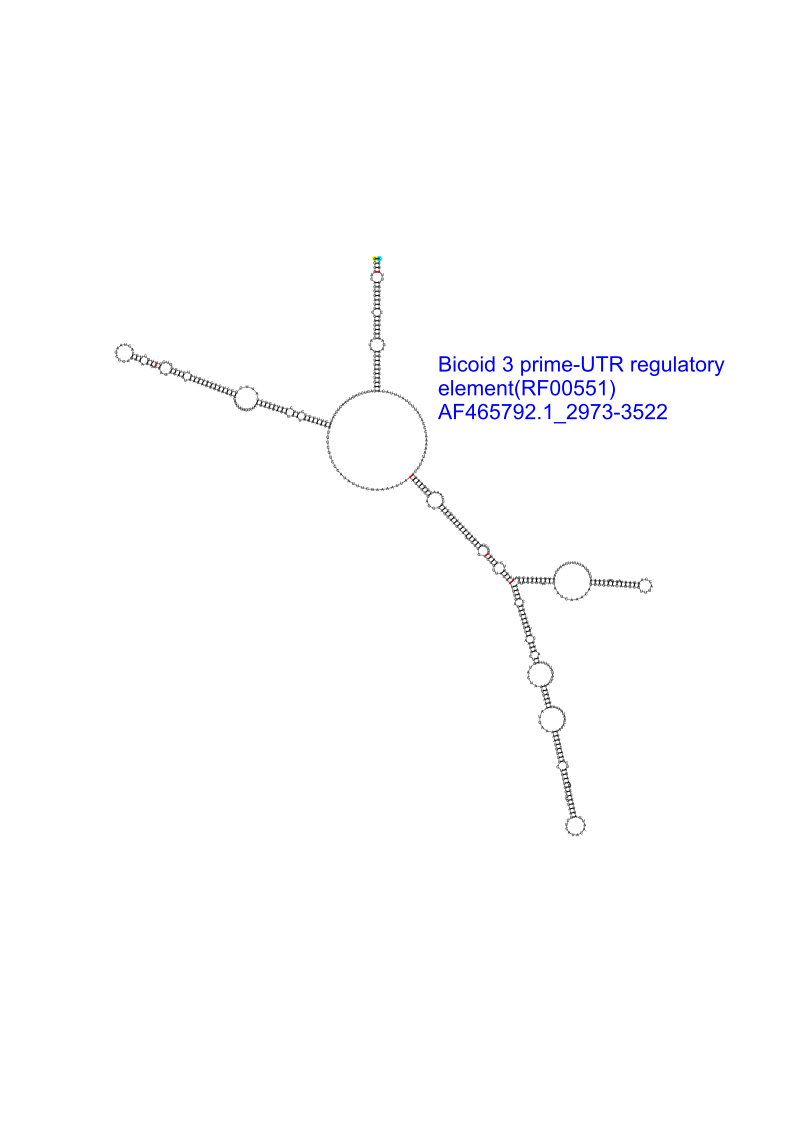

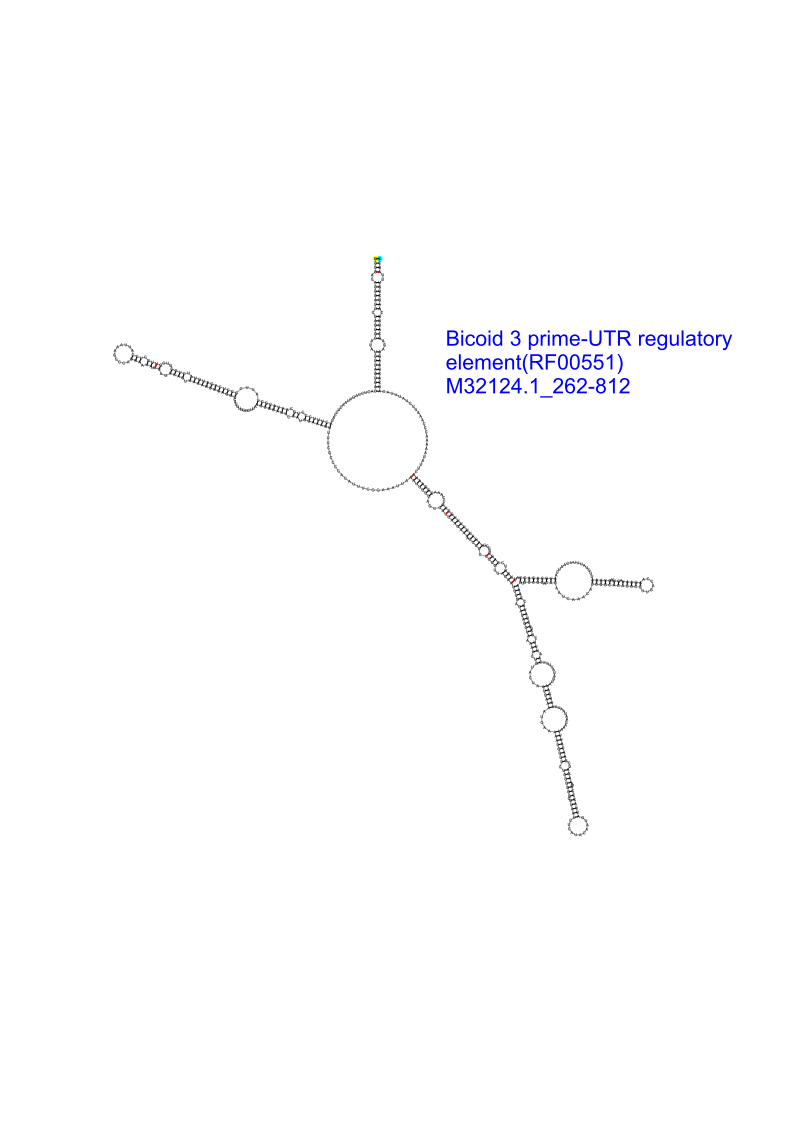

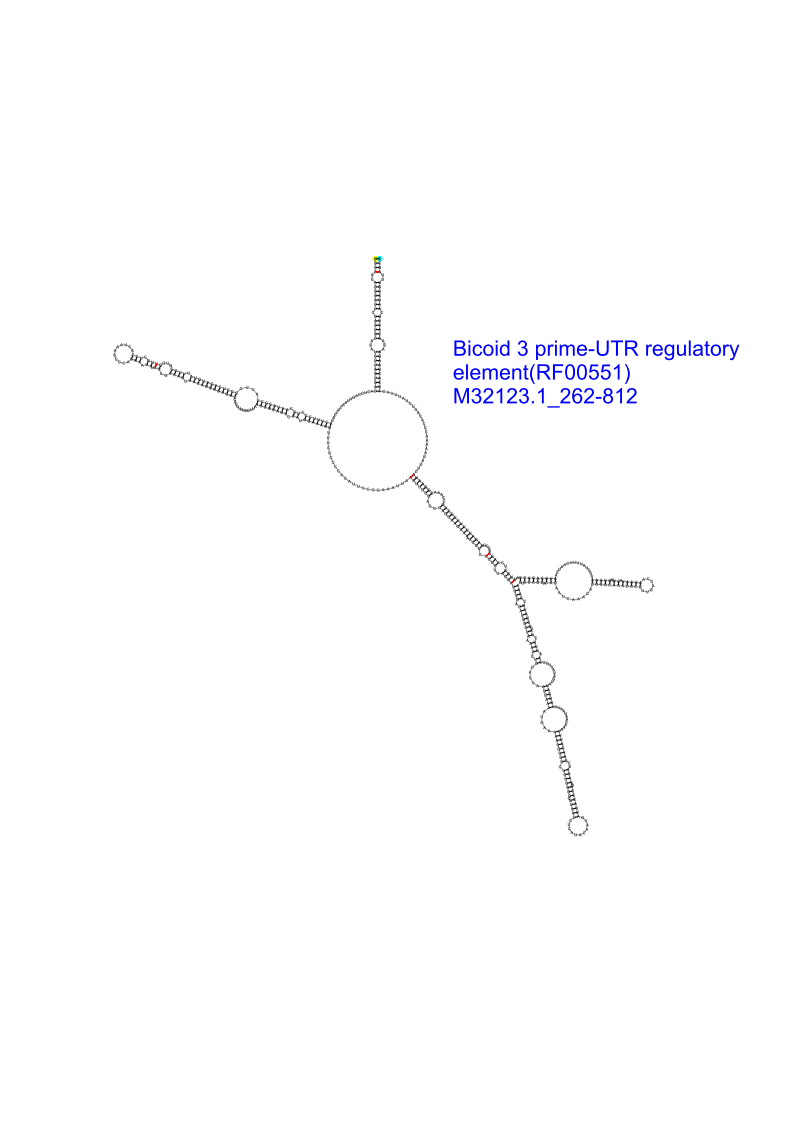
**
