# Supplementary material for: A Dynamic 3D Graphical Representation for RNA Structure Analysis and Its Application in Non-Coding RNA Classification
Source: PLoS One. 2016 May 23;11(5):e0152238. doi: 10.1371/journal.pone.0152238 (PMC4877074; doi:10.1371/journal.pone.0152238)

**S3 Fig. The Phylogenetic tree by multi-scale RNA comparison based on RNA triple vector curve representation for the secondary structures of RNAs in S1 Fig**.


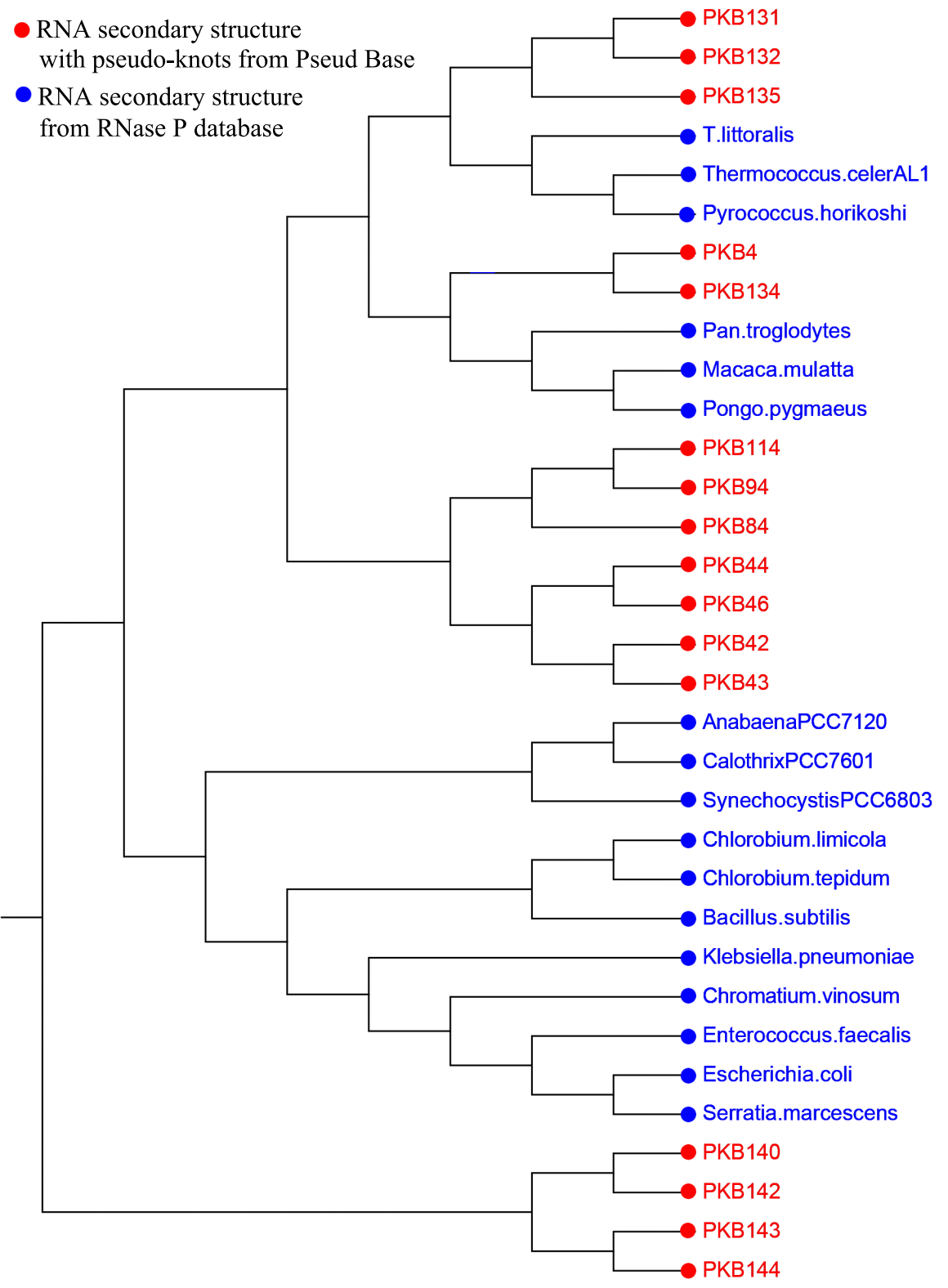

Supplement: S3 Fig — (DOC) [file pone.0152238.s003.doc]
