# Supplementary material for: A Dynamic 3D Graphical Representation for RNA Structure Analysis and Its Application in Non-Coding RNA Classification
Source: PLoS One. 2016 May 23;11(5):e0152238. doi: 10.1371/journal.pone.0152238 (PMC4877074; doi:10.1371/journal.pone.0152238)
Supplement: S4 Fig — (DOC) [file pone.0152238.s004.doc]

**S4 Fig. The Phylogenetic tree by multi-scale RNA comparison based on RNA triple vector curve representation for the secondary structures of RNAs in S2 Fig.**

**
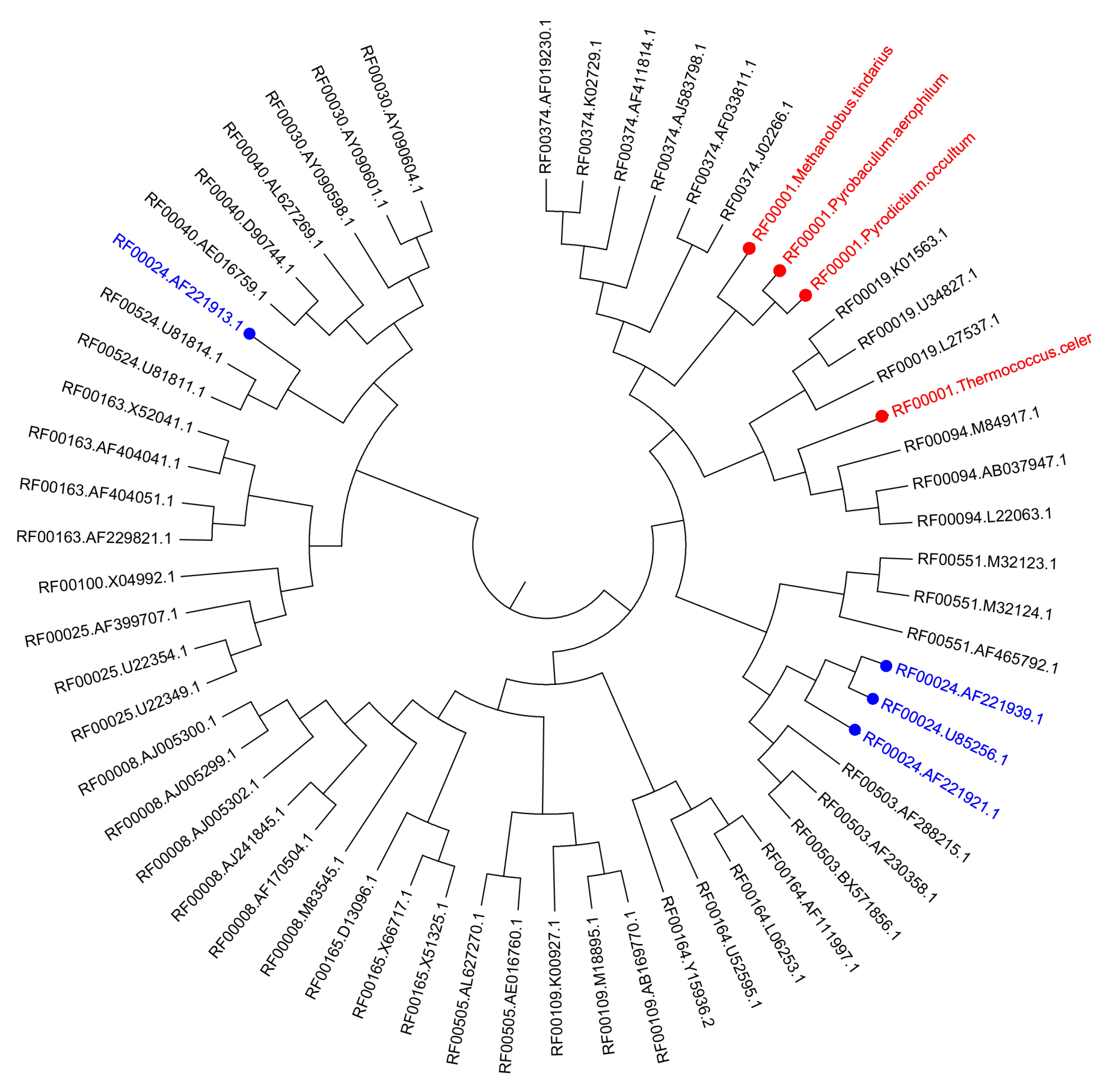
**
