# Supplementary material for: A Dynamic 3D Graphical Representation for RNA Structure Analysis and Its Application in Non-Coding RNA Classification
Source: PLoS One. 2016 May 23;11(5):e0152238. doi: 10.1371/journal.pone.0152238 (PMC4877074; doi:10.1371/journal.pone.0152238)

**S6 Fig. The two phylogenetic trees for the secondary structures of RNAs in S2 Fig based on the method by Liao *et al* [34].** (A) The phylogenetic tree based on the Euclidean. (B) The phylogenetic tree based on the Angle.

**(A)**


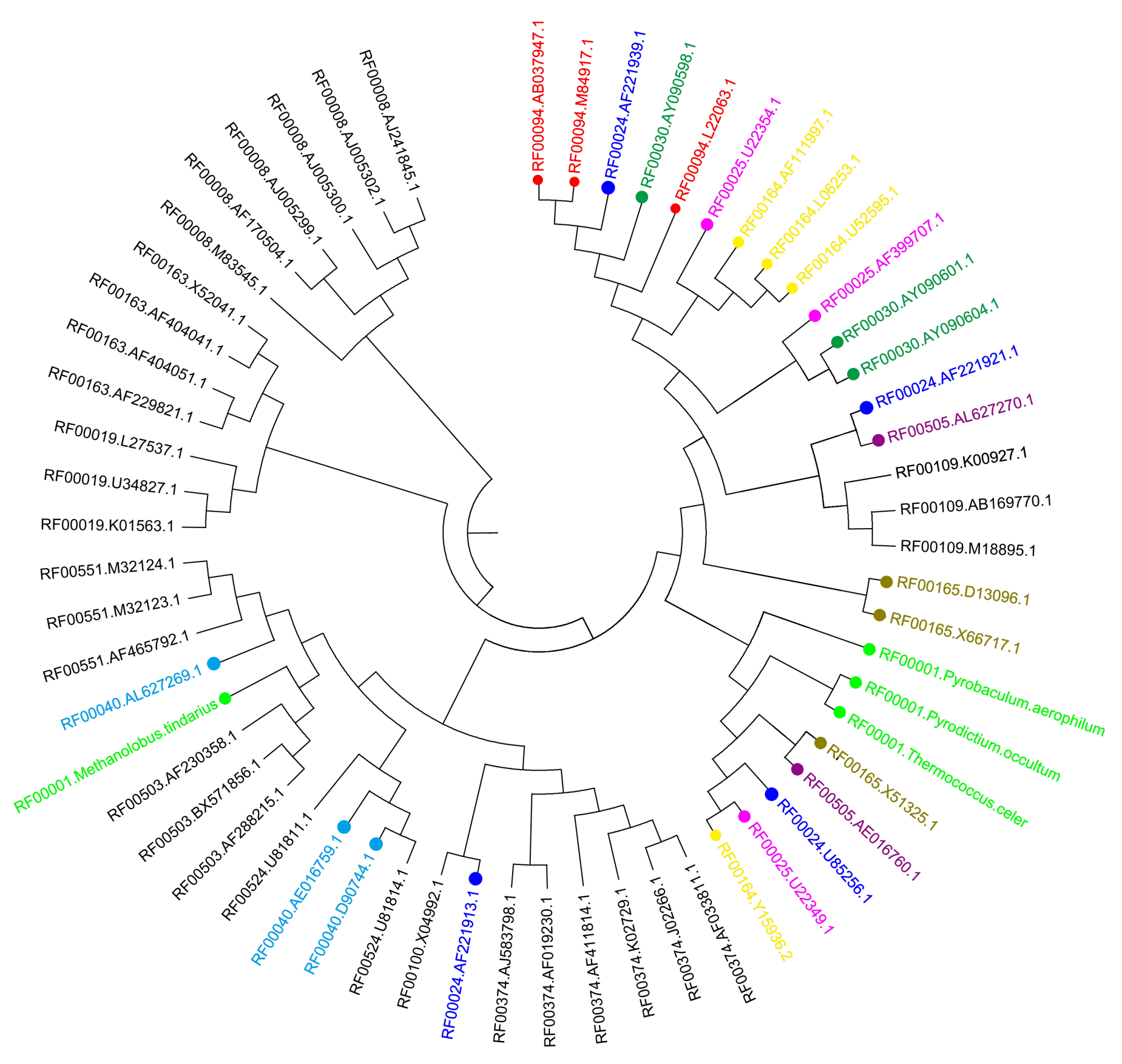


**(B)**


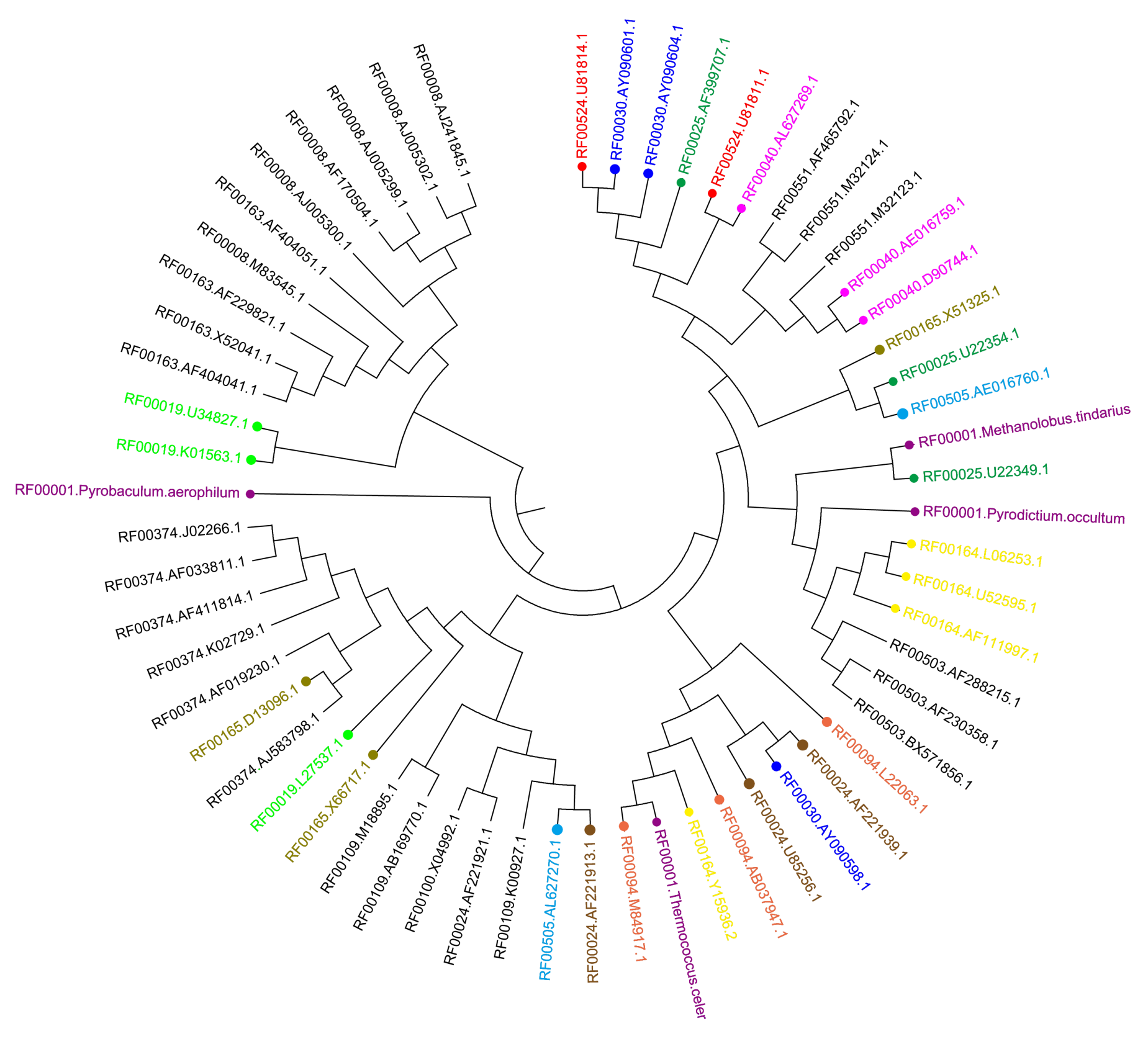

Supplement: S6 Fig — (A) The phylogenetic tree based on the Euclidean. (B) The phylogenetic tree based on the Angle. (DOC) [file pone.0152238.s006.doc]
