# Supplementary material for: A Dynamic 3D Graphical Representation for RNA Structure Analysis and Its Application in Non-Coding RNA Classification
Source: PLoS One. 2016 May 23;11(5):e0152238. doi: 10.1371/journal.pone.0152238 (PMC4877074; doi:10.1371/journal.pone.0152238)

**S7 Fig. The two phylogenetic trees for the secondary structures of RNAs in S1 Fig based on the method by Zhu *et al* [25].** (A) The phylogenetic tree based on the Euclidean distance. (B) The phylogenetic tree based on the Angle.

**(A)**


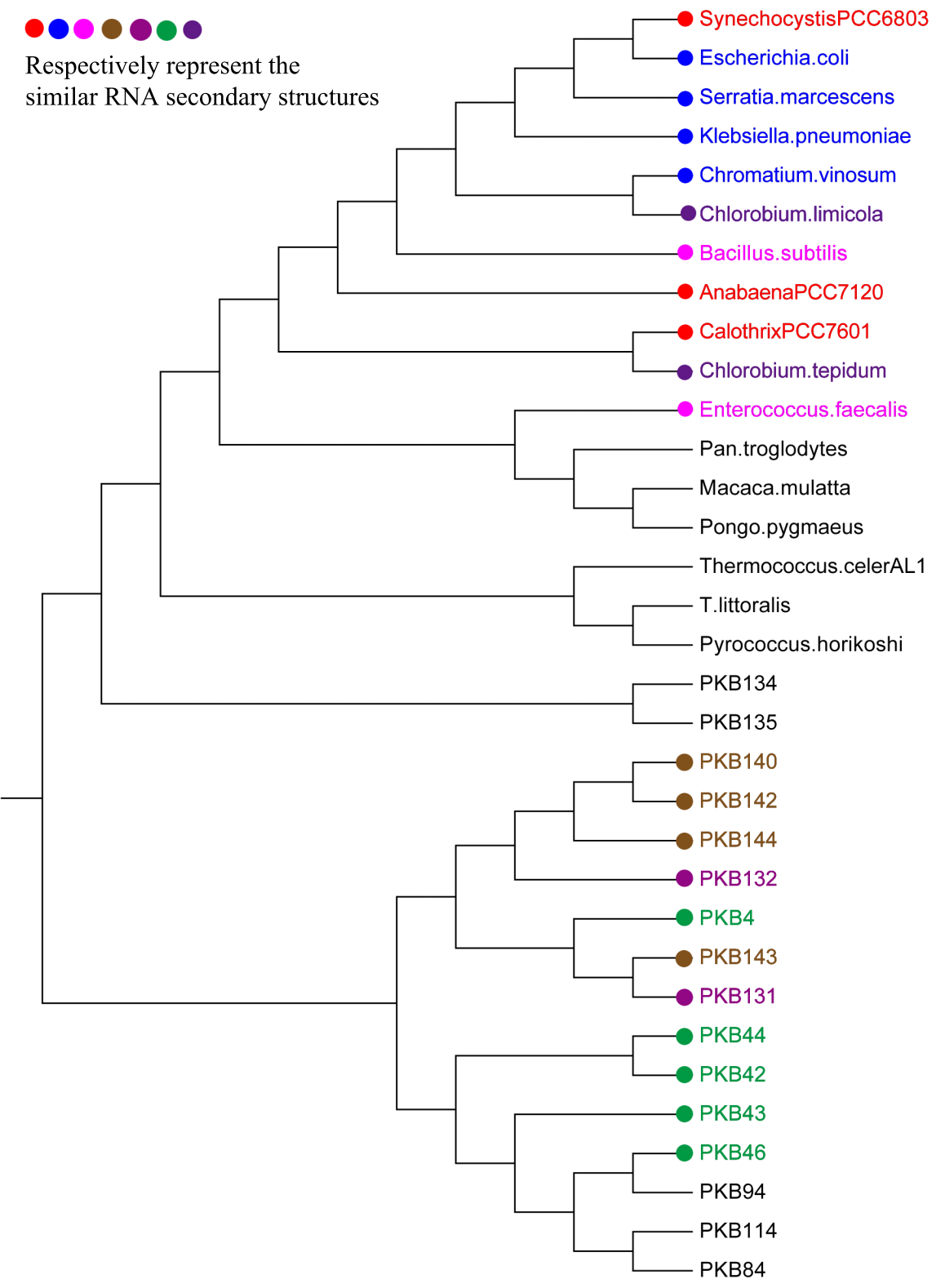


**(B)**


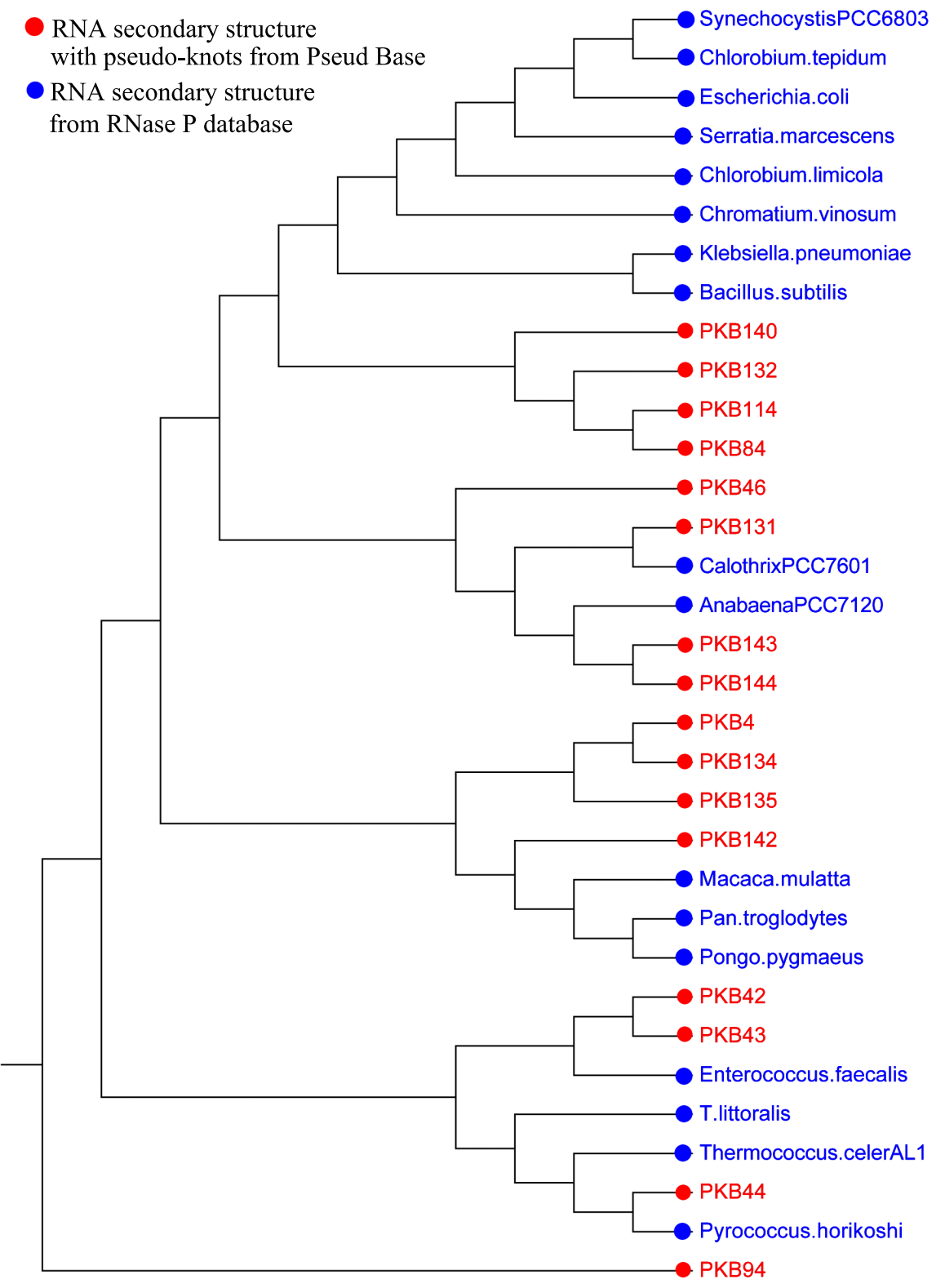

Supplement: S7 Fig — (A) The phylogenetic tree based on the Euclidean distance. (B) The phylogenetic tree based on the Angle. (DOC) [file pone.0152238.s007.doc]
