# Supplementary material for: A Dynamic 3D Graphical Representation for RNA Structure Analysis and Its Application in Non-Coding RNA Classification
Source: PLoS One. 2016 May 23;11(5):e0152238. doi: 10.1371/journal.pone.0152238 (PMC4877074; doi:10.1371/journal.pone.0152238)

**S9 Fig. The phylogenetic tree for the secondary structures of RNAs in S1 Fig based on the method by Feng *et al* [40].**


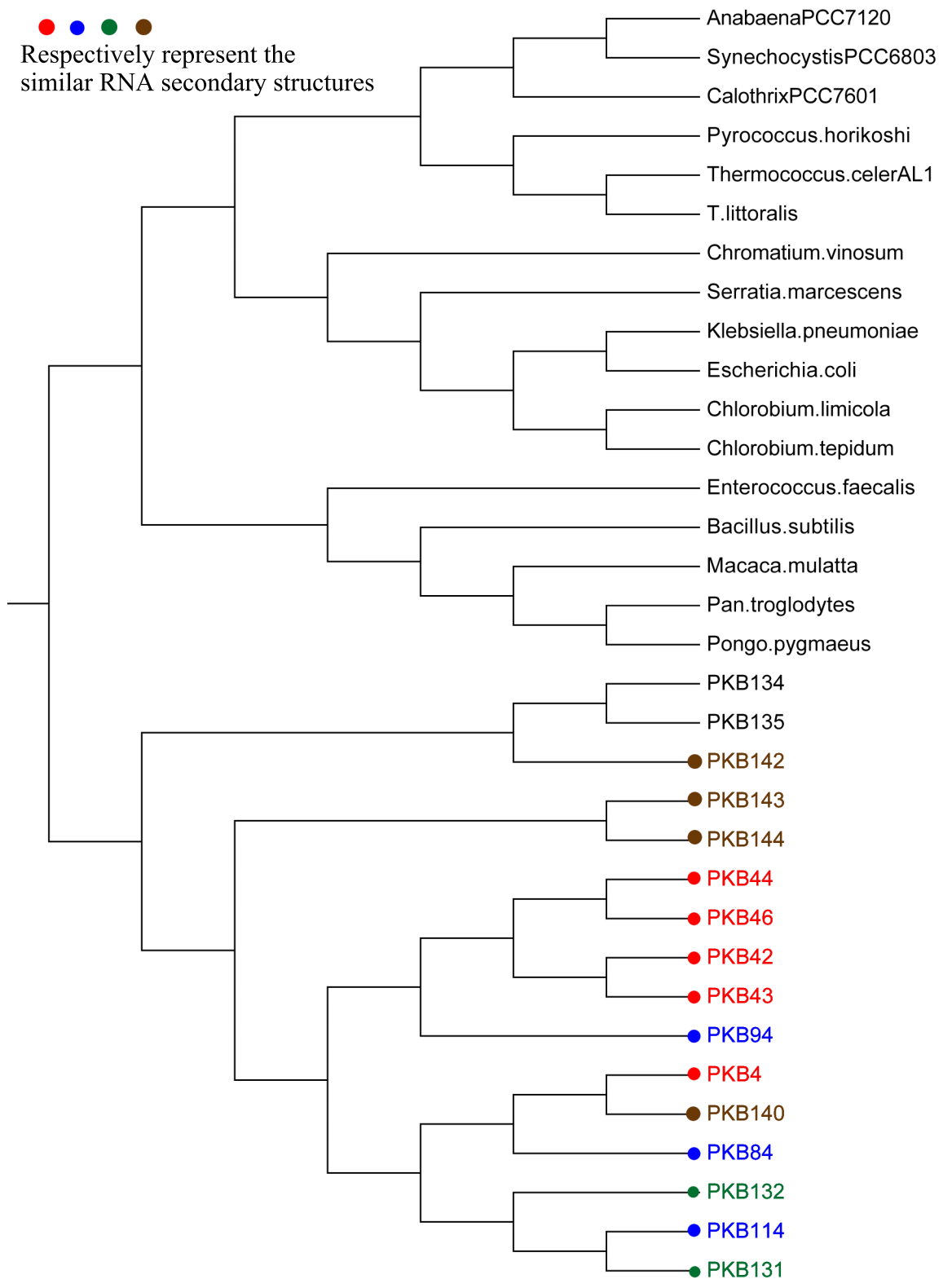

Supplement: S9 Fig — (DOC) [file pone.0152238.s009.doc]
