# Supplementary material for: A Dynamic 3D Graphical Representation for RNA Structure Analysis and Its Application in Non-Coding RNA Classification
Source: PLoS One. 2016 May 23;11(5):e0152238. doi: 10.1371/journal.pone.0152238 (PMC4877074; doi:10.1371/journal.pone.0152238)

**S15 Fig. The phylogenetic tree for the secondary structures of RNAs in S1 Fig based on the method by Li *et al* [24].**


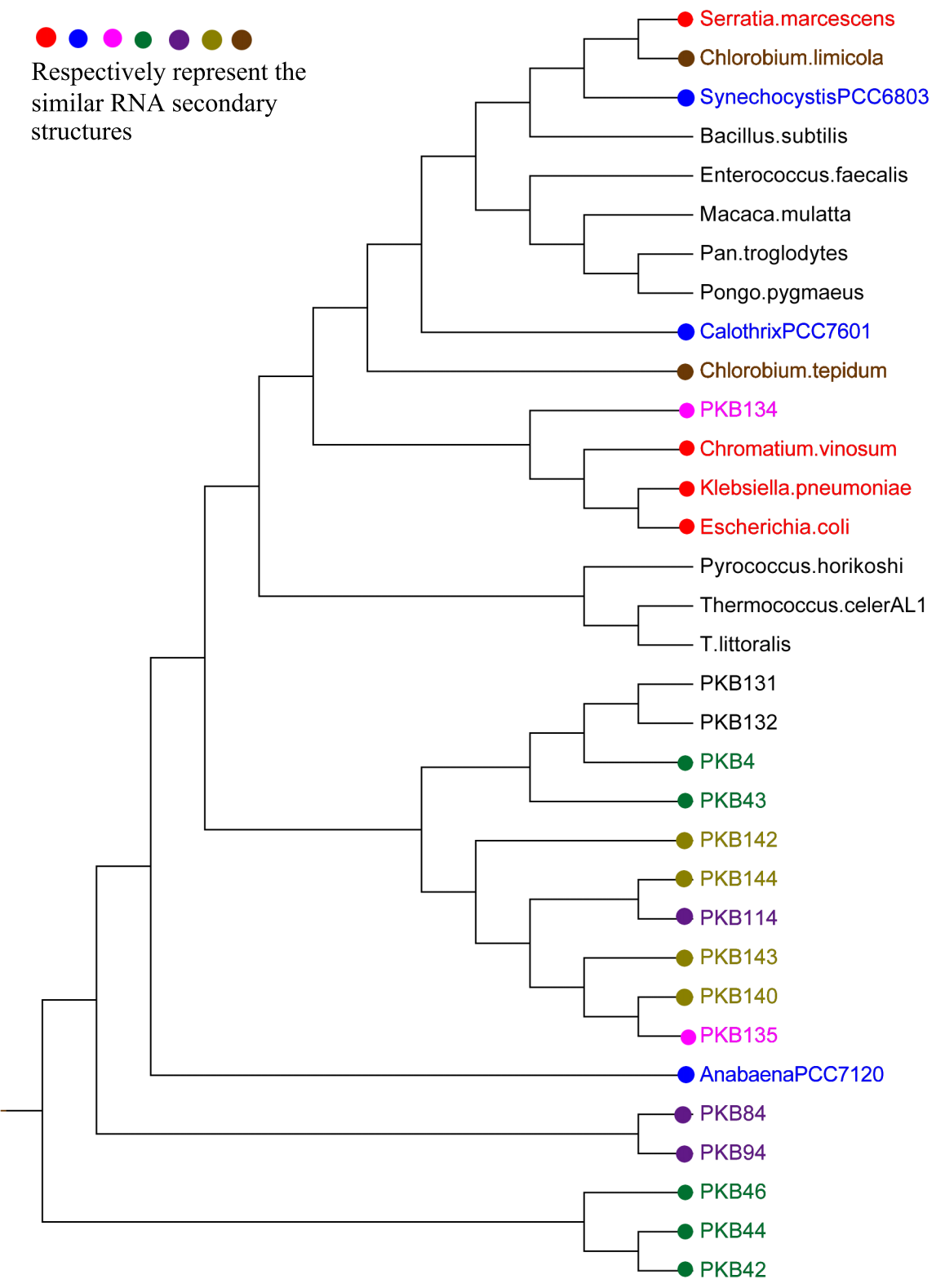

Supplement: S15 Fig — (DOC) [file pone.0152238.s015.doc]
