# Supplementary material for: A Dynamic 3D Graphical Representation for RNA Structure Analysis and Its Application in Non-Coding RNA Classification
Source: PLoS One. 2016 May 23;11(5):e0152238. doi: 10.1371/journal.pone.0152238 (PMC4877074; doi:10.1371/journal.pone.0152238)

**S19 Fig. The three phylogenetic trees for the secondary structures of RNAs in S1 Fig based on the method by Liu *et al* [39].** (A) The phylogenetic tree based on non-$A(A')$sequences. (B) The phylogenetic tree based on the non-$C(C')$ sequences. (C) The phylogenetic tree based on the non-$G(G')$sequences.

**(A)**

**
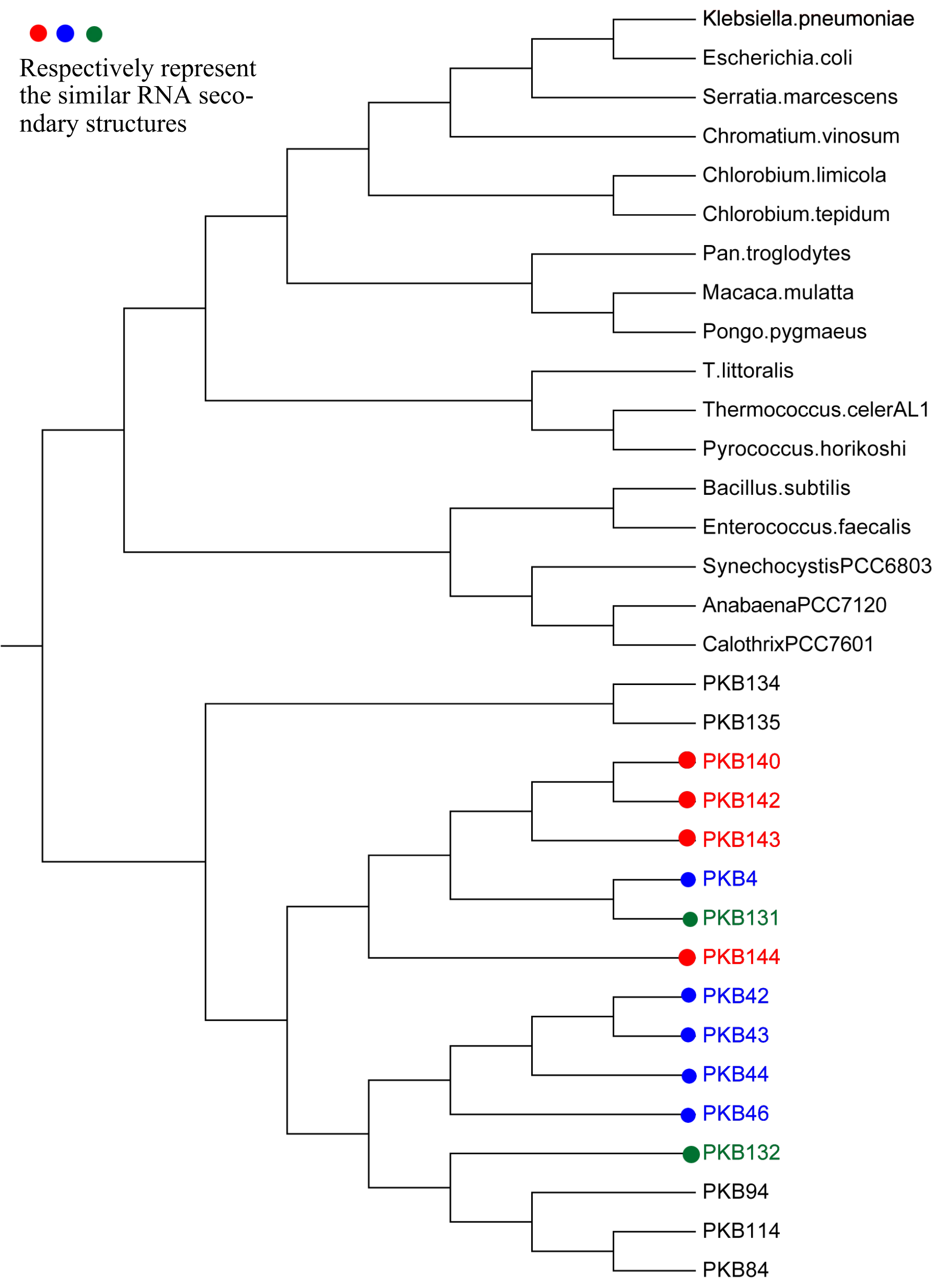
**

**(B)**


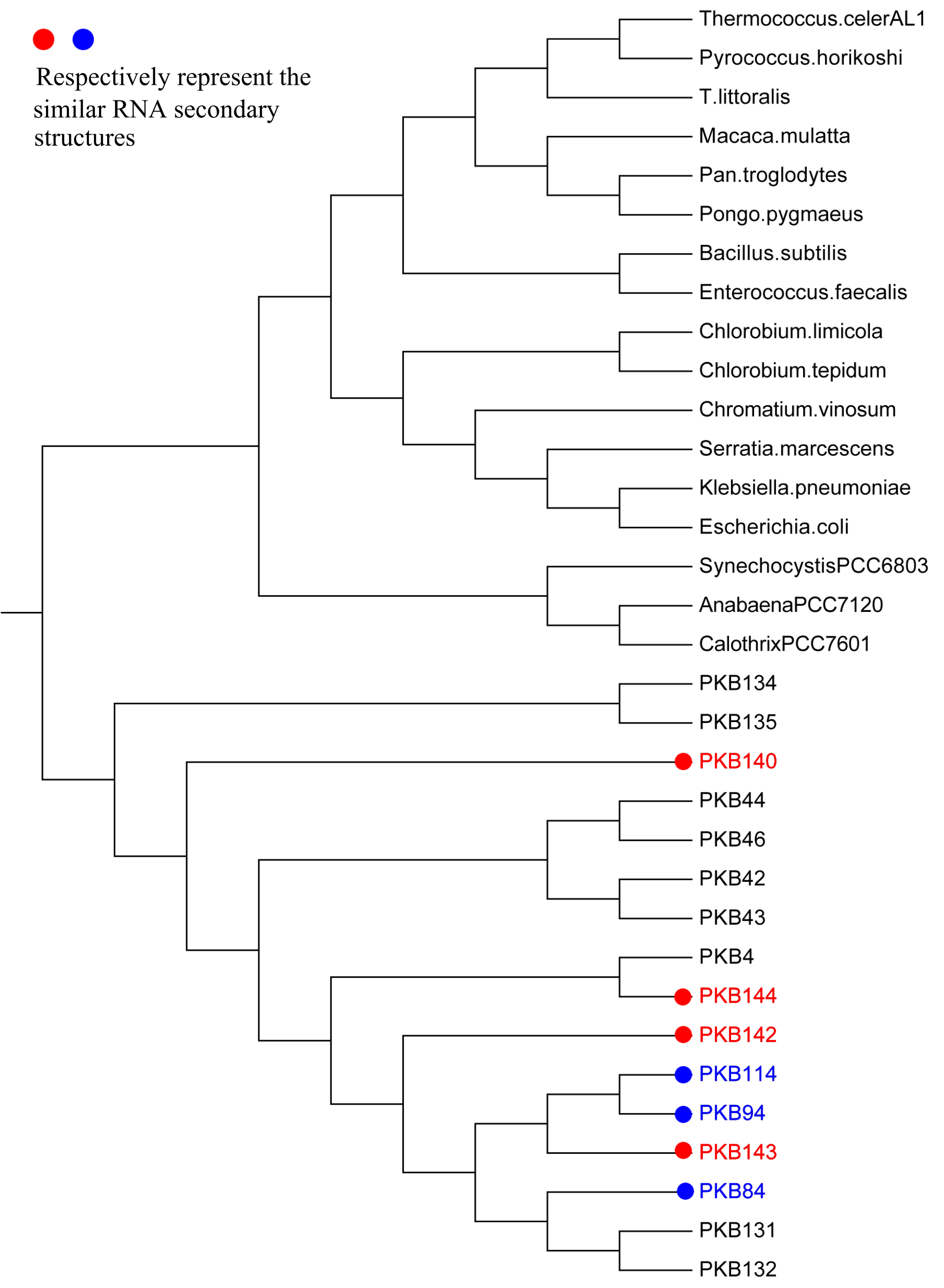


**(C)**


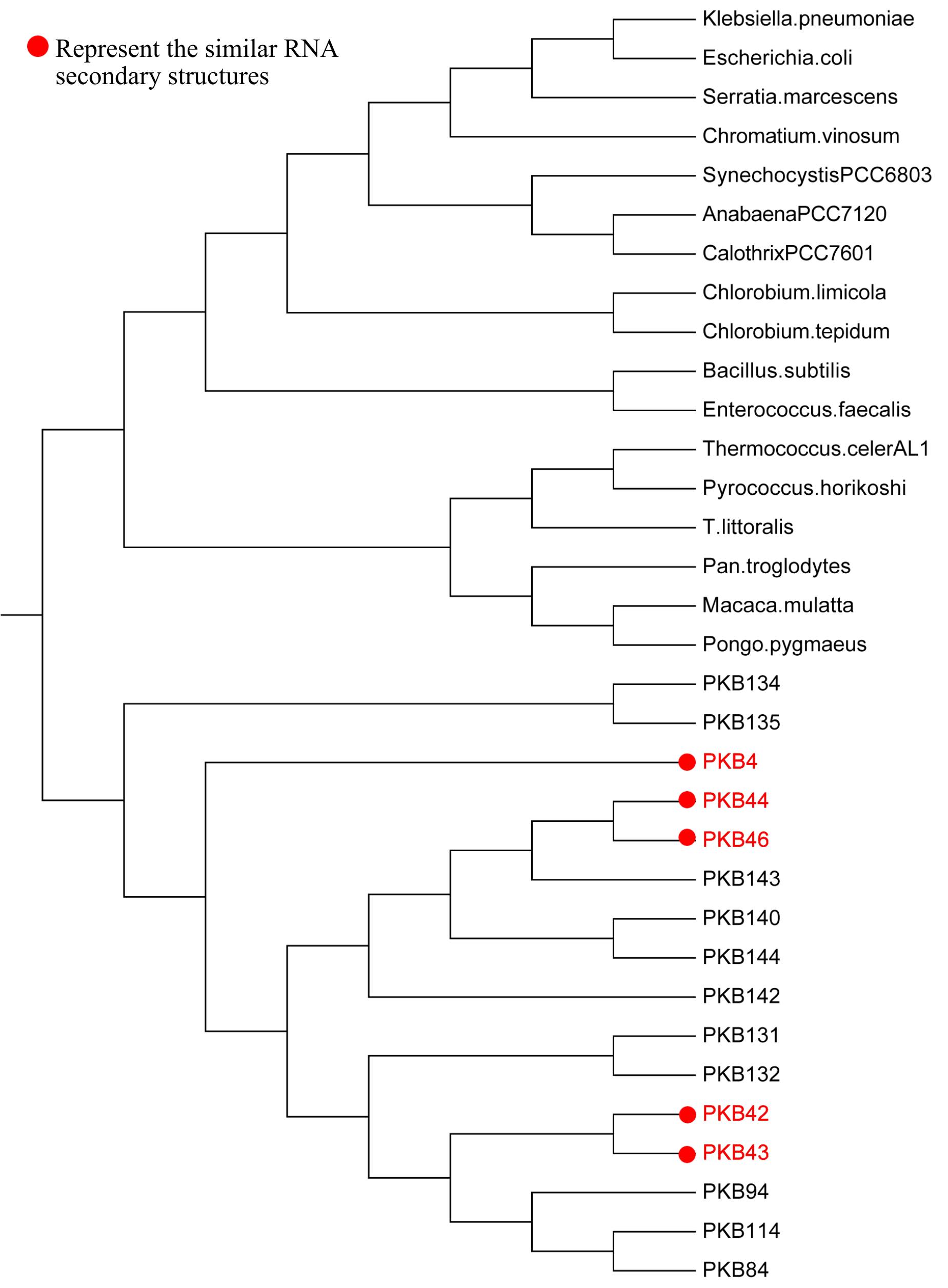

Supplement: S19 Fig — (A) The phylogenetic tree based on non-A(A′) sequences. (B) The phylogenetic tree based on the non-C(C′) sequences. (C) The phylogenetic tree based on the non-G(G′) sequences. (DOC) [file pone.0152238.s019.doc]
