# Supplementary material for: A Dynamic 3D Graphical Representation for RNA Structure Analysis and Its Application in Non-Coding RNA Classification
Source: PLoS One. 2016 May 23;11(5):e0152238. doi: 10.1371/journal.pone.0152238 (PMC4877074; doi:10.1371/journal.pone.0152238)
Supplement: S1 Table — (DOC) [file pone.0152238.s022.doc]

**S1 Table. The characteristic sequences for the secondary structures of RNAs in S1 Fig (A, G, C and U located in the base pairs are denoted as a, g, c and u).**

| **Species** | **RNA characteristic sequence** |
| --- | --- |
| PKB4 | GGGAAACUGGAAGGCggggcGAgcugcagccccAGUGAAUCAAAugcagc |
| PKB44 | GGGAAACGGGCAggcggCGgcgAccgccGAAACAACcgc |
| PKB46 | GGGAAACGGGAAggcggCGgcguccgccGUAACAAacgc |
| PKB42 | UUUAAAUGGGCGAgcggCAccgCccgcCAAAACAAAcgg |
| PKB43 | UUUAAAUGGGCAAgcggCAccguccgcCAAAACAAacgg |
| PKB140 | UuugcucauUGGUuugcggaaacCucUCacgUGUGAcguUgaAguuucuaugggcagUAAUUCUgcaaG |
| PKB142 | UuuuguuuUGGUcuuccgaacgCAUacgUuagUGUGAcuaCcguuguucgaaacaagUAAAACAggaagG |
| PKB143 | UugucguuGGGAuggaacgcAAUUaaauacaugUGUGAcguguauuugcgaacgacgUAAUUAUUuuucaG |
| PKB144 | UuguguauaUGGAucaugcggAUAAAguuauacuGGUGCaguauaacccguuauacacaUAAAAUUaugaG |
| PKB114 | UagugUUUAccguuccacuUAAAUCgAaacggA |
| PKB84 | CagugUUUUUcccuccacuUAAAUCgAagggU |
| PKB94 | CagugGUUGUucguCcacuUAAAUAUAacgaUU |
| PKB131 | cgcucAAcucauggagcgCAAGAcgaauaGCUACAuauucgAcaugag |
| PKB132 | cgcucAAccucagagcgCAAGAgucgaacGAAUACAguucgacAugagg |
| PKB134 | UCUAggugccuUUgagaguUAcucuuugcucucUUCGGAAgaacccUUAGggguucGUgcaugggcuugCAUAGcaagucuUAGAaugcggguaccGuacaguguUGAAAAacacuguaAAUCUCUAaaagagACCA |
| PKB135 | AAgauaucuGUaccggucUUcuccccgacgguUUCAAuccauAgcuucaCGUUGuguagcGUAGguggaagguaucgugugguguAUAAacaccacauAGUCUCUUAggggagACCA |
| Anabaena PCC7120 | AgggagagaguAggCGuUggcgguuGcagaCcaguuaGCUuaacugAuuugAGGAAAGUCCGGAcucccGAAAGACCAgacUugcuggaUAACGuccagugcgaGCGAucguGaggAUAguGCCACAGAAAAAUAccgccaagauugggGACugggGACUAgggguuggggaCuGgggaagAAAcuucccaAucccuaaucccCCAUAcccaAUAcccaaCucuugguAAggGUGCAAAggugcgGUAAGAGcgcaccAGcaguaucGAGAgguacugGCUCGGUAAaccccgguugggagCAAggcCGAAgaacuAUgguUGGUCUUUUaccaguucCGCUAUCAGAGAgccGCUAGAggcguuugGUAAcaaacgucCCAGAUAGAUaaucgcccucguguaaaGCAAuuuacacAgagAACAGAACCCGGCUUACCaccAacucucuccuCUUU |
| Calothrix PCC7601 | AAggagagaguAggCGuaggcgguuGcagaucaguCACAAAAacugAauuugAGGAAAGUCCGGGcucccGAAAGACCAaacUugcuggaUAACGuccagugcgaGCGAucguGaggAUAguGCCACAGAAAGAUAccgccaauuAgucauuAguCAuuugucAuuAgucAuuugGAAAAACAcaaaGgacAaaGgacaaaGAacAaaugacCAaauugguAAggGUGCAAAggugcgGUAAGAGcgcaccAGcaacgucGAGAggcguugGCUCGGUAAaccccgguugggagCAAggcGAAAGgaacuAUgguUGGUCUUUUaccaguucCGCUAAAACAGAgccGCUAGAggcguuugGUAAcaaacgucCCAGAUAGAUaaccgcccucauguagagaAguuaCUUGuaacAuuucuacauUCgagAACAGAACCCGGCUUACuaccAacucuuuccCUUUUU |
| Synechocystis PCC6803 | gagaguuagGgagggaguuGcggauuccugUCAcagggAaucugAGGAAAGUCCGGGcuuccCAAAGGCCAaacUugcugggUAACGcccagugcgcGCGAgcguGaggACAguGCCACAGAAAAAUAccgccCuuuuuagAAAAcagCAaccaguAAACaguuaAcagggUUUuucugCugacuAacugguAAcugACCAcugaaaagguAAggGUGCAAAggugcgGUAAGAGcgcaccAGcaguaucGAGAgguacugGCUCGGUAAaccccgguuggaagCAAgguCGGAGgggcaAAggUUGGUCUUUUUccugcccCAUGAUUGGUGGAAccGCUUGAggaauuugGUAAcaaauuucCCAGAUAGAUaacuccccAagggugcCUCgcauccuGgAACAGAACCCGGCUUACgacuaacucucUUUUUUU |
| Chromatium vinosum | ggagucggccagacagucGcuuccgucCUGGUgacgggggAGGAAAGUCCGGGcuccAUAGGGCAgggugccaggUAACGccuggggggcGAGAguccAcggAAAguGCCGCAGAAAAGAUAccgccGACcccgucCUCGgacgggAgguAAggGUGAAAUggugcgGUAAGAGcgcaccGcgcuggugGCAAcaucagugGCAGGGUAAaccccacccggagCAAgaccAAAUAGgggaacUCgcggCUUCGAccgcAgcgcgcGGCCCgcgcguguucccGGGUAggucGCUAGAggcgugcgGCGAcgcacgucCCAGAUGAAUgacugucCACGACAGAACCCGGCUUAuCggccgacuccCUUC |
| Klebsiella pneumoniae | gaagcugaccagacagucGccGcuucgucgucGuccuccUUCGgggggagacgGgcggagGggAGGAAAGUCCGGGcuccAUAGGGCAAggugccaggUAACGccugggggguGUCACGacccAcgaCCAguGCAACAGAGAGCAAAccgccGAuggcccgcGCAAgcgggAucagguAAggGUGAAAGggugcgGUAAGAGcgcaccGcgcggcugGUAAcagUccgcgGCACGGUAAacuccaccCggagCAAggccAAAUAGggguucAUAAgguacGGCCCguacugaacccGGGUAggcuGCUUGAgccagugaGCGAuugcuggcCUAGAUGAAUgacugucCACGACAGAACCCGGCUUAuCggucaguuucACCU |
| Escherichia coli | gaagcugaccagacagucGccGcuucgucgucGuccucUUCGggggagacgGgcggagGggAGGAAAGUCCGGGcuccAUAGGGCAgggugccaggUAACGccuggggggGAAAcccAcgaCCAgugcaACAGAGAGCAAAccgccGAuggcccgcGCAAgcgggAucagguAAggGUGAAAGggugcgGUAAGAGcgcaccgcgcggcugGUAAcagUccgugGcACgguAAacuccacccggagCAAggccAAAUAGggguucAUAAgguacGGCCCguacugaacccGGGUAggcuGCUUGAgccagugaGCGAuugcuggcCUAGAUGAAUgacugucCACGACAGAACCCGGCUUAuCggucaguuucACCU |
| Serratia marcescens | ggaguugaccagacagucGccGcuucauugCcGuccucUUCGggggagAcaGauggagGggAGGAAAGUCCGGGcuccAUAGGGCAgggugccaggUAACGccugggaggcGCAAgccuAcgaCUAguGCAACAGAGAGCAAAccgccGAuggcccgcGCAAgcgggAucagguAAggGUGAAAGggugcgGUAAGAGcgcaccGcgcggcugGUAAcagUucgugGCACGGUAAacuccacccggagCAAggccAAAUAGggguucACAUgguacGGCCCguacugaacccGGGUAggcuGCUUGAgccagugaGCGAuugcuggcCUAGAGGAAUgacugucCACGACAGAACCCGGCUUAuCggucaacuccCUC |
| Chlorobium limicola | aaaccgCaagugugcagucGcuguauggcUUGACgcugugcagAGGAAAGUCCGAAcuucACAGGGCAgggUgccggucGAgaacCUGguucAAggccgggggcagcgguGCAAaccgUcugucAcagAGAgugcaACAGAAAGCAAAccgcCccggcUCCGgccggAguAAggGUGAAAAggCgguGUAAGAGaccAccaggugcgUcaGCAAugCcguacGcuAugaAAaccuccccgaagCAAggccAAAUAUggaagcuuuuuccGCAAggaaagaAggguUGCCCgcccAACguuuccGGGUAggccGCAUCAGAUAAAUgacugcagCUucaucaCUCGAugaugaUUcACAGAAUUCGGCUUAcAgcuucgguuuCAGC |
| Chlorobium tepidum | aaaccgCaagugugcagucGcuguaugguAUAaccaugcagAGGAAAGUCCGAAcuucACAGGGCAgggUgccggucgagAACGUUUGuucAAgaccgggggcagcggcGCAAgcugUcugucAcagAGAguGCAACAGAAAGCAAAccgcuccggCUUAAAccggaguAAggGUGAAAAggGgguGUAAGAGaccAccAGgcaggUcaGCAAugCccugcGCUAUGAAAaccuccccgaagCAAggccAAAUAAggaagcauuuccuGCAAgggaagAAggguUGCCCgcccAAuguuuccGGGUAggccGCAUCAGAUAAAUggcugcaAcaucacuaCUUGAuggugaugGACAGAAUUCGGcuuacAgcuucgguuu |
| Thermococcus celer | uaggcgagggggcugggggcUGUcgggCUCGUAcccgAGGAAGUUCCGCCcaccGCACCGGGGccgcggugccGCAAggcaccucccGAGAgggagggCAACGGCACAGAAACGACAcgucccucgggGGAUGUGGAUGAAAgcggAGAAggcuccugGCGAcaggagccGAGCUAACccgaAGACAAUcccgaggggAGcgGUGAAACGGCCGUcccgcggggugCAAggccGAGUUAgggccAAUGAGUUCCCGGUGUGAggcccGUGGUAggccGCUUAGUCGAAUgcccccGCAGGUACAGAAGGCGGGCUAUagcccccucgccua |
| T. Litoralis | gggggcuggggcCCUcggguAUUUGacccGAGGAAGUUCCGCCcaccGCACCGGGGccgcggugccGCAAggcaccucccGGAAgggagggCAACGGCGCAGAAACGACAcgucccucgggAAAUGUGGAUGAAAgcggUGAAggcucccgGCGAcgggagcuGAGUUAAcccgCAGACAAUcccgaggggAGcgGUGAAACGGCCGUCccgcggggugCAAggccGAGAUAggggcUAUGAGUUCCCGGUGUGAgccccGUGGUAggccGCUCAGUCGAAUgccccAUUAAUACAGAAGGCGGGCUAUagcccccU |
| Pyrococcus horikoshii | uaggcgagggggcugggggcCCUcggggUGCucccgAGGAAGUUCCGCCcaccGCACCGGGGccgcggugccGCAAggcaccucccGAGAgggagggCAACGGCACAGAAACGACAcgccccucgggGGAUGUGGAUGAAAgcggUGAAggcuccugGUGAcgggggccGAGUUAACccgcAGACGAUcccgaggggAUcgGUGAAACGGCCGUcccgcggggugCAAggccGAGUUAgggccGAUGAGUUCCCGGUGUGAggcccGUGGUAggccGCUUAGUCGAAUgcccccGUAGUACAGAAGGCGGGCUAUagcccccucgccua |
| Bacillus subtilis | GUUCuuaacguucggguaaucGcugcagauCUUGAaucuguagAGGAAAGUCCAUGcucgcacggUgcUGAGAUgcccguAGUGUucgUgccuagcGAAGUCAUAAgcuagggcagucuUUAGaggcugAcggCaggaAAAAAgccUACgucUUCGgauAUggcUGAGUAuccuUGAAAgugccACAGUGACGAAGucucacuaGAAAuggugagaGUGGAACGCgguAAaccccUcgagcgagAAAcccAAAUUUUGGUAgggGAAccuucuuAACGGAAUUCAACGGAgagaaggacagaaUGCUuucuguAGAUAGAUgauugccGccUgaguacgagguGAUGAgccgUUUGCaguacgaUggAACAAAACAUGGCUUAcAgaacguuagACCACUU |
| Enterococcus faecalis | AUuuguaauuuucggguGaucGcgguuugcUUUUgcaagcugAGGAAAGUCCAUGcucgcacaagcUGAGAUgcuuguAGUGUucgUgcuuagcGAAAUCAUAAgcuaagguacucuUUUagaguaAcggCaggaAAAAUgacUAAgguUUCGacuAUgucAAAGUAuccuUGAAAguGCCACAGUGACGAAGcgaugUggGAAAcuCcaucgGUGGAACGCGGUAAaccccUcgagcgagCAAcccAAACAAUAAUAgggGCGcucuucuAAAGGAAAUGAACGAGUagaagaggcagagUUUAcucugcAGAUAGAUgauGaccGucaccaauuuuuCCUGaagaauuggugaUACAGAACAUGGCUUAuAgaaaauuacaaGUAAU |
| Macaca mulatta | auAgggcggagggAAGcucaUCAGUggggCCACGUGCUgagugCGUCCUGUcacucCACUCCCAUGUcccuUGGGAAGGUCUGAGACUAGggccAGAGGCggcccuAACagggcucucccugAUCUUcggggaggugaguUCCCAGAGAAUGGGgcuccgCgcgaggUCAGAcugggcaGGAGAugccGuggACCCcgccCUucgggGAGGGGcccggCggAugCCUCcuuugccggagcUUGGAACAGacucacggccAGCGAAGUGAGUUCAAUGGCugaggugagguAcccCGGAGgggaccucauAACCCAAUUCAGACUACUCuccuccgcccauU |
| Pan troglodytes | auAgggcggagggAAGcucaUCAGUggggCCACGAGCUgagugCGUCCUGUcacucCACUCCCAUGUcccuUGGGAAGGUCUGAGACUAGggccAAAGGCggccGUAACAGggcucucccugAGCUUcggggaggugaguUCCCAGAGAACGGGgcuccaCgcgaggUCAGAcugggcaGGAGAugccGuggACCCcgccCUucgggGAGGGcccggCggAugCCUCcuuugccggagcUUGGAACAGacucacggccAGCGAAGUGAGUUCAAUGGCugaggugagguAcccCGCAGgggaccucauAACCCAAUUCAGACUACUCuccuccgcccauU |
| Pongo pygmaeus | auAgggcggagggAAGcucaUCAGUggggCCACGUGCUgagugCGUCCUGUcacucCACUCCCAUGUcccuUGGGAAGGUCUGAGACUAGggccAGAGGCggcccuAACagggcucucccugaGCUucggggaggugaguUCCCAGAGAACGGGgcuccgCgcgaggUCAGAcugggcaGGAGAugccGuggACCCcgccCUucgggGAGGGGcccggCggAugCCUCcuuugccggagcUUGGAACAGacucacggccAGCGAAGUGAGUUCAAUGGCugaggugagguAcccCGCAGgggaccucauAACCCAAUUCAGACUACUCuccuccgcccauU |
