# Supplementary material for: A Dynamic 3D Graphical Representation for RNA Structure Analysis and Its Application in Non-Coding RNA Classification
Source: PLoS One. 2016 May 23;11(5):e0152238. doi: 10.1371/journal.pone.0152238 (PMC4877074; doi:10.1371/journal.pone.0152238)
Supplement: S2 Table — (DOC) [file pone.0152238.s023.doc]

**S2 Table. The characteristic sequences for the secondary structures of RNAs in S2 Fig (A, G, C and U located in the base pairs are denoted as a, g, c and u).**

| **Species** | **RNA characteristic sequence** |
| --- | --- |
| RF00001.Methanolobus.tindarius | ggagUUuggcggcCAUAgcggcgggGCAAuuccuguACCCAUCCCGAACacagAAgauAAGcccgccUgcGuucuauaCUGuacuaaagUauGAGAgucuuugggaAAuauggaaCgcugccaUcucc |
| RF00001.Pyrobaculum.aerophilum | gccccuAAcccggcCAUAggcgccgguGAUAcgcccggUCUCAUCAGAACccggAAgcuAAGgccggcgccGcgcucggGAGuacugggcUccGCGAgggcccgggaAAccggcgugcugggAgggggcUU |
| RF00001.Pyrodictium.occultum | uggcccgacccggcCAUAgcggccggGCAAcacccggACUCAUGUCGAACccggAAguuAAGccggccgcGuugggggAUGcuguggggUccGCGAggccccgcagCGcccccaagccgggAucgggccG |
| RF00001.Thermococcus.celer | gguAcggcgguCAUAgcggcgggGUCAcacccggUCUCGUUUCGACCccggAAguuAAGcccgccAgcGaucccggUUGuacugcccUccGGGAgggggcgggaAGccggggaCgccgccggccACU |
| RF00025.AF399707.1 | UUAUAcccgcUUAAUUCAUUcagAucuGUAAUagaAcugUCAUUCAACCCCAAAAAUCUAGUGCUGAUAUAACCUUCaccaauuaGGUUCAAAuaagugguAAUgcgggACAAaagacUaucGAcauUugauaCACUAUUuaucaauggaugucuuAUUUUUUUU |
| RF00025.U22349.1 | UUAUAcccgcAUAUCAUUcagAcccUUUAUggaAcugUCAUUCAACCCCAAAAAUCUAGUGCAAAUAUUGUCUUAauuacuuaGGCUAAAAGAAAuaaguauuAAAgcgggACaaggcaucGAcauUugauaCAAAUAUUgaucaauggaugucuuAUUUUU |
| RF00025.U22354.1 | UUAUAcccgcACAUUCACUcaaAucuGUAAUagaCuugUCAUUCAACCCCAAAAAUCUAGUGCAAAUAUUACCUUCgccaauuaGGUACAAuuaaugguUCUCgcgggACAAaagacUaucGAcauUugauaCAUUAUUuaucaauggaugucuuAUUUUUCUU |
| RF00374.AF019230.1 | GUcUgUAcuaguuGGCCgacuagCUcUgUACCUGgcggACccgUgguggaacuGACGaguucGGAAcacccggccgcAAcccugggaGACGucccggggAC |
| RF00374.AF033811.1 | GUcGgUAcuaguuAGCUaacuagCUcUgUAUCUGgcggACccgUgguggaacuGACGaguucGGAAcacccggccgcAAcccugggaGACGucccagggAC |
| RF00374.AF411814.1 | GUcUgUAcuaguuGGCCgacuagCUcUgUAUCUGgcggACccgUgguggagcuGACGaguucGGAAcacccggccgcAAcccugggaGACGucccagggAC |
| RF00374.AJ583798.1 | GUuUgUAcuaguuGGCCgacuagCUcUgUACCUGgcggACccgUgguggaacuGACGgguucGGAAcacccggccgcAAcccugggaGACGucccaaggAC |
| RF00374.J02266.1 | GUcUgUAcuaguuAGCUaacuagCUcUgUAUCUGgcggACccgUgguggaacuGACGaguucGGAAcacccggccgcAAcccagggaGACGucccagggAC |
| RF00374.K02729.1 | GUcUgUAcuaguuGGCCgacuagCUcUgUAUCUGgcggACccgUgguggaacuGACGaguucGGAAcacccggccgcAAcccugggaGACGucccggggAC |
| RF00109.AB169770.1 | UCCAuaucuuaaaGAAACAGCuuuCAAgugcCuuuCugCAGUUUUUcaggagcgcaagauaGAUU |
| RF00109.K00927.1 | UUCGuaucuuaagGAAACAGCuuuCAAgugcCuuuACugCAGUUUUUcaggagcgcaagauaGAUU |
| RF00109.M18895.1 | UCCAuaucuuaaaGAAACAGCuuuCAAgugcCuuuCugCAGUUUUUcaggagcgcaaggaaUUAA |
| RF00163.AF229821.1 | cuuuccCUGAAGAgacgAAGUGAUCAAGAGAUUGAAGACGAGUGAACUAAUUUUUUUUAAUAAAAAGUUCACCACGACUCCUCCUUCUCUCACAagucGAAacucAgaguCggaaag |
| RF00163.AF404041.1 | cuuuccCUGAAGAgacgAAGUGAUCAAGAGAUUGAAGACGAGUGAACUAAUUUUUUUAUAAAAGUUCACCACGACUCCUCCUUCUCUCACAagucGAAacucAgaguCggaaag |
| RF00163.AF404051.1 | cuuuccCUGAAGAgacgAAGUGAUCAAGAGAUCGAAGACGAGUGAACUAAUUUUUUUAAAUAAAAAGUUCACCACGACUCCUCCUUCUCUCACAagucGAAacucAgaguCggaaag |
| RF00163.X52041.1 | cuuuccCUGAAGAgacgAAGUGAUCAAGAGAUUGAAGACGAGUGAACUAAUUUUUUUAAUAAAAGUUCACCACGACUCCUCCUUCUCUCACAagucGAAacucAgaguCggaaag |
| RF00008.AF170504.1 | GauaaguCugugcUAAgcacaCUGAUGAgucuCUGAAAUGagacGAAacuuauC |
| RF00008.AJ005299.1 | UaagaguCugugcUAAgcacaCUGAUGAaucuCUGAAAUGagacGAAacucuuG |
| RF00008.AJ005300.1 | UaagaguCuguggUAAgcacaCUGAUGAgucuCUGAAAUGagacGAAacucuuG |
| RF00008.AJ005302.1 | AaagaguCugugcUAAgcacaCUGAUGAgucuCUGAAUUGagacGAAacucuuG |
| RF00008.AJ241845.1 | GaugaguCugugcUAAgcacaCUGAUGAgucuCUGAAAUGagacGAAacucauG |
| RF00008.M83545.1 | GaagaguCugugcUAAgcacaCUGACGAgucuCUGAGAUGagacGAAacucuuC |
| RF00094.AB037947.1 | GUGGCCGGCAUGgccccagccUCCUCGCUggcGCCGGCUGGGCAAcgauccgaggggaCUGuCccucucGagAaucgGCAAAuggggcCCC |
| RF00094.L22063.1 | GUGGCCGGCAUGgccccagccUCCUCGCUggcGCCGGCUGGGCAAcgauccgagggagCUAcUccucucGagAaucgGCAAAuggggcCCC |
| RF00094.M84917.1 | GUGGCCGGCAUGgucccagccUCCUCGCUggcGCCGGCUGGGCAAcauuccgaggggaCCGuCcuccucggUaaugGCGAAugggacCCA |
| RF00164.AF111997.1 | CagugcCGGGgccAcgcgGAGUAcgAUcgAGgguACAgcacaA |
| RF00164.L06253.1 | CagugcCGGGgccAcgcgGAGUAcgAUcgAGgguACAgcacuA |
| RF00164.U52595.1 | CagugcCGGGgccAcgcgGAGUAcgAUcgAGgguACAgcacuU |
| RF00164.Y15936.2 | UgaugcCGAGgccAcgccGGGUAggAUcgAGgguACAgcaucG |
| RF00165.D13096.1 | CUACUCUUGUACAGAAUGGUAAGCCAAgugUcaauaggAGGUACAAGCAAccuauugcauAUUA |
| RF00165.X51325.1 | CUAGUCUUAUACACAAUGGUAAGCCAgugGuaguaaaGGUAUAAGAAAuuugcuaCuauGUUA |
| RF00165.X66717.1 | CUACUCUUGUACAGAAUGGUAAGCACguguaauaggAGGUACAAGCCACccuauugcauAUUA |
| RF00524.U81811.1 | ACGUAGCUGAGCGCGUUUGAUAUAGUGAUAAAUGCCUGCugccugGGCUCAACAUCAAAUGcaggcaUAAACaucgcAACUAGCAACAAggaggAuggUUUUUAguacgUAGGcauugcggaacCCUCAACGUGAAGAAguucaGAUAGAgcaaugAAUcgugcAugcuagAGUCAUUGGUUCGACCuaguaUCUUUCGAAGAUUUccaUUccuucgcgauC |
| RF00524.U81814.1 | GCUUGAUCUCUGUAUUUGUUUCUAUUUUGAACAUUUGCCUGCuaccuuGGCAUAACAUCAAUaagguaCAACaucgcAAAAAGUCAUCAuaaggUgggUUUUAguacgUAGGcgcuguagaacUUAAUUguucuGAUAGAgcagcgAGUcgugcAugcuagUCUAACAUUUCUUGCUACcuaguaUCUUUAGAAGAUUUcccUCccuuagcgguC |
| RF00040.AE016759.1 | GuuuccguGUCCAuccuuGUUAAAACaagaaAUUUUacggaauAACCCAUUuugcccgaCCgaucAUCCacgCAgcAAUGgcGUAAGAcguAUUgaucUUucaggcagUUAGCgggcugcggguUGCAGUCCuuaccggUAGAUggaaaUAUUucuggAGAGUAAUACccaguCUGuuucuCUGAUAAUugcgcuguuuuucCGCAUgaaaaacgggcaaccgaCACUCUGCgccucUUUGAGCUGAcgauaacGUGAgguuggcgACGCGACUAGACACgaggcCAucgguUCACACccggaaaGGCGUUACUUUgcccgcagcuuAGUCGUCAA |
| RF00040.AL627269.1 | GuuuccgcACCGAAAcuuguUAAAacaaaAAGUUUAgcggaauAACCCAUUuugucugaCCgaucAUCAacgCAggAAUGgcGUAAGAcguAUUgauuUUucagacagUUAGCgggcugcggguUGCAGUCCuuaccggUAGAUaggauCUUCucuggAGAGUAAUGCccaggCUGcuuccCUGAUAAUugcGcuguguuucCGUAUgaaauacaggcaaccgaCACUCUGCgccucUUUGAGCUGAcgauaaccGUGAgguuggcgACGCGAAAUAGUCACgaggcCAucgguUCAUGCccgguaaGGCGUCACCAUgcccgcagcuuAGUCGUCAA |
| RF00040.D90744.1 | GuuuccguGUCCAuccuuGUUAAAACaagaaAUUUUacggaauAACCCAUUuugcccgaCCgaucAUCCacgCAgcAAUGgcGUAAGAcguAUUgaucUUucaggcagUUAGCgggcugcggguUGCAGUCCuuaccggUAGAUggaaaUAUUucuggAGAGUAAUACccaguCUGuuucuUUGAUAAUugcGcuguuuuucCGCAUgAAAAAcgggcaaccgaCACUCUGCgccucUUUGAGCUGAcgauaaccGUGAgguuggcgACGCGACUAGACACgaggcCAucgguUCACACccggaaaGGCGUUACUUUgcccgcagcuuAGUCGUCAA |
| RF00024.AF221913.1 | UUaauuagagggAUuggaagguuccGCUUAUGCUAACCCUAAUAUUGGGGGUCUGUUGAAAAccucuUUAAGAUAugcguguugUUUUAUUGGCUGACUUUcagcgcgcaUUGagaggAGUUGCUGCCCAGGACUAAAAAAUGUCAGCUGggaguCcuuccucucccuuauuucugccuCACAACCUGGAcucuuuaUUUAgcggugccccAUUUGUCGAGGCcgcagUcaguCUuguUCUUauaCGCUgcuguugcgAagagUUCGUcucuGUCAGCCUCCggggcaacgcCUUGAAUUuggagagCCUGGGAAUGUAACAAGggguaggGAAAAUAACGAgagcugAguugGCUUCUCCUGugcUGuuccUGAGCUGUggaaCUUgcaAUCGcagucggcucUGACACUU |
| RF00024.AF221921.1 | GGCGGGuugcgggagCugcgagcggccgUCUCGUCUAACCCUAAAGAGAAAggcguagguGCUUGgccuGGCGACUcgcccgcugUUUUUUGGCUGGCUUUcagcgggugAAgaggcCCAAGaccuaCcgccACCCACCGUCUAGUGUCUUAAGGGCACAAAGUCCUGCCCGCCACCCUUCGAGGAGCGAAACCCAAAAAAGUCAGCCCCuggccgCCUcucgcccucucgcaacccgcCUccgucccAgccucCAUCcccgcCCCAGGCcgcggUcgguCCgggCUUCUUCGGAAGUcccGUUgccgucgcgAagagUUCGCcucuGUCAGCCgcgggGCUUggggcCAgggacggGACCCUGUCCgcaggGAGAGAAACUggagccGggcccUCCACGgugccucccCGAGCUGUgggaUCugcacCCgggacuCgaaccCUACACUU |
| RF00024.AF221939.1 | GGGUUGUGGAGGGUGGGccugggaggggaagCggucaGUUUUUGUCUAACCCUAACUGAGAAGggcguaggcGCCGCgcuuuUGUUUCCcgcacgcugUUUUUUUCGCUGACUUUcagcgggcgGAaaagcCUCGgccuaccgccGUCCACCGUACAGUUUGGAGCAAACAAAAAAUGUCAGCUGCugacuUGCucgccccucccaggACccugcggUGGCUCgccuccuuAGcccccgCGUcccgcCUAGAGGCcgcggUcggcCCgggGCUUCUCCGGAGGCAcccAUUgccgucgcgAagaguUGGgcucuGUCAGCCgcgggUCCCUugggggGGCCGAgggCgaggcUCUGAccgcaggGAGAGAAACGGgagcAggucccCGCGCGCGgugcgcUucccUGAGCUGUgggaCuugcacCCgggacuGGgcucAGACACAU |
| RF00024.U85256.1 | GGGUUGCGGAGGGUGGGccugggagggguggUggccaUUUUUUGUCUAACCCUAACUGAGAAGggcguaggcGCCGUgcuuuUGCUCCCcgcgcgcugUUUUUCUCGCUCACUUUcagcgggcgGAaaagcCUCGgccugCcgcUUCCACCGUUCAUUCUAGAGCAAACAAAAAAUGUCAGCUGCuggccCGUucgccccucccgggGAccugcggCGGGUCgccugcccAgcccccgaACcccgcCUGGAGGCcgcggUcggcCCgggGCUUCUCCGGAGGCAcccACUgccaccgcgAagaguUGGgcucuGUCAGCCgcgggUCUCucgggggcGAgggcGagguUCAGGCCUUUCAGGccgcaggAAGAGGAACGgagcgagucccCGCGCGCGgcgcgAUucccUGAGCUGUgggacgugcACCCaggacucGgcucACACAUGC |
| RF00019.K01563.1 | ggcuggucCGAGUgcaguggUGUUUACAACUAAUUGAUCACAACCAGUUACAGAUUUCUUUGUUCCUUCUCCACUCccacugcUUCACUUgacuagccUU |
| RF00019.L27537.1 | ggcuggucCGAAAguaguggGUUAUCACAGAAAUUAUUACAGUUAGUUUCACUAACCUUUCUAAGUUCCACCccacugcUAACCUUgacuggucUU |
| RF00019.U34827.1 | gguuggucCGAGAguaguggUGUUUACAACUAAUUGAUCACAACCAGUUACAGAUUUCUUUGUUCCUUCUCCGCUCccacugcUUCACUUgaccagccUU |
| RF00030.AY090598.1 | aucgucaCUAAucgaagcUUACAAAAAUGgaguaaAGGAAUUUUUCUUCuuacucAAUAUUAAUgcuuuggGUUGAAAGUCUCCCACCAAuucaUAUGcggaAAACGuaaugagauuuaaaaaauUUAaauuCuuuaaauCAAcucauuaAgagaaauaucUUUAgauauUUCUCUUCuugACCCaguaCUCUguuACaggagcgcugguguuuucuUCGGUACUGGAuuccUUAUAUAUggaaUCUAAACCAUAGUUAugacgauUGcucuuuccCAUGCUggaucgagUAACCCAAUGGAGCU |
| RF00030.AY090601.1 | aucgucaCAAAucgaagcUUACAAAAUGgaguaaAAUUUUUUuuacucAGUAAUAUgcuuuggGUUGAAAGUCUCCCACCAAuucgUAUGcggaAAACGuaaugagauuuaaaaauuUUAaauuGuuuaaauCAAcucauuaAggaggaugccCUUGgguauUCUGCUUCuugACCUgguaCCUCuauuGCaggguacugguguuuucuUCGGUACUGGAuuccguUUGUauggaaUCUAAACCAUAGUUAugacgauUGcucuuuccCGUGCUggaucgagUA |
| RF00030.AY090604.1 | aucgucaCAAAucgaagcUUACAAAAUGgaguaaAAUUUUUUuuacucAGUAAUAUgcuuuggGUUGAAAGUCUCCCACCAAuucgUAUGcggaAAACGuaaugagauuuaaaaauuCUAaauuGuuuaaauCAAcucauuaAggaggaugccCUUGgguauUCUGCUUCuugACCUgguaCCUCuauuGCaggguacugguguuuucuUCGGUACUGGAuuccguUUGUauggaaUCUAAACCAUAGUUAugacgauUGcucuuuccCGUGCUggaucgagUAACCCAAUGGAGCU |
| RF00100.X04992.1 | ggaUgugagggCgaUCUggCUGCgacaucugucAccccaUUgaucgccAGGGUUGAUUCggcUgaucuggCUggCUAggcgggugucCccUucCUcccucacCGCuccAUGUGCGucccucccgaAgcUGCGCgcucggucgAAGAggaCGACCAuccCcgaUAgaggAggACCGGUCUUCGGUCAAGGGUAUACGAGUAgcuGCGcuCcccugcUAGAACcuccAAacaagCUCUCAAGGUCCAuuuguAggagAACguagggUagUCAagcUUCCAAGACUCCAGACACAUCCaaaUgaggcgcugcAUGUGgcaguCUgccuuUCuuu |
| RF00503.AF230358.1 | UAACUAGaucacaGAGAugugauGGAAAauaguugAugaguuguuuaAUUuuaagaAUUUUUAucuuaaUUAAGGAAggagugauUucAAUGGCACAAgaUaucauuucAACAAucggugacUUAGUAAAAUGgAuuaucgaCACagugaaCAAAuucacuAAAAAAUAagauGaauaaUUAAuuacUUUCAUUguaaAUuuguuaucuUCguauaguacuAAAAguaugAguuauuaagCCAUCCCAAcuuaauaaccauguAAAauuagcaagugAGUAAcauuugcuaguAgaguuAGUUUCCUUGgacucagugcuacguAUUUUUCUuaauuaucAUUACAgauaauuaUUUCuagcAUGUAAgcuaUCGuaaacaacAucgauuuaucaUUAUUugauaaauAAAAUuuuuuucaUAauuaauAACAUCCCCAAAAauagauugaaaaaauaacuguAAAACAUUCCCUUAAUAAUAAGUAGG |
| RF00503.AF288215.1 | UAACUAGauuacaGAGAugugauGGAAAauaguugAugaguuguuuaAUUuuaagaAUUUUUUAucuuaaUUAAGGAAggagugauUucAAUGGCACAAgaUaucauuucAACAAucagugacUUAGUAAAAUGgAuuaucgaCACagugaaCAAAuucacuAAAAAAUAagauGaauaaUUAAuuacUUUCAUUguaaAUuuguuaucuUCguauaguacuAAAAguaugAguuauuaagCCAUCCCAAcuuaauaaccauguAAAauuagcaagugAGUAAcauuugcuaguAgaguuAGUUUCCUUGgacucagugcuauguAUUUUUCUuaauuaucAUUACAgauaauuaUUUCuagcAUGUAAgcuaUCGuaaacaacAucgauuuaucaUUAUUugauaaauAAAAUuuuuuucaauuaauAACAUCCCCAAAAauagauugaaaaaauaacuguAAAACAUUCCCUUAAUAAUAAGUAUGGgucgugagcCCCUCCCAAgcucgcggc |
| RF00503.BX571856.1 | UAACUAGaucacaGAGAugugauGGAAAauaguugAugaguuguuuaAUUuuaagaAUUUUUAucuuaaUUAAGGAAggagugauUucAAUGGCACAAgaUaucauuucAACAAucggugacUUAGUAAAAUGgAuuaucgaCACagugaaCAAAuucacuAAAAAAUAagauGaauaaUUAAuuacUUUCAUUguaaAUuuguuaucuUCguauaguacuAAAAguaugAguuauuaagCCAUCCCAAcuuaauaaccauguAAAauuagcaagugAGUAAcauuugcuaguAgaguuAGUUUCCUUGgacucagugcuauguAUUUUUCUuaauuaucAUUACAgauaauuaUUUCuagcAUGUAAgcuaUCGuaaacaacAucgauuuaucaUUAUUugauaaauAAAAUUuuuuuucaauuaauAACAUCCCCAAAAauagauugaaaaaauaacuguAAAACAUUCCCUUAAUAAUAAGUAUGgucgugagcCCCUCCCAAgcucgcggc |
| RF00505.AE016760.1 | CUUCCGAUGUAGGCCCGUAUUCUUcgccuguacCACGGGUCGGUUUUAguacaggcgUUUUCUU |
| RF00505.AL627270.1 | CUUCCGAUGUAGACCCGUCCUCCUUcgccugcguCACGGGUCCUGGUUAgacGcaggcgUUUUCUG |
| RF00551.AF465792.1 | ggcgACacggauuCcagugcAUAucuuagugauACUCCAGUUAACUCCAUacuuuccCugCaauacgcuAUUCgccucagauguaUcuggGUGguugCuccACUAAGCCCAggaAuaAccAGccagUuacauuugaggcCAUUUGGGCUUAagcguauuCcaUggaaaguUGUCGUCCCAUAUUUCGAAAAUUAaauuccgAGCcagcaagaaaAucuuCucugUUacaauuugAcauagcuaAaaaCugUACUAaucaaaAUGAAaaauguuuCucuugggCguaauCUCAUACAAUGauuacccuUAaagaUCgaacauuuAAACAAUAAUAuuugauAUGAUAUUUUcaAuuuCuaUgcuaugCcaaagUgucugAcauAAUCAAACAuuugcacauucuUUGACCAagaauAguCAgcaaaUUGUAUUUUCAAUCAaugcagaccAUauguUCcaguUUCGgagauuuuuugcugCCAAAcggaauaCUUAUAAAAACCCACAUUCUAUUUACaucacuaagaAGAgcauugCaaucuguUUagcc |
| RF00551.M32123.1 | ggcgACacggauuCcagugcAUAucuuagugauACUCCAGUUAACUCCAUacuuuccCugCaauacgcuAUUCgccucagauguaUuuggGUGguugCuccACUAAAGCCCAggaAuaUccAGccagUuacauuugaggcCAUUUGGGCUUAagcguauuCcaUggaaaguUUUCUCCCCACAUUUCGGAAAUUAaauuccgAGCcagcaagaaaAucuuCucugUUacaauuugAcauggcuaAaaaCugUACUAaucaaaAUGAAaaauguuuCucuugggCguaauCUCAUACAAUGauuacccuUAaagaUCgaacauuuAAACAAUAAUAuuugauAUGAUAUUUUcaAuuuCuaUgcuaugCcaaagUgucugAcauAAUCAAACAuuugcacauucuUUGACCAagaauAguCAgcaaaUUGUAUUUUCAAUCAaugcagaccAUauguUCcaguUUCGgagauuuuuugcugCCAAAcggaauaCUUAUAAAAACCCACAUUCUAUUUACaucacuaagaAGAgcauugCaaucuguUUagcc |
| RF00551.M32124.1 | ggcgACacggauuCcagugcAUAucuuagugauACUCCAGUUAACUCCAUacuuuccCugCaauacgcuAUUCgccucagauguaUcuggGUGguugCuccAUUAAAGCCCAggaAuaUccAGccagUuacauuugaggcCAUUUGGGCUUAagcguauuCcaUggaaaguUUUCGCCCCACAUUUCGGAAAUUAaauuccgAGCcaucaagaaaAucuuCucugUUacaauuugAcauagcuaAaaaCugUACUAaucaaaAUGAAaaauguuuCucuugggCguaauCUCAUACAAUGauuacccuUAaagaUCgaacauuuAAACAAUAAUAuuugauAUGAUAUUUUcaAuuuCuaUgcuaugCcaaagUgucugAcauAAUCAAACAuuugcacauucuUUGACCAagaauAguCAgcaaaUUGUAUUUUCAAUCAaugcagaccAUauguUCcaguUUCGgagauuuuuugcugCCAAAcggaauaCUUAUAAAAACCCAAAUUCUAUUUACaucacuaagaAGAgcauugCaaucuguUUagcc |
