# Supplementary material for: A Dynamic 3D Graphical Representation for RNA Structure Analysis and Its Application in Non-Coding RNA Classification
Source: PLoS One. 2016 May 23;11(5):e0152238. doi: 10.1371/journal.pone.0152238 (PMC4877074; doi:10.1371/journal.pone.0152238)
Supplement: S3 Table — (DOC) [file pone.0152238.s024.doc]

**S3 Table. The comparison between our method and the other nine algorithms.**

| **Article name** | **Classification of RNA secondary structures of set II in S1 Fig** | **Classification of ncRNA secondary structures of set III in S2 Fig** |
| --- | --- | --- |
| Multi-scale RNA comparison based on RNA triple vector curve representation [26] | Unable to separate the complicated RNA secondary structures of RNase P database from RNA secondary structures with pseudo-knots of Pseud Base (see S3 Fig). | RF00024. AF221913.1 and RF00001. Thermococcus. celer are not assigned to the corresponding families (see S4 Fig). |
| A 3D Graphical Representation of RNA Secondary Structures [24] | Unable to separate the complicated RNA secondary structures of RNase P database from RNA secondary structures with pseudo-knots of Pseud Base (see S5 Fig A and B). | The classification of ncRNA secondary structures among the families of RF00024, RF00030, RF00025 and so on are in confusion (see S6 Fig A and B). |
| A condensed 3D graphical representation of RNA secondary structures [23] | Some dissimilar RNA secondary structures are closely related and that is unreasonable (see S7 Fig A and B). | The classification of ncRNA secondary structures among the families of RF00019, RF00505, RF00001 and so on are in confusion (see S8 Fig A and B). |
| A 3D graphical representation of RNA secondary structures based on chaos game representation [45] | Some dissimilar RNA secondary structures with pseudo-knots are closely related and that is unreasonable (see S9 Fig). | The classification of ncRNA secondary structures among the families of RF00165, RF00019 and RF00040 are in confusion (see S10 Fig). |
| On 3D graphical representation of RNA secondary structures and their applications [46] | Unable to separate the complicated RNA secondary structures of RNase P database from RNA secondary structures with pseudo-knots of Pseud Base (see S11 Fig A and B). | The classification of ncRNA secondary structures among the families of RF00025, RF00165, RF00505 and so on are in confusion (see S12 Fig A and B). |
| RNA secondary structure 3D graphical representation without degeneracy [47] | Bacillus. subtilis and Klebsiella. pneumoniae are dissimilar but closely related. PKB4 is similar with PKB44, PKB46, PKB42 and PKB43 but not closely related (see S13 Fig). | Good classification (see S14 Fig). |
| Analysis of similarity of RNA secondary structures based on a 2D graphical representation [22] | Some dissimilar RNA secondary structures are closely related and that is unreasonable (see S15 Fig). | The classification of ncRNA secondary structures among the families of RF00019, F00030, RF00024, RF00001 and so on are in confusion (see S16 Fig). |
| A 2D graphical representation of RNA secondary structures and the analysis of similarity /dissimilarity based on it [48] | Some dissimilar RNA secondary structures are closely related and that is unreasonable (see S17 Fig A and B). | The classification of ncRNA secondary structures among the families of RF00030, RF00025, RF00001, RF00040 and so on are in confusion (see S18 Fig A and B). |
| Comparing RNA Secondary Structures Based on LZ Complexity [44] | Some dissimilar pseudoknot secondary structures are closely related and that is unreasonable (see S19 Fig A, B and C). | RF00019. L27537.1 is assigned to the class of RF00001 (see S20 Fig A). |
| Our similarity measure | Good classification (see Fig 5). | Just one (RF00001. Methanolobus. tindarius) is assigned to the class of RF00374 (see Fig 6). |
