# Supplementary material for: A Dynamic 3D Graphical Representation for RNA Structure Analysis and Its Application in Non-Coding RNA Classification
Source: PLoS One. 2016 May 23;11(5):e0152238. doi: 10.1371/journal.pone.0152238 (PMC4877074; doi:10.1371/journal.pone.0152238)
Supplement: S1 Text — (DOC) [file pone.0152238.s025.doc]

# S1 Text. The proof of three properties

## Proof of Property 1

The definition of the maps and indicates that if $x_{1i}>0$, $y_{1i}>0$, $x_{2i}>0$, $y_{2i}>0$, then $g_{i}=A$; if $x_{1i}>0$, $y_{1i}>0$, $x_{2i}>0$, $y_{2i}<0$, then $g_{i}=C$; if $x_{1i}>0$, $y_{1i}<0$, $x_{2i}>0$, $y_{2i}>0$, then $g_{i}=G$; if $x_{1i}>0$, $y_{1i}<0$, $x_{2i}>0$, $y_{2i}<0$, then $g_{i}=U$; if $x_{1i}<0$, $y_{1i}>0$, $x_{2i}<0$, $y_{2i}>0$, then $g_{i}=A'$; if $x_{1i}<0$, $y_{1i}>0$, $x_{2i}<0$, $y_{2i}<0$, then $g_{i}=C'$; if $x_{1i}<0$, $y_{1i}<0$, $x_{2i}<0$, $y_{2i}>0$, then $g_{i}=G'$; if $x_{1i}<0$, $y_{1i}<0$, $x_{2i}<0$, $y_{2i}<0$, then $g_{i}=U'$. Therefore, any two of the three curves can uniquely determine the original RNA secondary structure, which makes the mapping on plan X-Y non-degenerative.

## Proof of Property 2

From **Fig. 2** in the main context, it is obvious that the distribution of points in $\left\{A',G',C',U'\right.\}$is dense while that in others is sparse, which indicates that the content of bases$\left\{A',G',C',U'\right.\}$is higher than the content of bases $\{A,G,C,U\}$ in the characteristic sequence of TSV-3. We can also immediately conclude that the$AU$-content is higher than the$CG$-content, the$AC$-content is similar to $CU$-content, and the$AG$-content is higher than the$CU$-content.

## Proof of Property 3

According to the definition of maps and , we can obtain $z_{1}^{1}=\frac{1}{n}\sum_{l=1}^{A_{n}+C_{n}} l$ and $z_{2}^{1}=\frac{1}{n}\sum_{l=1}^{A_{n}+G_{n}} l$, where $A_{n}$, $C_{n}$, and $G_{n}$ are the cumulative occurrence numbers of $A$, $C$, and $G$, respectively, in a characteristic sequence of the RNA secondary structure. $z_{1}^{1}>z_{2}^{1}$ implies $\frac{A_{n}+C_{n}}{n}>\frac{A_{n}+G_{n}}{n}$, and thus $c>g$. Similarly, $z_{1}^{1}>z_{3}^{1}$ implies $c>u$; $z_{1}^{2}>z_{3}^{1}$ implies $g>a$; $z_{1}^{3}>z_{2}^{3}$ implies $c'>g'$; $z_{1}^{3}>z_{3}^{3}$ implies $c'>u'$; $z_{1}^{3}>z_{2}^{4}$ implies $a'>u'$and others in the same way. Therefore, the variables of $z_{1}^{1}$, $z_{1}^{2}$, $z_{1}^{3}$, $z_{1}^{4}$, $z_{2}^{1}$, $z_{2}^{2}$, $z_{2}^{3}$, $z_{2}^{4}$, $z_{3}^{1}$, $z_{3}^{2}$, $z_{3}^{3}$, and $z_{3}^{4}$ indicate the distribution of any two bases frequencies of $a',g',c',u'$ or $a,g,c,u$.
