# Supplementary material for: A Dynamic 3D Graphical Representation for RNA Structure Analysis and Its Application in Non-Coding RNA Classification
Source: PLoS One. 2016 May 23;11(5):e0152238. doi: 10.1371/journal.pone.0152238 (PMC4877074; doi:10.1371/journal.pone.0152238)
Supplement: S1 Software — (ZIP) [file pone.0152238.s021.zip › S1 Software. RNAfeatureGenerator/S1 Software. RNAFeatureGenerator.doc]

**Software:** RnaFeatureGenerator.exe can generate the 36-dimensional vectors of any RNA secondary structure.

**Usage**

- Put (1) RnaFeatureGenerator.exe and (2) the sequence file “Sequences.txt” in the same fold and double-click RnaFeatureGenerator.exe. The results are saved in an automatically generated file “Results.txt”
- Sequences.txt format: name:sequence

Example:

AIMV-3:AUGCucaugcaAAACugcaugaAUGCcccUAAgggAUGC

APMV-3:AAUGCccacaacGUGAAguuguggAUGCcccGUUAgggAAGC

We also implemented our methods together with the other nine similarity analysis methods in Matlab. The codes are available upon request.
